# Thermosensitive hydrogel releasing nitric oxide donor and anti-CTLA-4 micelles for anti-tumor immunotherapy

**DOI:** 10.1038/s41467-022-29121-x

**Published:** 2022-03-18

**Authors:** Jihoon Kim, David M. Francis, Lauren F. Sestito, Paul A. Archer, Margaret P. Manspeaker, Meghan J. O’Melia, Susan N. Thomas

**Affiliations:** 1 Parker H. Petit Institute for Bioengineering and Bioscience, Georgia Institute of Technology, 315 Ferst Dr NW, Atlanta, GA 30332 USA; 2 George W. Woodruff School of Mechanical Engineering, Georgia Institute of Technology, 315 Ferst Dr NW, Atlanta, GA 30332 USA; 3 School of Chemical and Biomolecular Engineering, Georgia Institute of Technology, 315 Ferst Dr NW, Atlanta, GA 30332 USA; 4 Wallace H. Coulter Department of Biomedical Engineering, Georgia Institute of Technology, 313 Ferst Dr NW, Atlanta, GA 30332 USA; 5 Wallace H. Coulter Department of Biomedical Engineering, Emory University, 201 Dowman Drive, Atlanta, GA 30322 USA; 6 Winship Cancer Institute, Emory University School of Medicine, 1365-C Clifton Road NE, Atlanta, GA 30322 USA

**Keywords:** Drug delivery, Tumour immunology, Tumour immunology

## Abstract

Due to their autosynchronous roles in shaping the anti-tumor immune response, complex immune regulatory networks acting both locally within the tumor microenvironment as well as in its draining lymph nodes play critical roles in the cancer immunotherapy response. We describe herein a thermosensitive co-polymer hydrogel system formed from biocompatible polymers gelatin and Pluronic^®^ F127 that are widely used in humans to enable the sustained release of a nitric oxide donor and antibody blocking immune checkpoint cytotoxic T-lymphocyte-associated protein-4 for efficient and durable anti-tumor immunotherapy. By virtue of its unique gel formation and degradation properties that sustain drug retention at the tumor tissue site for triggered release by the tumor microenvironment and formation of in situ micelles optimum in size for lymphatic uptake, this rationally designed thermosensitive hydrogel facilitates modulation of two orthogonal immune signaling networks relevant to the regulation of the anti-tumor immune response to improve local and abscopal effects of cancer immunotherapy.

## Abstract (web summary)

Nitric oxide exerts a multitude of physiological functions and has also been exploited for anticancer therapies. Here the authors report the design of a micelle-releasing thermosensitive hydrogel system for the concomitant locoregional and lymphatic delivery of a nitric oxide donor and an anti-CTLA4 antibody, showing anti-tumor immune responses in preclinical cancer models.

## Introduction

The advent of the cancer immunotherapy era brought by approval of therapy antagonizing the immune checkpoint cytotoxic T-lymphocyte-associated protein-4 (CTLA-4) revolutionized the outlook on cancer therapy^1–7^. Existing immune checkpoint blockade (ICB) therapies are based on function-blocking antibodies that thwart the suppressive effects of these and other signaling pathways to unleash antitumor functions of a patient’s immune system. Despite efficacious and durable responses achieved in a subset of patients, low response rates in most cancer types and systemic immune-related adverse events limit ICB’s impact on patient outcomes^3,4^. Use in combination with other immune-boosting therapies, including but not limited to radiation, chemotherapy, chimeric antigen receptor T cells, cytokines, vaccination, and molecular adjuvants, as well as development of controlled and targeted delivery systems offer the potential to address these limitations and broaden ICB’s benefits across and within patient pools^1–8^. Controlled delivery and release technologies furthermore offer unique advantages to this end by unlocking the synergies of combination therapeutics within their target tissue site(s) while simultaneously minimizing the need for repeated administration, thereby reducing treatment costs and patient compliance barriers^2^.

Nitric oxide (NO) is an endogenous gaseous molecule that governs a myriad of physiological functions^9–17^ and whose levels in tumors are closely related to the therapeutic effects of conventional cancer therapies^13–15,17,18^. In particular, functions of NO in apoptosis, drug efflux, and vascular vasodilation/normalization have recently been highlighted for their beneficial effects in anticancer therapies, motivating the rapid progress of NO delivery systems for therapeutic applications^9–17^. These investigations have largely overlooked the complex regulatory effects of NO on intra- and intercellular immune networks, however, including those relevant to cancer immunotherapy. Indeed, treatment with high dose NO that results in tumor cell death failed to augment antitumor immunity despite the expansion and activation of dendritic cells (DCs) and macrophages^14,15^. Low dose NO, on the other hand, resulted in a modest in vivo expansion of T cells, a response not associated with improved antitumor therapeutic effects^14^. Given the complex immune regulatory and stimulatory effects of NO^14,15^, controlled delivery and release systems offer unique opportunities to control the overall immunomodulatory functions of exogenously delivered NO to potentiate cancer immunotherapy^17^.

In this work, we demonstrate that immune regulatory networks of NO and CTLA-4 can be therapeutically ameliorated for cancer immunotherapy by a thermosensitive biomaterial-based hydrogel system for controlled locoregional delivery of both a NO-donor and monoclonal antibody (mAb) antagonizing CTLA-4 (aCTLA-4). Treatment with *S*-nitrosoglutathione (GSNO), the most widely explored NO-donor for in vivo applications^19^, results in DC expansion and activation both locally and systemically, but its functions appear restrained by simultaneous expansion of various CTLA-4-expressing immune cells. Repeated treatment with the combination of GSNO and aCTLA-4 mAb leads to antitumor therapeutic effects that are systemic in a melanoma model that is resistant to either GSNO and aCTLA-4 administered systemically as monotherapies. A thermosensitive hydrogel synthesized via a simple chemical conjugation between clinically apposite Pluronic^®^ F127 and gelatin improves both the direct and abscopal antitumor effects of combination therapy via sustained release of GSNO and aCTLA-4 mAb loaded in situ micelles from the hydrogel delivered into the treated tumor. This sustained-release drug delivery system, comprised entirely of biocompatible polymers and therapeutic agents already approved for or in investigational human use, thus further potentiates the benefits afforded by sustained ICB by modulating orthogonal immune networks regulated by NO in tissues relevant to melanoma immunology, unleashing new therapeutic windows relevant to the treatment of advanced, unresectable disease to improve immunotherapy outcomes.

## Results

### Exogenous GSNO promotes tumor growth and enriches CTLA-4-expressing immune cell milieus within lymphoid tissues

**Fig. 1:**
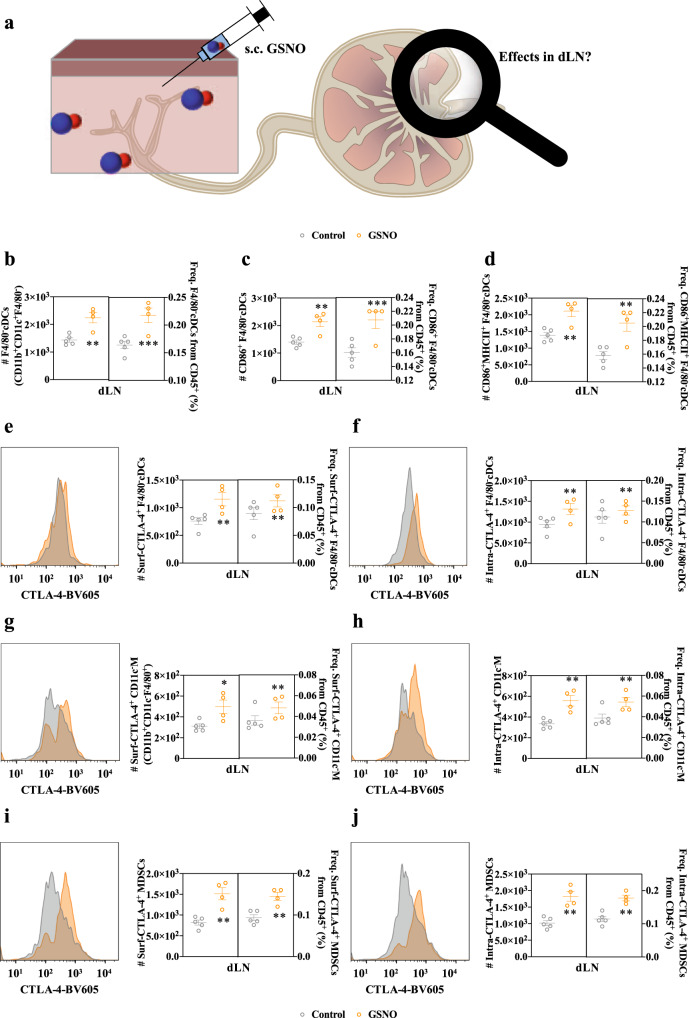
Subcutaneously injected GSNO influences on antigen presenting cell levels and expression of CTLA-4 in LNs draining the tissue site of injection. Immune phenotyping of dLN leukocyte populations 1 day after treatment of GSNO (570 μg kg^−1^) in 30 μL saline. Gating strategy can be found in Supplementary Fig. 1. **a** Schematic illustration to investigate the effects of subcutaneously injected GSNO on immune cells in dLN. **b**–**d** Number and frequency of (**b**) CD45^+^CD11b^+^CD11c^+^F4/80^−^ cDCs (F4/80^−^cDCs) (left *p* = 0.0321 and right *p* = 0.0046), (**c**) CD86^+^ activated F4/80^−^cDCs (CD86^+^ F4/80^−^cDCs) (left *p* = 0.0485 and right *p* = 0.0087), and (**d**) CD86^+^ and MHCII^+^ activated F4/80^−^cDCs (CD86^+^MHCII^+^ F4/80^−^cDCs) (left *p* = 0.0466 and right *p* = 0.0120) within dLNs. **e**–**j** Representative histograms (left) and number and frequency of cell subpopulation (right) of (**e**, **g**, **i**) surface and (**f**, **h**, **j**) intracellularly expressed CTLA-4 by various dLN leukocyte populations. **e**, **f** F4/80^−^cDCs (**e** left *p* = 0.0277, **e** right *p* = 0.0480, **f** left *p* = 0.0298, and **f** right *p* = 0.0321). **g**, **h** CD11b^+^CD11c^-^F4/80^+^ macrophages (CD11c^-^M) (**g** left *p* = 0.0772, **g** right *p* = 0.0377, **h** left *p* = 0.0396, and **h** right *p* = 0.0123). **i**, **j** CD45^+^CD11b^+^GR-1^+^ (MDSCs) (**i** left *p* = 0.0353, **i** right *p* = 0.0285, **j** left *p* = 0.0375, and **j** right *p* = 0.0192). Number and frequency data are presented as individual biological replicates and mean ± SEM. **b**–**j** *n* = 5 for control and *n* = 4 for GSNO. *****p < 0.0001, ****p < 0.001, ***p < 0.01, **p < 0.05, and *p < 0.1 by two-tailed Student *t*-test. Source data are available in a Source Data file.

Given NO’s reported effects on tumor immunity-relevant regulatory networks^9,15^, responses by immune cells located within lymph nodes (LNs) draining the site of subcutaneous (s.c.) injection (dLN) to administered GSNO were profiled (Fig. 1a), as well as cells within non-draining LNs (ndLNs) and spleens. Many changes within cells located within draining lymphoid tissue microenvironments were found to result from GSNO treatment (Fig. 1b–j and Supplementary Figs. 1–19). CD45^+^CD11b^+^CD11c^+^F4/80^−^ DCs [F4/80^−^ conventional DCs (F4/80^−^cDCs)] were expanded and activated in dLN and expanded in spleen (Fig. 1b–d and Supplementary Fig. 3, 4), while other subsets of CD11b, CD11c and/or F4/80 expressing immune cells were maintained at similar levels in dLN, spleen, and ndLN (Supplementary Figs. 3–6). CD11b^+^CD11c^+^ DCs (cDC type 2, cDC2) are known to preferentially direct CD4^+^ over CD8^+^ T cells, though their precise functions are largely unknown due to their heterogenicity^20–22^. Nevertheless, frequencies of total (CD45^+^CD3^+^) and CD4 (CD45^+^CD3^+^CD4^+^) versus CD8 (CD45^+^CD3^+^CD4^−^) T cells were significantly decreased within LN draining the injection site (Supplementary Fig. 7), spleens and ndLNs (Supplementary Figs. 8–9). In addition, the overall levels of immunosuppressive cells, including regulatory T cells (T_reg_s, CD45^+^CD3^+^CD4^+^Foxp3^+^) and myeloid-derived suppressor cells (MDSCs, CD45^+^CD11b^+^Gr1^+^), in lymphoid tissues remained unchanged by GSNO treatment (Supplementary Figs. 10–11). These results imply that rather than expanding populations of immune suppressive cells (e.g., T_reg_s and MDSCs) within lymphoid tissues, GSNO may influence adaptive immune signaling through its enhancement of these cells’ regulatory functions.

CTLA-4, which is expressed widely on T cells as well as various other cells, including cancer cells^23,24^, DCs^25^, and MDSCs^26^, is an immune checkpoint whose major role is attributed to the modulation of T cell priming, differentiation, and function^1,3,8,24–26^. When expressed by cancer cells, CTLA-4 has also been shown to suppress the maturation and functions of DCs^23,24^. Nevertheless, the effects of NO-delivery on CTLA-4 expression have never been investigated, although NO donors were reported to control the activity of AP-1 (transcription factor as well as clathrin adaptor protein)^27^ that governs both the metabolism and expression of CTLA-4^28^. Accordingly, extracellular and intracellular expression of CTLA-4 by various immune cells was also profiled (Fig. 1e–j and Supplementary Figs. 12–19). Interestingly, the populations of extra- and intracellular CTLA-4 expressing F4/80^−^cDCs in dLN (Fig. 1e, f), and CD11b^+^CD11c^-^F4/80^+^ (CD11c^-^M) in dLN (Fig. 1g, h) were significantly expanded, while the CTLA-4 expression on/in other subsets of CD11b, CD11c and/or F4/80 expressing immune cells were negligibly changed in dLN, spleen, and ndLN (Supplementary Figs. 12–17). In addition, CTLA-4 expressing MDSCs (Fig. 1i, j) were significantly expanded in dLN, while there were no changes in spleen and ndLN (Supplementary Fig. 18). However, the expression of CTLA-4 by T_reg_s was reduced in dLN, spleen, and ndLN (Supplementary Fig. 19).

**Fig. 2:**
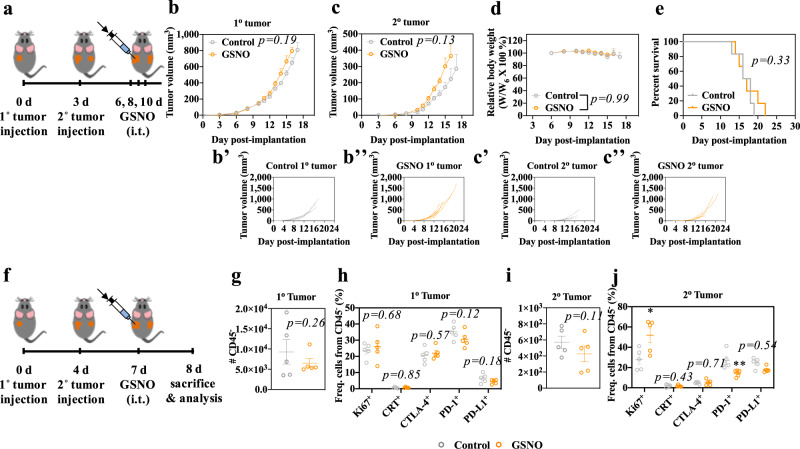
Intratumorally administered GSNO effects on tumor growth. **a** Tumor model and treatment schedule. 1^o^ and 2^o^ tumors were formed in C57Bl/6 mice by inoculation of 10^5^ B16F10-OVA cells in 30 μL saline on day 0 and day 3, respectively. GSNO (570 μg kg^−1^) in 30 μL saline was administered on day 6, 8, and 10. **b**, **c** Average and (B', B'', C', C'') individual volumes of (**b**) 1^o^ (directly injected) and (**c**) 2^o^ tumors (uninjected). **d** Relative body weight changes post treatment. **e** Kaplan–Meier survival curves. **f** Tumor model and treatment schedule. 1^o^ and 2^o^ tumors were formed by inoculation of 10^5^ B16F10-OVA cells in 30 μL saline on day 0 and day 4, respectively. GSNO (570 μg kg^−1^) was administered i.t. on day 7. Gating strategy can be found in Supplementary Fig. 22. **g**–**j** Number or frequency of each population of the indicated parent gate in the (**g**, **h**) 1^o^ or (**i**, **j**) 2^o^ tumor. **g**, **i** CD45^−^; **h**, **j** Ki-67^+^ (**j** *p* = 0.0677), CRT^+^, CTLA-4^+^, PD-1^+^ (**j** *p* = 0.0144), and PD-L1^+^ of CD45^−^. Data are presented as individual biological replicates and mean ± SEM. **b**–**e** *n* = 6. **g**–**j** *n* = 5. *****p < 0.0001, ****p < 0.001, ***p < 0.01, **p < 0.05, and *p < 0.1. **b, c** ANOVA using linear mixed-effects model. **d** Two-way ANOVA using Tukey post-hoc statistical hypothesis. **e** Log-rank using Mantel–Cox statistical hypothesis. **g**–**j** Two-tailed Student *t*-test. Source data are available in a Source Data file.

The therapeutic effects of GSNO on the growth of B16F10-OVA melanoma-inoculated C57BL/6 mice were evaluated. Repeated (3x) intravenous (i.v.) administration of GSNO at 600 μg kg^−1^ resulted in prolonged animal survival (Supplementary Fig. 20). Contrastingly, intratumoral (i.t.) administration of 570 μg kg^−1^ GSNO (Fig. 2) slightly accelerated tumor growth, effects seen both in the treated (primary, 1^o^) tumor as well as in an untreated (secondary, 2^o^) tumor implanted in the contralateral dorsal skin (Fig. 2b, c). Tumor growth effects were not associated with changes in animal weight or survival (Fig. 2d, e), nor was GSNO treatment found to induce in any direct cytotoxic or cytostatic effects on B16F10-OVA cells in vitro (Supplementary Fig. 21) or proliferation in vivo, as suggested by no change in frequencies of Ki-67^+^ CD45^−^ cells (Fig. 2h). These results imply that i.t. administration of GSNO may have protumoral effects that are immune-mediated, including but not limited to tumor cell immunogenicity or by expanding tolerogenic immune cells that foster immunosuppressive tumor microenvironments. However, except for expression of PD-1 by CD45^−^ cells in the contralateral (untreated) tumor, GSNO administered i.t. appeared to exert negligible effects on expression of tumor immunogenicity markers including calreticulin (CRT), CTLA-4, PD-1, and PD-L1 (Fig. 2f–j and Supplementary Fig. 22). These observations suggest that the protumoral effects of GSNO may be associated with the CTLA-4 mediated hindrance of antitumor immunity, rather than direct effects of NO on tumor cell immunogenicity or proliferation, a hypothesis consistent with GSNO’s expansion of CTLA-4 expressing DCs, macrophages, and MDSCs and within LNs draining the locoregional site of injection.

### Immunotherapeutic effects of GSNO and aCTLA-4 are enhanced in combination

**Fig. 3:**
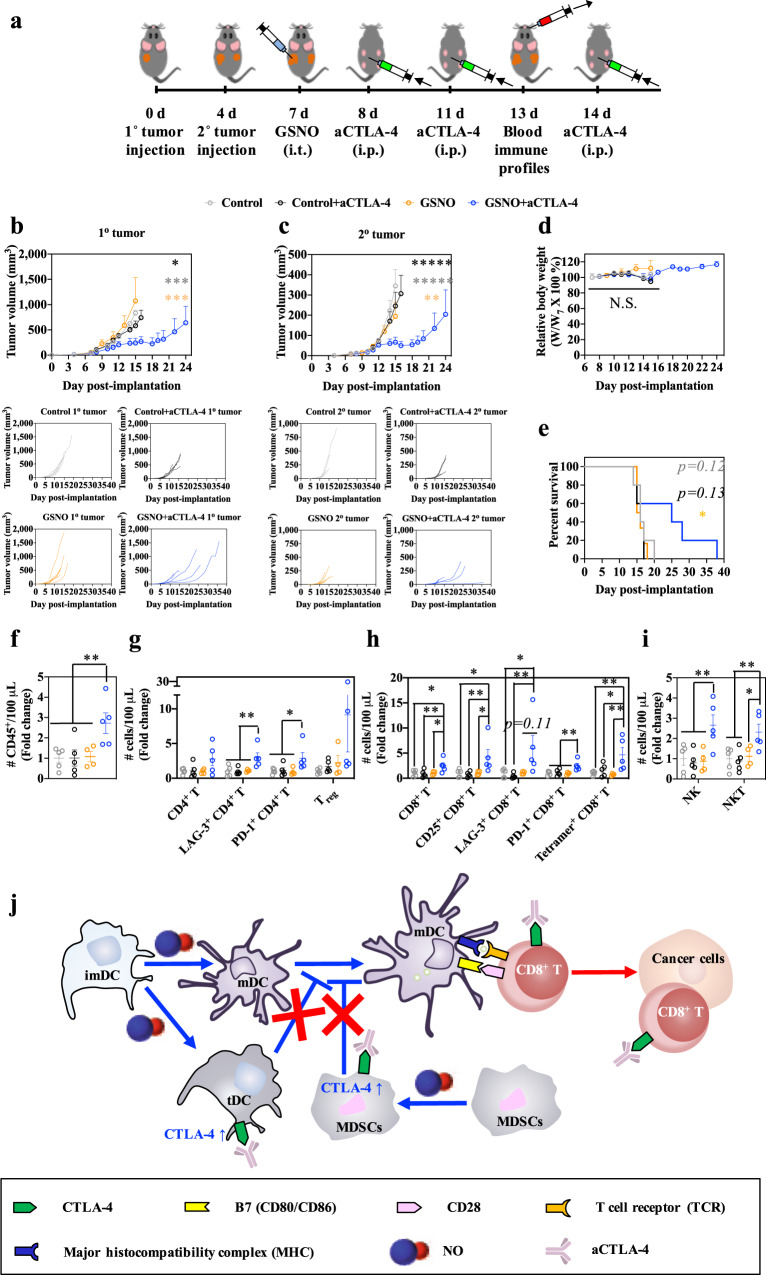
Direct and abscopal therapeutic effects and immunomodulation by GSNO and aCTLA-4 combination therapy. **a** Tumor model and treatment schedule. 1^o^ and 2^o^ tumors were formed in C57Bl/6 mice by inoculation of 10^5^ B16F10-OVA cells in 30 μL saline on day 0 and day 4, respectively. GSNO (480 μg kg^−1^) in 30 μL saline was intratumorally treated on day 7, and aCTLA-4 (100 μg mouse^−1^) in 30 μL saline was intraperitoneally administered on day 8, 11, and 14. Blood was harvested from the facial vein on day 13 for the profiling of blood immune cells. **b** Average and individual volumes of 1^o^ (directly injected) tumors. **c** Average and individual volumes of 2^o^ (uninjected) tumors. **d** Relative body weight changes post treatment. **e** Kaplan–Meier survival curves. **f**–**i** Relative blood abundance of **f** CD45^+^, **g** CD45^+^CD3^+^CD4^+^ T (CD4^+^ T), LAG-3^+^ CD4^+^ T, PD-1^+^ CD4^+^ T, and CD45^+^CD3^+^CD4^+^CD25^+^Foxp3^+^ (T_reg_), **h** CD45^+^CD3^+^CD8^+^ T (CD8^+^ T), CD25^+^ CD8^+^ T, LAG-3^+^ CD8^+^ T, PD-1^+^ CD8^+^ T, and tetramer^+^ CD8^+^ T, and **i** CD45^+^CD3^-^NK1.1^+^ (NK) and CD45^+^CD3^+^NK1.1^+^ (NKT). Data are presented as individual biological replicates and mean ± SEM. **b**–**e** *n* = 5 for Control, Control+aCTLA-4, and GSNO + aCTLA-4, and *n* = 6 for GSNO. **f**–**i** *n* = 5 for Control, Control+aCTLA-4, and GSNO + aCTLA-4, and *n* = 4 for GSNO. *****p < 0.0001, ****p < 0.001, ***p < 0.01, **p < 0.05, and *p < 0.1. Exact *p*-values for **b**, **c** and **e**–**i** are reported in Supplementary Table 1 and 2. **b**, **c** ANOVA using linear mixed-effects model. **d** Two-way ANOVA using Tukey post-hoc statistical hypothesis. **e** Log-rank using Mantel–Cox statistical hypothesis by comparing the GSNO + aCTLA-4 with control groups. **f**–**i** One-way ANOVA using Tukey post-hoc statistical hypothesis. **j** Proposed actions of combinational use of GSNO and aCTLA-4 on immune response. Blue arrows indicate the mechanism associated with GSNO. Red arrows and crosses represent mechanisms associated with aCTLA-4. Source data are available in a Source Data file.

The combination of intraperitoneally (i.p.) administered mAb antagonizing CTLA-4 signaling (aCTLA-4 mAb) with i.t. GSNO treatment was evaluated for its potential to unleash the functions of activated and mature DCs that appear to be restrained by CTLA-4 expressing tolerogenic DCs, macrophages, and MDSCs induced by GSNO treatment (Figs. 1, 2) using a dual B16F10-OVA mouse tumor model to reveal direct as well as abscopal therapeutic effects (Fig. 3a). The combination therapy, but not GSNO or aCTLA-4 when used as monotherapies, led to a significant slowing of the treated 1^o^ tumor’s growth, despite having no cytotoxic effects on B16F10-OVA cells in vitro (Fig. 3b, Supplementary Table 1, and Supplementary Figs. 21 and 23), suggestive of the therapeutic benefit not being associated with direct drug effects on the tumor. Treatment with the combination therapy furthermore resulted in substantial diminution in the growth of a contralateral tumor with no change in animal weight, indicating a strong abscopal effect, and animal survival was improved compared to treatment with GSNO alone (Fig. 3c–e and Supplementary Table 1 and 2). Consistent with these observed therapeutic benefits, the combination therapy was associated with an expansion of CD4^+^ T, CD8^+^ T, CD3^-^NK1.1^+^ (NK), and CD3^+^NK1.1^+^ (NKT cells, NKT) cells in the blood day 13 post tumor implantation (Fig. 3a, f–i and Supplementary Table 2). Suggestive of robust priming of tumor antigen-specific T cells underlying these improvements in tumor control enabled by GSNO and aCTLA-4 when used in combination, the populations of CD4^+^ and CD8^+^ T cells that express activation markers CD25^+^ and LAG-3^+^, as well as antigen-experience marker PD-1^+^, were increased in the blood, as were tetramer-positive, tumor antigen-specific CD8^+^ T cells (Fig. 3g, h)^29,30^. CTLA-4 antagonism with GSNO treatment thus appears to suppress the regulatory functions of CTLA-4 on immunosuppressive immune cells induced by GSNO, resulting in expansion of NK^31^ and NKT^31^ cells and improved priming of T cells (Fig. 3j).

### F127-*g*-Gelatin thermosensitive hydrogel facilitates the sustained and targeted delivery of GSNO and aCTLA-4

The potential for sustained release technology to benefit immunomodulatory and/or immunotherapy applications is now established^32–37^. In addition, considering the potential side effects of systemic NO delivery in blood pressure^38^, local NO delivery systems have also attracted significant attention in biomedical applications^10^. To this end, injectable thermosensitive hydrogels offer numerous advantages, including their simple, simultaneous loading of diverse drug types, facile administration without surgery, and prolonged drug release from the polymer matrix^37,39^.

Lower critical solution temperature (LCST) polymer F127 is widely utilized because it is cheap, biocompatible, and renal clearable. Despite its FDA approval, however, its practical hydrogel application is limited due to short residence in aqueous and physiological conditions^39,40^. Accordingly, we hypothesized that the grafting F127 onto the also FDA-approved polymer gelatin, which is likewise biocompatible and exhibits low antigenicity, is compositionally diverse to provide sufficient functional groups amenable for easy chemical modifications, and is biodegradable and responsive to matrix metalloproteinases (MMPs) that are overexpressed by melanoma as well as various metastatic tumors,^41–43^ would yield a biocompatible and biodegradable thermosensitive hydrogel that would facilitate the sustained delivery and therapeutic effects of aCTLA-4 mAb and GSNO in vivo. Such an approach would reduce the number of injections needed to elicit the therapeutic effects of GSNO and aCTLA-4 when used in combination for melanoma immunotherapy.

**Fig. 4:**
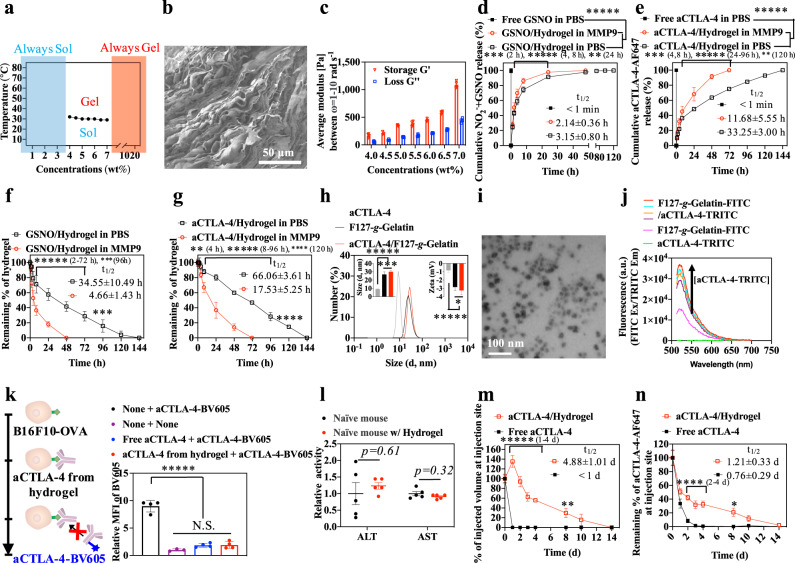
In vitro and in vivo physicochemical properties, residence stability, and drug release behavior of thermosensitive hydrogels formed from F127-*g*-Gelatin. **a** Concentration-dependent sol-gel transition properties of F127-*g*-Gelatin. **b** Representative SEM image of F127-*g*-Gelatin hydrogel (*n* = 3). **c** Concentration-dependent storage (G') and loss (G'') modulus of F127-*g*-Gelatin at 37 °C (*n* = 3 for 4.5 wt.%, *n* = 4 for 5.5, 6.0, 6.5, and 7.0 wt.%, and *n* = 5 for 4.0 and 5.0 wt.%.). **d**, **e** Cumulative release of (**d**) NO_2_^−^ + GSNO (*n* = 4) and (**e**) Alexa Fluor^TM^ 647 labeled aCTLA-4 (*n* = 3) from 4.5 wt% F127-*g*-Gelatin into PBS with or without MMP9. **f**, **g** In vitro residence stability of (**f**) GSNO (*n* = 4) and (**g**) Alexa Fluor^TM^ 647 labeled aCTLA-4 (*n* = 3) containing 4.5 wt% F127-*g*-Gelatin in PBS with or without MMP9. **h** DLS size distribution of aCTLA-4 and supernatants released from F127-*g*-Gelatin hydrogel (F127-*g*-Gelatin) or F127-g-Gelatin hydrogel containing aCTLA-4 (aCTLA-4/F127-*g*-Gelatin) (*n* = 12). The left and right insets represent the average size (*n* = 12) and zeta potentials (*n* = 6 for aCTLA-4, and *n* = 12 for the other) of materials, respectively. **i** Representative TEM image of F127-*g*-Gelatin micelles in situ released from F127-*g*-Gelatin thermosensitive hydrogel (*n* = 3). **j** FRET analysis with aCTLA-4-TRITC and F127-*g*-Gelatin-FITC at FITC excitation and TRITC emission (*n* = 4). **k** Competitive assay to verify the activity of aCTLA-4 released from F127-*g*-Gelatin hydrogel (*n* = 3 for None+None, and *n* = 4 for the other). **l** ALT/AST activities of blood taken from mice 2 d after subcutaneous administration of 4.5 wt.% F127-*g*-Gelatin hydrogel (*n* = 5). **m** In vivo residence stability of 4.5 wt.% F127-*g*-Gelatin hydrogel quantified by time-resolved volume of hydrogel remaining at the injection site (*n* = 4). **n** In vivo quantification of aCTLA-4-AF647 remaining at the injection site by using IVIS^®^ resulting from formulation in the 4.5 wt.% F127-*g*-Gelatin hydrogel (*n* = 4). Data are presented as mean ± SD for **c**–**h** and **j**, **k**, and mean ± SEM for **l**–**n**. *****p < 0.0001, ****p < 0.001, ***p < 0.01, **p < 0.05, and *p < 0.1. Exact *p*-values for **d**–**h**, **k**, **m**, and **n** are reported in the source file. **d**–**g**, **m**, **n** Two-way ANOVA using Tukey post-hoc statistical hypothesis. **h**, **k** One-way ANOVA using Tukey post-hoc statistical hypothesis. **l** Two-tailed Student *t*-test. Source data are available in a Source Data file.

F127-grafted gelatin (F127-*g*-Gelatin) was synthesized by conjugation of 4-nitrophenyl chloroformate-activated hydroxyl groups of F127 to gelatin amine groups (Supplementary Figs. 24–26). The grafted F127-*g*-Gelatin polymer formed thermosensitive hydrogels at very low concentrations (4.0–7.0 wt.%), a surprising result given gelatin’s upper critical solution temperature (UCST) behavior (Fig. 4a, Supplementary Figs. 27,28 and Supplementary Table 3,4). This enhanced thermosensitive behavior was not observed in the mixture of F127 and gelatin (Supplementary Fig. 28c, and Supplementary Table 4), in contrast to previously reported F127-Gelatin copolymers that show sol-gel transition behavior (>10–15 wt.%) similar to that of bare F127 (> ~15 wt.%)^44,45^. F127-*g*-Gelatin showed a reduced peak for crystalline structure of triple-helix (2*θ* = 8.4)^46^ and an increased peak for amorphous phase (2*θ* = 21.1)^47^ of gelatin in X-ray diffraction (XRD) compared to gelatin alone (Supplementary Fig. 29), negating potential contributions of coil-to-helix conversion to the observed thermosensitive gelation behavior^45^. In addition, the crystalline peak for F127 (2*θ* = 19.2 and 23.4)^46^ was reduced in F127-*g*-Gelatin, compared to F127 (Supplementary Fig. 29). Furthermore, F127-*g*-Gelatin exhibited no additional crystalline peaks in differential scanning calorimeter (DSC), compared to gelatin and F127 (Supplementary Fig. 30,31). On the other hand, F127-*g*-Gelatin showed significantly decreased critical micellar concentration (CMC) with the increase of temperature-dependency in CMC compared to F127 and the mixture of F127 and gelatin (Supplementary Fig. 32, and Supplementary Table 5). These results indicate that enhanced amorphous hydrophobic interactions contribute to the improved thermosensitivity of F127-*g*-Gelatin. The resultant F127-*g*-Gelatin hydrogels exhibited sheet-like microstructures (Fig. 4b) capable of solvent entrapment, which might enhance the hydrogel’s swelling property to contribute to the diffusion-mediated release of drugs. In addition, F127-*g*-Gelatin hydrogel showed the concentration-dependent rheology at 37 °C (Fig. 4c, and Supplementary Fig. 33).

The potential for the resultant F127-*g*-Gelatin hydrogels for sustained drug release was next evaluated. Total levels of nitrite (NO_2_^−^) and GSNO or Alexa Fluor^TM^ 647-labeled aCTLA-4 (aCTLA-4-AF647) (Supplementary Fig. 34) were released in a sustained manner from GSNO- or aCTLA-4-loaded 4.5 wt.% F127-*g*-Gelatin hydrogels (Fig. 4d, e), a process accelerated by enzymatic degradation with MMP9 (Fig. 4d, e) that is commonly overexpressed in melanomas^41–43^. Interestingly, F127-*g*-Gelatin hydrogels containing aCTLA-4-AF647 mAb exhibited prolonged residence times as well as release half-lives in vitro compared to F127-*g*-Gelatin hydrogels containing GSNO (Fig. 4d–g), implicating the association of the loaded aCTLA-4 mAb in the formation of F127-*g*-Gelatin hydrogels. Indeed, aCTLA-4 mAb (*d* = 9.3 ± 0.6 nm) was not detected separately in the supernatants released from aCTLA-4 mAb loaded F127-*g*-Gelatin hydrogels in dynamic light scattering (Fig. 4h). In particular, the hydrogel released spherical micelles (Fig. 4i). The size of the in situ-formed micelles released from aCTLA-4 mAb loaded F127-*g*-Gelatin hydrogels (*d* = 30.0 ± 1.8 nm) was significantly larger than those of bare F127-*g*-Gelatin hydrogels (*d* = 26.8 ± 3.1 nm) (Fig. 4h), implying the loading of aCTLA-4 on the F127-*g*-Gelatin micelles. The in situ release of F127-g-Gelatin micelles would be attributed to Pluronic^®^ F127 components which self-assemble into the micelles via the dehydration of hydrophobic blocks with the increase in the entropy of the system at above critical micelle concentration (CMC)^48^. Nevertheless, the size of aCTLA-4-loaded F127-*g*-Gelatin micelles was in a size range appropriate for efficient lymphatic uptake (10–100 nm)^49,50^, which raised an expectation of efficient aCTLA-4 functions in both the dLN as well as tumor microenvironment site of injection^34^.

The interactions of aCTLA-4 mAb with F127-*g*-Gelatin in situ-formed micelles were further verified with the appearance of additional CMC (CMC_2_) in F127-*g*-Gelatin solutions containing aCTLA-4 (Supplementary Fig. 35 and Supplementary Table 6) and the fluorescence resonance energy transfer (Fig. 4j) between TRITC-labeled aCTLA-4 (aCTLA-4-TRITC) (Supplementary Fig. 36a) and FITC-labeled F127-*g*-Gelatin (F127-*g*-Gelatin-FITC) (Supplementary Fig. 36b) at FITC excitation and TRITC emission. Considering that F127 has an ability to bind human serum albumin via hydrogen bonding and hydrophobic interactions^51^ and aCTLA-4 mAb in F127 micelles was not detected solely in dynamic light scattering measurements (Supplementary Fig. 37), the F127 blocks in F127-*g*-Gelatin may contribute to the formation of aCTLA-4 mAb loaded in situ F127-*g*-Gelatin micelles. In addition to the larger molecular size of aCTLA-4 than GSNO, this affinity of aCTLA-4 with F127-*g*-Gelatin would also allow the dependency of aCTLA-4 release on the hydrogel degradation (Fig. 4e, g). On the other hand, the weaker dependence of GSNO release on the degradation of F127-*g*-Gelatin matrix may be attributed to its higher rate of diffusion through the F127-*g*-Gelatin hydrogel (Fig. 4d, f). When supernatants of in vitro pre-incubated aCTLA-4-loaded F127-*g*-Gelatin hydrogels were pretreated onto B16F10-OVA cells, staining with aCTLA-4-BV605 was blocked to similar extents as the treatment that seen with free aCTLA-4 (Fig. 4k). These results demonstrate that despite the interactions between aCTLA-4 mAb and F127-*g*-Gelatin polymer, the activity of aCTLA-4 mAb is not diminished by incorporation into the F127-*g*-Gelatin in situ-formed micelles.

**Fig. 5:**
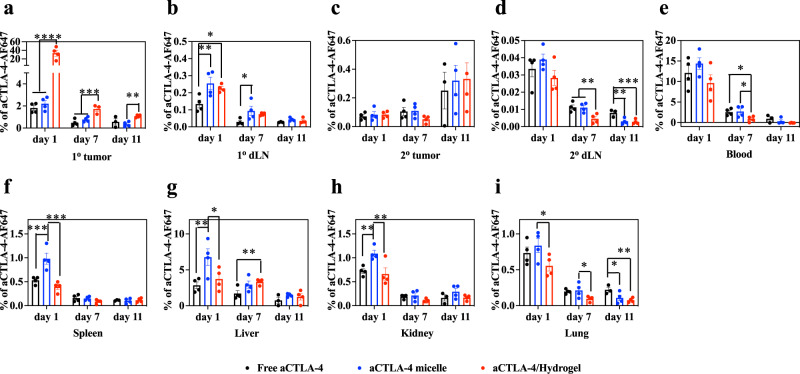
In vivo biodistribution of aCTLA-4 released from F127-*g*-Gelatin thermosensitive hydrogels. **a**–**i** Biodistribution of free aCTLA-4-AF647, aCTLA-4-AF647 with 0.45 wt.% F127-g-Gelatin micelles (aCTLA-4 micelle), and aCTLA-4-AF647 with 4.5 wt.% F127-*g*-Gelatin hydrogel (aCTLA-4 dose equivalent to 162 µg mouse^−1^) administered into the 1^o^ tumor of C57Bl/6 mice bearing 1^o^ and 2^o^ tumors inoculated with B16F10-OVA 10^5^ cells in 30 μL saline on day 0 and day 4, respectively. **a** 1^o^ (directly injected) tumor; **b** LN draining the 1^o^ tumor (1^o^ dLN); **c** 2^o^ (uninjected) tumor; **d** LN draining the 2^o^ tumor (2^o^ dLN); **e** blood; **f** spleen; **g** liver; **h** kidney; **i** lung. Data are presented as mean ± SEM. *n* = 4 except (**a-i**) Free aCTLA-4 groups on day 11 (*n* = 3) and (**a**) aCTLA-4/Hydrogel on day 7 (*n* = 3). *****p < 0.0001, ****p < 0.001, ***p < 0.01, **p < 0.05, and *p < 0.1 with one-way ANOVA using Tukey post-hoc statistical hypothesis. Exact *p*-values for **a–i** are reported in Supplementary Table 7. Source data are available in a Source Data file.

As an LCST polymer, F127-*g*-Gelatin solution (4–7 wt.%) existed in a sticky liquid state inside the syringe and underwent gelation after administration. In line with negligible cytotoxicity in vitro (Supplementary Fig. 38), the F127-*g*-Gelatin hydrogel administered i.d. in vivo had no effect on body weight (Supplementary Fig. 39) nor systemic liver toxicity, as measured by serum alanine aminotransferase (ALT) and aspartate aminotransferase (AST) levels (Fig. 4l). When the injected tissue volume and fluorescent signal of aCTLA-4-AF647 mAb loaded F127-*g*-Gelatin hydrogels were measured at the site of mouse dorsal skin injection, F127-*g*-Gelatin hydrogels were found to impart significantly longer residence times and sustained release of aCTLA-4 mAb in vivo compared to bolus delivery of free mAb (Fig. 4m, n and Supplementary Fig. 40). When administered i.t., aCTLA-4-AF647 mAb was retained at the injected tumor site to greater extents in its hydrogel form (aCTLA-4/Hydrogel) for as long as 11 d compared to free aCTLA-4-AF647 and aCTLA-4-AF647 loaded micelles formed from F127-*g*-Gelatin (Fig. 5a and Supplementary Table 7). Administration of F127-*g*-Gelatin micelles resulted in accumulation of aCTLA-4-AF647 in LNs draining the tumor injection site to greater extents compared to bolus delivery but at levels similar to that seen for aCTLA-4-AF647 delivered via the administered F127-*g*-Gelatin hydrogels (Fig. 5b and Supplementary Table 7). Considering aCTLA-4 mAb loaded in situ micelles that exhibit a hydrodynamic size amenable for efficient lymphatic uptake (10–100 nm)^49,50^ are released from aCTLA-4 mAb containing F127-*g*-Gelatin hydrogels (Fig. 4i, j), efficient lymphatic delivery of aCTLA-4-AF647 with F127-*g*-Gelatin hydrogels can be attributed to the formation of in situ of aCTLA-4 loaded F127-*g*-Gelatin micelles. F127-*g*-Gelatin hydrogels thus facilitate not only the sustained i.t. accumulation of aCTLA-4 mAb, but also enable its efficient delivery into dLN. F127-*g*-Gelatin hydrogels also reduced the exposure of aCTLA-4-AF647 to other tissues (Fig. 5c–i and Supplementary Table 7) compared to aCTLA-4-AF647 in its free or F127-*g*-Gelatin micelle loaded form.

### Sustained GSNO + aCTLA-4 combination therapy using F127-*g*-gelatin augments antitumor immunotherapy

**Fig. 6:**
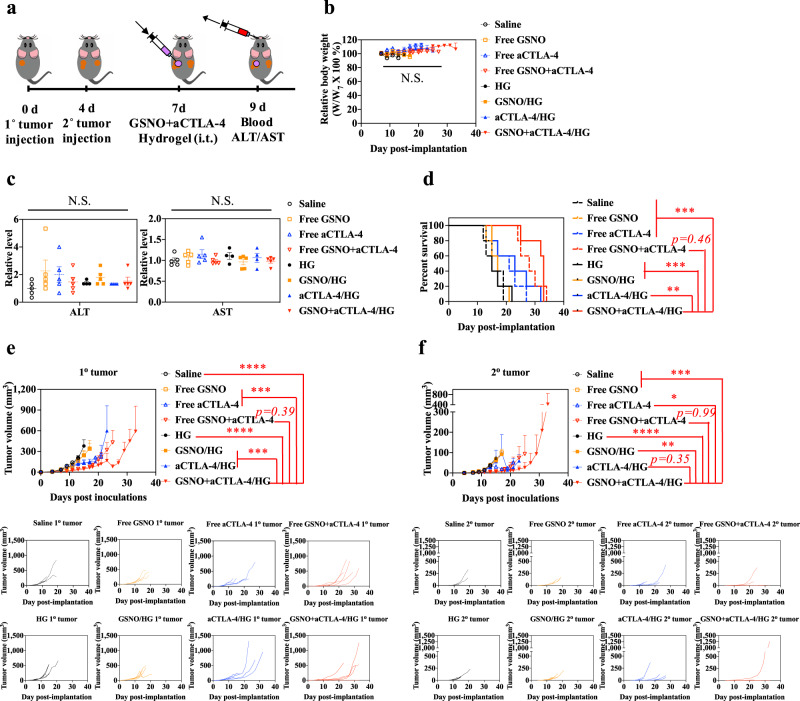
Direct and abscopal effects of GSNO and aCTLA-4 loaded F127-*g*-Gelatin hydrogel. **a** Tumor model and treatment schedule. 1^o^ and 2^o^ tumors were formed in C57Bl/6 mice by inoculation of 10^5^ B16F10-OVA cells in 30 μL saline on day 0 and day 4, respectively. GSNO (570 μg kg^−1^) and aCTLA-4 (50 μg mouse^−1^) were administered intratumorally on day 7 in a total volume of 30 μL in saline or 4.5 wt.% F127-*g*-Gelatin hydrogel. Blood was harvested from the facial vein on day 9 for assessment using the ALT/AST assay (*n* = 4 for HG and aCTLA-4/HG, and *n* = 5 for the other). **b** Relative body weight changes post treatment (*n* = 5). **c** ALT/AST activities of blood taken from mice 2 d after treatment (*n* = 5). **d** Kaplan–Meier survival curves (*n* = 5). **e** 1^o^ (directly injected) tumor size (*n* = 5). **f** 2^o^ (uninjected) tumor size (*n* = 5). Data are presented as individual biological replicates and mean ± SEM. *****p < 0.0001, ****p < 0.001, ***p < 0.01, **p < 0.05, and *p < 0.1. Exact *p*-values for **d**–**f** are reported in Supplementary Tables 8 and 9. **b** Two-way ANOVA using Tukey post-hoc statistical hypothesis. **c** One-way ANOVA using Tukey post-hoc statistical hypothesis. **d** Log-rank using Mantel–Cox statistical hypothesis. **e**, **f** ANOVA using linear mixed-effects model. Source data are available in a Source Data file.

The benefit afforded by the dual tissue (tumor and tumor-dLN) delivering F127-*g*-Gelatin hydrogel on combination GSNO and aCTLA-4 therapy was assessed in the dual B16F10-OVA tumor model (Fig. 6a). Although no significant changes in body weight or ALT/AST activity were observed in any group (Fig. 6b, c), animal survival in response to a single i.t. injection of GSNO and aCTLA-4 co-formulated within the F127-*g*-Gelatin hydrogel was prolonged (Fig. 6d and Supplementary Table 8). This survival benefit was associated with the antitumor effects (Fig. 6e, f and Supplementary Table 9). In line with our other results (Fig. 3), i.t. administration of GSNO in both free and hydrogel formulations showed negligible effects on tumor growth. aCTLA-4 formulated within F127-*g*-Gelatin hydrogel showed limited improvement of aCTLA-4’s therapeutic index with respect to 1^o^ tumor growth and animal survival compared to free aCTLA-4. However, unexpectedly, i.t. administration of free GSNO + aCTLA-4 exhibited similar therapeutic effects with that of free aCTLA-4. These results may suggest that locally high levels of antibody achieved by i.t. administration enable aCTLA-4 to better exert its therapeutic effects due to the aCTLA-4’s actions on the tumor microenvironment and dLN^34^. Nevertheless, combination of GSNO and aCTLA-4 co-formulated within F127-*g*-Gelatin hydrogel led to the decreased growth of the treated (1^o^) tumor with efficacies being slightly superior to that of the free drugs in combination. In addition, GSNO + aCTLA-4/HG also substantially diminished the contralateral untreated tumor (2^o^). In particular, one-time i.t. administration of GSNO + aCTLA-4/HG showed significantly higher antitumor effects than combined one-time i.t. administration of GSNO and three times i.p. administration of aCTLA-4 (Fig. 3, Supplementary Fig. 41, and Supplementary Table 10). These results suggest that prolonging the action of GSNO and aCTLA-4 mAb through i.t. administration of an F127-*g*-Gelatin hydrogel formulation through a single administration improves the combination therapy’s therapeutic index.

**Fig. 7:**
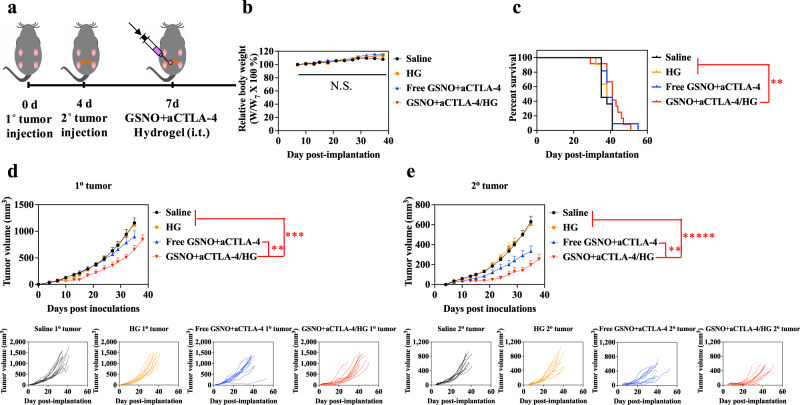
Antitumor effects of GSNO and aCTLA-4 loaded F127-*g*-Gelatin hydrogel on 4T1 tumor models. **a** Tumor model and treatment schedule. 1^o^ and 2^o^ tumors were formed in Balb/C mice by inoculation of 3 × 10^5^ 4T1 cells in 30 μL saline to left mammary fat pad on day 0 and at right mammary fat pad on day 4, respectively. GSNO (570 μg kg^−1^) and aCTLA-4 (50 μg mouse^−1^) were administered intratumorally on day 7 in a total volume of 30 μL in saline or 4.5 wt.% F127-*g*-Gelatin hydrogel. **b** Relative body weight changes post treatment. **c** Kaplan–Meier survival curves. **d** 1^o^ (directly injected) tumor size. **e** 2^o^ (uninjected) tumor size. Data are presented as individual biological replicates and mean ± SEM. *n* = 11 for saline, HG, and Free GSNO + aCTLA-4. *n* = 12 for GSNO + aCTLA-4/HG. *****p < 0.0001, ****p < 0.001, ***p < 0.01, **p < 0.05, and *p < 0.1. Exact *p*-values for **c**–**e** are reported in Supplementary Tables 11 and 12. **b** Two-way ANOVA using Tukey post-hoc statistical hypothesis. **c** Log-rank using Mantel–Cox statistical hypothesis. **d**, **e** ANOVA using linear mixed-effects model. Source data are available in a Source Data file.

Antitumor immunotherapeutic effects of GSNO + aCTLA-4/HG were also explored in the 4T1 model of mammary carcinoma (Balb/C mouse strain) using an aCTLA-4 (clone 9D9) that is of mouse origin (Fig. 7) in order to demonstrate the relevance of this immunotherapeutic synergy and drug delivery approach to another cancer and tissue type, a different mouse strain, and when employed using a therapeutic mAb of the same species as the host to replicate the human scenario. Despite the negligible survival benefit (Fig. 7c and Supplementary Table 11) and therapeutic effects on the 1^o^ tumor (FIg. 7d and Supplementary Table 12), i.t. administration of free GSNO + aCTLA-4 led to the slight antitumor effects on 2^o^ 4T1 tumors, compared to saline (Fig. 7d, e and Supplementary Table 12). The combination of GSNO and aCTLA-4 co-formulated within the F127-*g*-Gelatin hydrogel exhibited significantly stronger antitumor effects than saline, bare hydrogel, and free GSNO + aCTLA-4. These results not only indicate that the synergistic therapeutic effects of GSNO and aCTLA-4 in combination are maintained with a different antibody clone and species as well as in another mouse strain, but also imply the potential of locoregional sustained release platforms with combinational GSNO and aCTLA-4 therapy to cancers in different tissue sites and underlying biologies. Histological analysis of the mammary fat pad tumor injection site also revealed no effect of bare F127-*g*-Gelatin hydrogels compared to saline (Supplementary Fig. 42). Nor were any substantial changes in body weight measured for any treatment group (Fig. 7b). These results support the conclusion that the F127-*g*-Gelatin hydrogel exhibits no overt toxicity or inflammatory response and is generally biocompatible.

## Discussion

Locoregional therapies that boost the antitumor immune response as of one their mechanisms of action, including but not limited to radiation and oncolytic virus therapy, offer unique advantages for the treatment of not only unresectable disease but also advanced melanoma more broadly owing to their abscopal effects. These therapies’ successes are attributed to the elicitation of antitumor adaptive immune responses and, due to multiple immune regulatory mechanisms underlying tumor immune escape, are increasingly implemented with clinical success in combination with immunotherapies that modulate orthogonal immune regulatory pathways. Herein, we describe a drug delivery and release behavior of a thermosensitive hydrogel comprised of FDA-approved polymers to obviate the need for repeated administration, poor dosing control, and inability of current methods of administration to concentrate delivery and effects within target tissues. These polymers include Pluronic^®^ F127 and gelatin that have each been widely used with decades-long FDA-approval as a clinical biomaterial or food ingredient, respectively, because they are cheap, biocompatible, biodegradable, and/or renal clearable. The F127-*g*-Gelatin co-polymer can furthermore be prepared in a mass quantity via simple bioconjugation chemistry. When used to deliver FDA-approved/investigated drugs in combination, this formulation that upon degradation uniquely forms drug-laden micelles of a hydrodynamic size optimal for lymphatic uptake^49,50^ elicits more potent and durable therapeutic efficacy compared to bolus delivery. These favorable effects are furthermore associated with immune modulation within the tumor injection site as well as its dLNs to elicit robust abscopal effects to prolong animal survival. Co-formulated agents include: GSNO that is both endogenously ubiquitous and used therapeutically in a variety of preclinical cardiovascular and infectious disease applications; CTLA-4 antagonizing mAbs, which compared to other more recently developed ICB mAbs achieve overall lower rates of patient response clinically as a monotherapy^1–7^ but offer numerous attributes favorable to abscopal-eliciting locoregional therapies, including lower systemic adverse effects and broadening of the clonal repertoire of antitumor T cells^34^. As treatment with GSNO alone expands both activated DCs and CTLA-4-expressing immunosuppressive cells, the combinational use of antagonistic aCTLA-4 mAb with NO-donor GSNO results in synergistic and systemic anticancer effects in a melanoma tumor model resistant to either GSNO or aCTLA-4 administered systemically as monotherapies. This platform thus represents a technology highly amenable to clinical translation to enable NO’s immune modulatory functions to improve the therapeutic index of ICB therapy.

## Methods

### Ethical regulations

All research complied with the policies of the Georgia Institute of Technology.

### Synthesis of F127-*g*-Gelatin

20 g of Pluronic^®^ F127 (Sigma Aldrich, F127) in 50 mL dichloromethane (Sigma Aldrich, DCM) was added dropwise to 3.2 g of 4-nitrophenyl chloroformate (Sigma Aldrich, *p*-NPC) in 50 mL DCM with vigorous stirring. After overnight reaction, the resultant *p*-NPC activated F127 was precipitated in 2750 mL cold diethyl ether (Sigma Aldrich), followed by vacuum filtration. *p*-NPC activated F127 in 150 mL of 33.3% ethanol was added to 10 g gelatin type A (Sigma Aldrich, 300 g bloom) in 1 L deionized water containing 15 mL triethylamine (Sigma Aldrich) with vigorous stirring. After overnight reaction, the resulting F127-*g*-Gelatin co-polymer was dialyzed against deionized water (Spectrum Industries, MWCO 100 KDa) for 1.5 days, followed by freezing drying for 3 days.

### Synthesis of fluorescently labeled aCTLA-4 mAb or F127-*g*-Gelatin

5.4 mg of aCTLA-4 mAb (BioXCell, clone: 9H10) in 600 µL PBS was reacted with 35 µL of 10 mM Alexa Fluor^TM^ 647 NHS Ester (AF647-NHS) (Invitrogen^TM^) in DMSO at room temperature for 2 h. 1.8 mg of aCTLA-4 mAb in 200 µL PBS was reacted with 20 µL of 1 mg mL^−1^ TRITC (Thermo Scientific^TM^) in PBS at room temperature overnight. 8 mg of F127-*g*-Gelatin in 1 mL PBS was reacted with 160 µL of 1 mg mL^−1^ FITC (Thermo Scientific^TM^) in PBS at room temperature overnight. AF647-labeled aCTLA-4 mAb (aCTLA-4-AF647), TRITC-labeled aCTLA-4 mAb (aCTLA-4-TRITC), and FITC-labeled F127-*g*-Gelatin (F127-*g*-Gelatin-AF647) were purified using CL-6B Sepharose^®^ column (GE Healthcare) and an Amicon^®^ Ultra centrifugal filter (Milipore, MWCO 30 kDa) at 4000 g and 4 °C for 20 min.

### F127-*g*-Gelatin hydrogel characterization

The chemical composition of F127-*g*-Gelatin was analyzed with ^1^H nuclear magnetic resonance spectroscopy (^1^H NMR) with Bruker Advance 400 MHz FT-NMR using Topspin v3.0 software. NMR data were analyzed with MestreNova NMR v11. The vial tilting method was used to investigate the thermosensitivity of F127-*g*-Gelatin. Time-to-gelation is dependent on the volume of the solution, shape of the container for the solution, and the method used to increase the temperature. For example, 30 uL F127-*g*-Gelatin (4.5 wt.%) solution in e-tube at 37 °C water bath gelates within 15 s, while 1 mL F127-*g*-Gelatin (4.5 wt.%) solution in e-tube at 37 °C water bath gelates in 1 min. Differential scanning calorimetry (DSC, TA Instruments Q200) using Advantage v2.0 software and X-ray diffraction (XRD, Malvern PANalytical Empyrean) using Highscore v4.9 software were implemented to evaluate powder crystallinity of 4 wt.% hydrogels incubated at 37 °C for 1 h, ultra-rapidly frozen using liquid nitrogen, and then lyophilized. DSC was used to test solution crystallinity of 2 wt.% polymer solutions. Critical micellar concentrations (CMC) were measured by quantifying ratiometric emitted fluorescence (373 nm and 383 nm) of pyrenes at an excitation wavelength of 336 nm when 50 µL of different concentrations of polymers were incubated with 50 µL of 1.2 µM pyrene for 1 day. MCR302 rheometer (Anton Paar) using Rheocompass v1.24.584 software was employed to investigate rheology of F127-*g*-Gelatin hydrogels. A Hitachi SU-8230 at accelerating voltage 1 kV and 10 μA emission current was used to obtain scanning electron microscopy (SEM) images of F127-*g*-Gelatin hydrogel, which were lyophilized after incubation of 4.5 wt.% polymer solutions at 37 °C and ultra-rapidly frozen using liquid nitrogen. Size and zeta potential of in situ micelles were assessed by dynamic light scattering (DLS) and Zetasizer Nano ZS (Malvern Instruments) using Zetasizer v5.1 software, in which final concentrations of F127-*g*-Gelatin and aCTLA-4 after total release from 4.5 wt.% F127-*g*-Gelatin hydrogel were 0.9 wt.% and 0.542 mg mL^−1^, respectively. Morphology of in situ micelles was analyzed by TEM imaging of 4.5 wt.% F127-*g*-Gelatin hydrogel (300 μL) supernatants incubated in saline (300 μL) at 37 °C for 1 d with high-resolution FEI Tecnai G2 F30 TEM (FEI Company) using Gatan GMS v3.2 software. Fluorescence resonance energy transfer (FRET) assays was performed by fluorescence measurements at FITC excitation (495 nm) and TRITC emission (572 nm) wavelengths using a Synergy H4 microplate reader (BioTek) using Gen5 v2.09 software. In FRET assays, negligible fluorescence was detected in aCTLA-4-TRITC. Compared to F127-*g*-Gelatin-FITC, the fluorescence signal was significantly increased in the mixture of aCTLA-4-TRITC and F127-*g*-Gelatin-FITC, which was proportional to the concentration of aCTLA-4-TRITC.

### In vitro residence stability of and release of NO_x_ and GSNO, and aCTLA-4 from polymer hydrogel

Three hundred microliters of F127-*g*-Gelatin 4.5 wt.% hydrogels containing GSNO (Sigma Aldrich) [at a final GSNO concentration of 0.45 mg mL^−1^ (1.34 mM)] or aCTLA-4-AF647 (at a final aCTLA-4 mAb concentration of 0.542 mg mL^−1^, clone: 9H10) were prepared in 1.5 mL e-tube in 37 °C water incubator to which an additional 300 µL of PBS with or without 2.5 U mL^−1^ MMP9 (Gibco^TM^, collagenase IV) was added. After supernatants were sampled with a pipet at predetermined time intervals due to the solid hydrogel being easily distinguishable from the liquid supernatant, the remaining hydrogel masses were recorded in their hydrated condition prior to the re-addition of fresh 300 µL of PBS with or without 2.5 U mL^−1^ MMP9. It is important to note that the frequency of sampling significantly affects the results of in vitro hydrogel residence stability and drug release test. Griess/Saville assay with mercuric chloride (HgCl_2_) to reduce *S*-nitrosothiols was employed to measure total nitrite (NO_x_) and GSNO contents in harvested supernatants, while Alexa Fluor 647 fluorescence (650 nm excitation, 670 nm emission) was recorded from the supernatants using a Synergy H4 microplate to measure aCTLA-4 mAb content. As the polymer and degradants detached/released from hydrogel take up volume, each absorbance of Griess assay/Saville assay and fluorescence of aCTLA-4-AF647 were multiplied with each corresponding sample volume to calculate the total released GSNO and aCTLA-4.

### Cell lines

B16F10-OVA murine melanoma, 4T1 murine mammary carcinoma, and NIH3T3 murine fibroblast cells were provided from Prof. Melody Swartz previously at École Polytechnique Fédérale de Lausanne, Prof. Edmund Waller in Emory University, and Prof. Andres Garcia in Georgia Institute of Technology, respectively.

### mAb activity test

The activity of aCTLA-4 mAb (clone: 9H10) released from F127-*g*-Gelatin hydrogel was evaluated by using a competitive binding assay. In brief, aCTLA-4 mAb and 4.5 wt.% F127-*g*-Gelatin containing aCTLA-4 mAb (at a final aCTLA-4 mAb concentration of 0.88 mg mL^−1^) in Dulbecco’s Modified Eagle Medium (Gibco^TM^, DMEM) containing 10% Fetal Bovine Serum (Gibco^TM^, FBS) and 1X Antibiotic-Antimycotic (Gibco^TM^) were incubated in 37 ˚C water incubator until complete gel disruption (4 days). B16F10 cells that endogenously express CTLA-4 were plated at 5 × 10^3^ cells well^−1^ in 96 well U-bottom non-cell culture plates (Falcon^®^), incubated with 2.4G2 (Tonbo bioscience) on ice for 5 min, and stained with Zombie Aqua fixable viability dye (Biolegend) at room temperature for 30 min. The cells were incubated with free as-prepared aCTLA-4 mAb solutions, or aCTLA-4 mAb containing degraded F127-*g*-Gelatin hydrogel solutions for 30 min on ice, followed by incubation in flow cytometry staining buffer (10 mg ml^−1^ bovine serum albumin (Sigma Aldrich) in PBS, FACS buffer) or aCTLA-4-BV605 (Biolegend, clone: UC10-4B9) in FACS buffer for 30 min on ice. Cells were fixed with 2% paraformaldehyde in PBS (Alfa Aesar) on ice for 15 min. Cells were washed with PBS or FACS buffers after each step. LSR Fortessa flow cytometry (BD Biosciences) and flowJo (FlowJo LLC) were employed to measure and analyze cell staining. The decreased fluorescence value is proportional to the binding activity of the pretreated aCTLA-4.

### In vitro cell cytotoxicity

DMEM containing 10% FBS and 1X Antibiotic-Antimycotic was used to culture B16F10-OVA mouse melanoma and NIH3T3 mouse fibroblast cells. Ninety microliters of 10^3^ B16F10-OVA cells in complete medium was seeded in the 96 well cell culture plates (Falcon^TM^), followed by incubation in 37 °C CO_2_ incubator overnight for Supplementary Fig. 21 and Supplementary Fig. 23. Cells in 90 µL medium were treated with 10 µL of GSNO, aCTLA-4 mAb (clone: 9H10), or GSNO + aCTLA-4 mAb suspensions prepared at 10-fold higher than the final concentrations, followed by incubation in 37 °C CO_2_ incubator during 2 days for Supplementary Fig. 21 and Supplementary Fig. 23. Ninety microliters of 10^4^ B16F10-OVA or NIH3T3 cells in complete medium was seeded in the 96 well cell culture plates, followed by incubation in 37 °C CO_2_ incubator overnight for Supplementary Fig. 38. Cell medium in the 96 well cell culture plates were replaced with 100 µL medium containing various concentrations of dissolved F127-*g*-Gelatin, followed by incubation in 37 °C CO_2_ incubator during 2 days for Supplementary Fig. 38. Fluorescence (560 nm excitation, 590 nm emission) was recorded by Synergy H4 microplate reader after 1 h incubation of the cells with 5 µL of alamarBlue^TM^ cell viability reagent (Invitrogen^TM^) in 37 °C CO_2_ incubator.

### Animal ethics

All animal procedures were IACUC approved and performed in Georgia Tech’s Physiological Research Laboratory (PRL). C57Bl/6 and Balb/C mice were purchased from Jackson Laboratories. Mice were housed in the ventilated cage (max 5 mice/cage) supplied with food and water in a 12-h light/12-h dark cycle (7:00–19:00 light and 19:00–7:00 dark) at 22 °C and 41% humidity. Mice were withdrawn from the study and sacrificed when the IACUC stipulated humane endpoint was reached: hunched appearance, more than 10% body weight loss, or tumor size in any dimension reaching 1.5 cm.

### Hydrogel in vivo residence stability and aCTLA-4 mAb release

Free aCTLA-4-AF647 or 4.5 wt.% F127-*g*-Gelatin hydrogels containing aCTLA-4-AF647 (aCTLA-4 mAb dose equivalent to 26.6 µg mouse^−1^, clone: 9H10) were administered to the left dorsal skin of mice (8–10-week-old female C57Bl/6). Hydrogel size was calculated as cuboidal volume, with each dimension measured by calipers. Fluorescence of aCTLA-4-AF647 at the injection site was quantified using IVIS^®^ Spectrum (Perkin Elmer), which represented the quantity of aCTLA-4-AF647 not released from hydrogel.

### In vivo biodistribution of aCTLA-4

Thirty microliters of 10^5^ B16F10-OVA cells in saline was inoculated in the left dorsal skin of mice (8–10 weeks old female C57Bl/6) on day 0 and in right dorsal skin on day 4 after which time either 30 µL of free aCTLA-4-AF647 (clone: 9H10), F127-*g*-Gelatin micelles (0.45 wt.%) containing aCTLA-4-AF647, or 4.5 wt.% F127-*g*-Gelatin hydrogels containing aCTLA-4-AF647 (aCTLA-4 dose equivalent to 162 µg mouse^−1^) was administered into the left tumor on day 7. Mice were sacrificed on day 8, 14 and 18 (equivalent to day 1, 7, and 11 after treatment). Harvested tissues were homogenized in 1.4 mm zirconium bead-filled tubes (OPS Diagnostics) with FastPrep-24 (MP Biomedicals), and the fluorescence (650 nm excitation, 670 nm emission) was recorded by Synergy H4 microplate reader. Standard curves of aCTLA-4-AF647 for each tissue were established by recording fluorescence of different concentrations of aCTLA-4-AF647 added to homogenized tissues harvested from untreated tumor-bearing mice.

### In vivo tumor therapy

10^5^ B16F10-OVA cells in 30 µL saline was inoculated in left dorsal skin of C57Bl/6 mice (female, 8–10 weeks old) on day 0 and, and then 200 µL of saline or GSNO (600 µg kg^−1^) was administered intravenously on day 4, 6, and 8 for Supplementary Fig. 20. Thirty microliters of 10^5^ B16F10-OVA cells in saline was inoculated in left dorsal skin of 8–10 week old female C57Bl/6 mice on day 0 and in right dorsal skin of these same animals on day 3 after which time 30 µL of either saline or GSNO (570 µg kg^−1^) was administered to the left tumor on day 6, 8, and 10 for Fig. 2a–e. Thirty microliters of 10^5^ B16F10-OVA cells in saline was inoculated in left dorsal skin of 8–10 week old female C57Bl/6 mice on day 0 and in right dorsal skin of these same animals on day 4 after which time 30 µL of either saline or GSNO (480 µg kg^−1^) was administered to the left tumor on day 7 and 30 µL of aCTLA-4 (100 µg mouse^−1^, clone: 9H10) was administered intraperitoneally on day 8, 11, 14 for Fig. 3. Thirty microliters of 10^5^ B16F10-OVA cells in saline was inoculated in left dorsal skin of 8–10 week old female C57Bl/6 mice on day 0 and in right dorsal skin of these same animals on day 4 after which 30 µL of either saline, free GSNO, free aCTLA-4, free GSNO + free aCTLA-4, F127-*g*-Gelatin hydrogel (HG), GSNO containing hydrogel (GSNO/HG), aCTLA-4 containing hydrogel (aCTLA-4/HG), or F127-*g*-Gelatin hydrogel containing GSNO and aCTLA-4 (GSNO + aCTLA-4/HG) (all F127-g-Gelatin hydrogel was 4.5 wt.%. GSNO and aCTLA-4 (clone: 9H10) dose equivalent to 570 µg kg^−1^ and 50 µg mouse^−1^, respectively) was administered to the left tumor on day 7 for Fig. 6. Thirty microliters of 3 × 10^5^ 4T1 cells in saline was inoculated in left mammary fat pad on day 0 and in right mammary fat pad of Balb/C mice (female, 6–12 weeks old) on day 4 after which 30 µL of either saline, free GSNO + free aCTLA-4, F127-*g*-Gelatin hydrogel (HG), or F127-*g*-Gelatin hydrogel containing GSNO and aCTLA-4 (GSNO + aCTLA-4/HG) (all F127-g-Gelatin hydrogel was 4.5 wt.%. GSNO and aCTLA-4 (clone: 9D9) dose equivalent to 570 µg kg^−1^ and 50 µg mouse^−1^, respectively) was administered to the left tumor on day 7 for Fig. 7. The tumor size for Figs. 2, 3 and Supplementary Fig. 20 was calculated by a cuboidal volume (*V* = abc, where *a* is height, *b* is width, and *c* is length, respectively) with each dimension measured by caliper. Due to the added size in the 1^o^ tumor resulting from the hydrogel’s i.t. administration, tumor size in Fig. 6 and Fig. 7 was calculated by an ellipsoid volume (*WL*^2^/2, where *W* and *L* are the longer and shorter dimensions, respectively). Animal survival was analyzed by Kaplan–Meier curves.

### Tissue and blood harvest for immune cell profiles

Draining lymph nodes (dLNs), non-draining lymph nodes (ndLNs) and spleens were harvested 1 day after injection of 30 µL of either saline and GSNO (0.38 mg mL^−1^ (1.13 mM), GSNO dose equivalent to 570 µg kg^−1^) into the left dorsal skin of 8–10 weeks old tumor-free mice (female C57Bl/6) for Fig. 1 and Supplementary Figs. 1–19. Thirty microliters of 10^5^ B16F10-OVA cells in saline was inoculated in left dorsal skin of mice (female C57Bl/6 with 8–10 weeks old) on day 0 and in right dorsal skin of the mice on day 4 after which time 30 µL of either saline or GSNO [0.38 mg mL^−1^ (1.13 mM), GSNO dose equivalent to 570 µg kg^−1^] was administered in the left tumor on day 7 for Fig. 2f–j and Supplementary Fig. 22. 1^o^ and 2^o^ tumors were harvested after animal sacrifice on day 8 for Fig. 2f–j and Supplementary Fig. 22. Blood used in immune profiling measurements was collected from the facial vein day 13 post tumor inoculation (Fig. 3f–i).

### Immune cell profiling

Splenocytes were harvested by passing through the spleens with 70 µm strainer (Corning) and incubating in ACK lysis buffer (Lonza). Lymphocytes were harvested by passing through the lymph nodes with 70 µm strainer after incubating each lymph node in collagenase D (Roche, 1 mg mL^−1^) in 37 °C CO_2_ incubator for 75 min. Cells within tumors were harvested by passing tumors through a 70 µm strainer after incubation in collagenase D at 37 °C for 4 h. Red blood cells were lysed using ACK lysis buffer per the manufacturer’s protocol. All cells were stored on ice <2 h prior to use. Cells for flow cytometry were prepared by staining with 2.4G2 on ice for 5 min, staining with Zombie Aqua fixable viability dye at room temperature for 30 min, staining with or without SIINFEKL-MHCI–PE tetramer (NIH Tetramer Core Facility, Atlanta, Georgia) on ice for 15 min, staining with antibody mixtures on ice for 30 min, fixing and permeabilizing with Foxp3 Fixation/Permeabilization working solution (eBioscience^TM^ Foxp3/Transcription Factor Staining Buffer Set, Invitrogen^TM^) on ice for 60 min, and staining FoxP3 and/or Ki-67 on ice for 75 min. Cells were washed with PBS, FACS buffer, or permeabilization buffer (eBioscience^TM^ Foxp3/Transcription Factor Staining Buffer Set, Invitrogen^TM^) after each step. LSR Fortessa flow cytometry (BD FACSDiva v9.0 software) and FlowJo (v10.6) were employed to analyze and profile the stained cells. The information for staining antibodies and dilutions that were used is listed in Supplementary Table 13–15.

### In vivo ALT/AST analysis

Blood was collected from facial vein 2 days after subcutaneous injection of 4.5 wt.% F127-*g*-Gelatin hydrogels on mice for Fig. 4l. Blood was collected from the facial vein on day 9 post primary tumor inoculation (equivalent to 2 days after i.t. treatment) for Fig. 6c. Plasma was harvested from collected blood samples after 2x centrifugation at 2100 g at 4 ˚C for 10 min. Alanine aminotransferase (ALT) activity colorimetry/fluorometry (Biovision) and aspartate aminotransferase (AST) activity colorimetric assay kits (Biovision) were purchased from VWR Scientific. ALT/AST assay was performed per the manufacturer’s instructions.

### In vivo hematoxylin and eosin (H&E) staining

Tumors formed in the mammary fat pad of Balb/C mice (female, 6–12 weeks old) by injection of 3 × 10^5^ 4T1 cells were collected 7 days after i.t. injection of 4.5 wt.% F127-*g*-Gelatin hydrogels (14 days after tumor implantation) and frozen in optimum cutting temperature compound (Sakura Finetek USA Inc.) using 2-methylbutane (Sigma Aldrich) chilled with liquid nitrogen. Frozen tissues were sliced, mounted, fixed, stained with Hematoxylin and eosin, and imaged using Nanozoomer 2.0 HT (Hamamatsu, Japan).

### Statistical analysis

In vitro data are expressed as mean±standard deviation (SD), while in vivo data are expressed as mean ± standard error of mean (SEM). Prism software (Graphpad v9) was used for plotting graphs and analyzing the statistical significance of differences among experimental groups. One-way ANOVA and two-way ANOVA with Tukey post-hoc were employed for multiple comparisons, while two-tailed Student *t*-test was used for comparisons of two groups. Log-rank analysis with Mantel–Cox statistical hypothesis were used for survival curve analyses. Tumor growth curves were analyzed with linear mixed-effect regression using R studio (v1.2.5033) with lme4 (v1.1.26) and emmeans (v1.7.2) package. In detail, tumor volumes were transformed to the natural log. Time, treatment groups, and interaction between treatments and time effects were designated as fixed effects, and variability between individual mice was designated as random effects. ANOVA with Tukey post-hoc test was performed in R studio for comparison of tumor growth among each treatment. Details for statistical analyses are indicated in each figure. *****p < 0.0001, ****p < 0.001, ***p < 0.01, **p < 0.05, and *p < 0.1.

### Reporting summary

Further information on research design is available in the Nature Research Reporting Summary linked to this article.

## Supplementary information

Supplementary Information

Reporting Summary

## Data Availability

The source data supporting this study’s findings are available with this paper. The remaining information are available within the Article, Supplementary Information or Source Data files. Source data are provided with this paper.

## Source data

Source data
