# Supplementary material for: Thermosensitive hydrogel releasing nitric oxide donor and anti-CTLA-4 micelles for anti-tumor immunotherapy
Source: Nat Commun. 2022 Mar 18;13:1479. doi: 10.1038/s41467-022-29121-x (PMC8933465; doi:10.1038/s41467-022-29121-x)
Supplement: Supplementary file 1 — Supplementary Information [file 41467_2022_29121_MOESM1_ESM.pdf]

SUPPLEMENTARY INFORMATION

Thermosensitive hydrogel releasing nitric oxide donor and anti-CTLA-4 micelles for anti-tumor immunotherapy

Jihoon Kim[1,2], David M. Francis[1,3], Lauren F. Sestito[4,5], Paul A. Archer[1,3], Margaret P. Manspeaker[1,3], Meghan J. O'Melia[4,5], Susan N. Thomas[1,2,4,5,6*]

[1]Parker H. Petit Institute for Bioengineering and Bioscience, Georgia Institute of Technology, 315 Ferst Dr NW, Atlanta, Georgia 30332, USA

[2]George W. Woodruff School of Mechanical Engineering, Georgia Institute of Technology, 315 Ferst Dr NW, Atlanta, Georgia 30332, USA

[3]School of Chemical and Biomolecular Engineering, Georgia Institute of Technology, 315 Ferst Dr NW, Atlanta, Georgia 30332, USA

[4]Wallace H. Coulter Department of Biomedical Engineering, Georgia Institute of Technology, 313 Ferst Dr NW, Atlanta, Georgia 30332, USA

[5]Wallace H. Coulter Department of Biomedical Engineering, Emory University, 201 Dowman Drive, Atlanta, Georgia 30322, USA

[6]Winship Cancer Institute, Emory University School of Medicine, 1365-C Clifton Road NE, Atlanta, Georgia 30322, USA

[*] Corresponding author: susan.thomas@gatech.edu

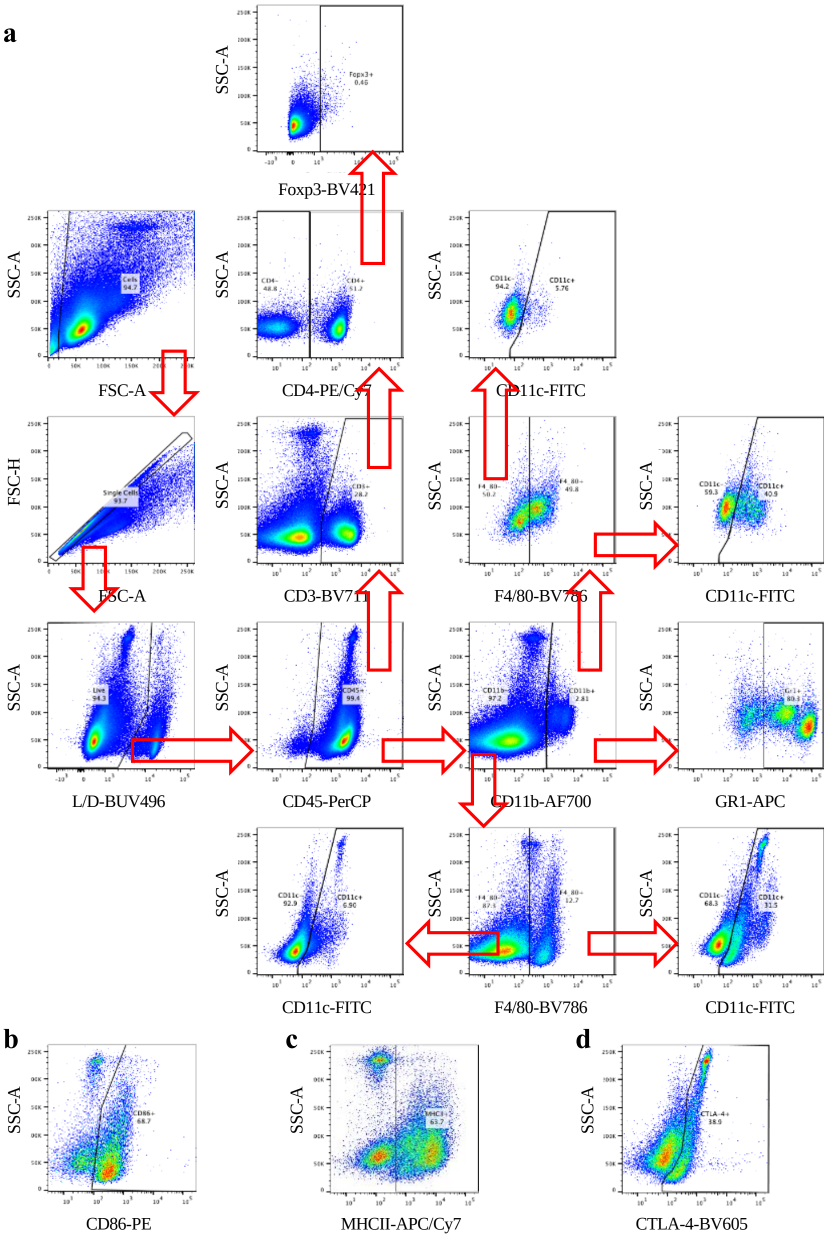

Supplementary Figure 1. Gating strategy for analysis of immune cells in dLN, ndLN, and spleen after subcutaneous GSNO treatment. Immune cells were profiled 1 day after treatment of GSNO (570 μg kg^{-1}) in 30 μL saline. LSR Fortessa flow cytometry and FlowJo were employed to analyze and profiles the stained cells. **a** Gating strategy to identify DCs, macrophages, $CD3^+$ T, $CD4^+$ T, $CD4^-$ T, T_{reg}s, and MDSCs. **b-d** Gating strategy to determine positive signal of (**b**) CD86, (**c**) MHCII, and (**d**) CTLA-4 staining.

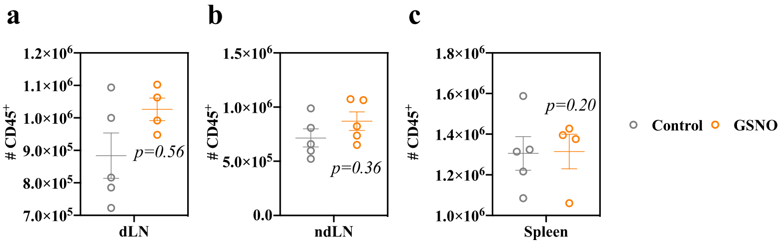

Supplementary Figure 2. Total number of CD45$^+$ immune cells in dLN, ndLN, and spleen associated with subcutaneous GSNO treatment. Immune cells were profiled 1 day after treatment of GSNO (570 µg kg^{-1}) in 30 µL saline. **a-c** Number of CD45$^+$ in (**a**) dLN, (**b**) ndLN, and (**c**) spleen. Data are presented as individual biological replicates and mean±SEM. **a, c** n=5 for control and n=4 for GSNO. **b** n=5. *****p < 0.0001, ****p < 0.001, ***p < 0.01, **p < 0.05, and *p < 0.1 with two-tailed Student t-test. Source data are available in a Source Data file.

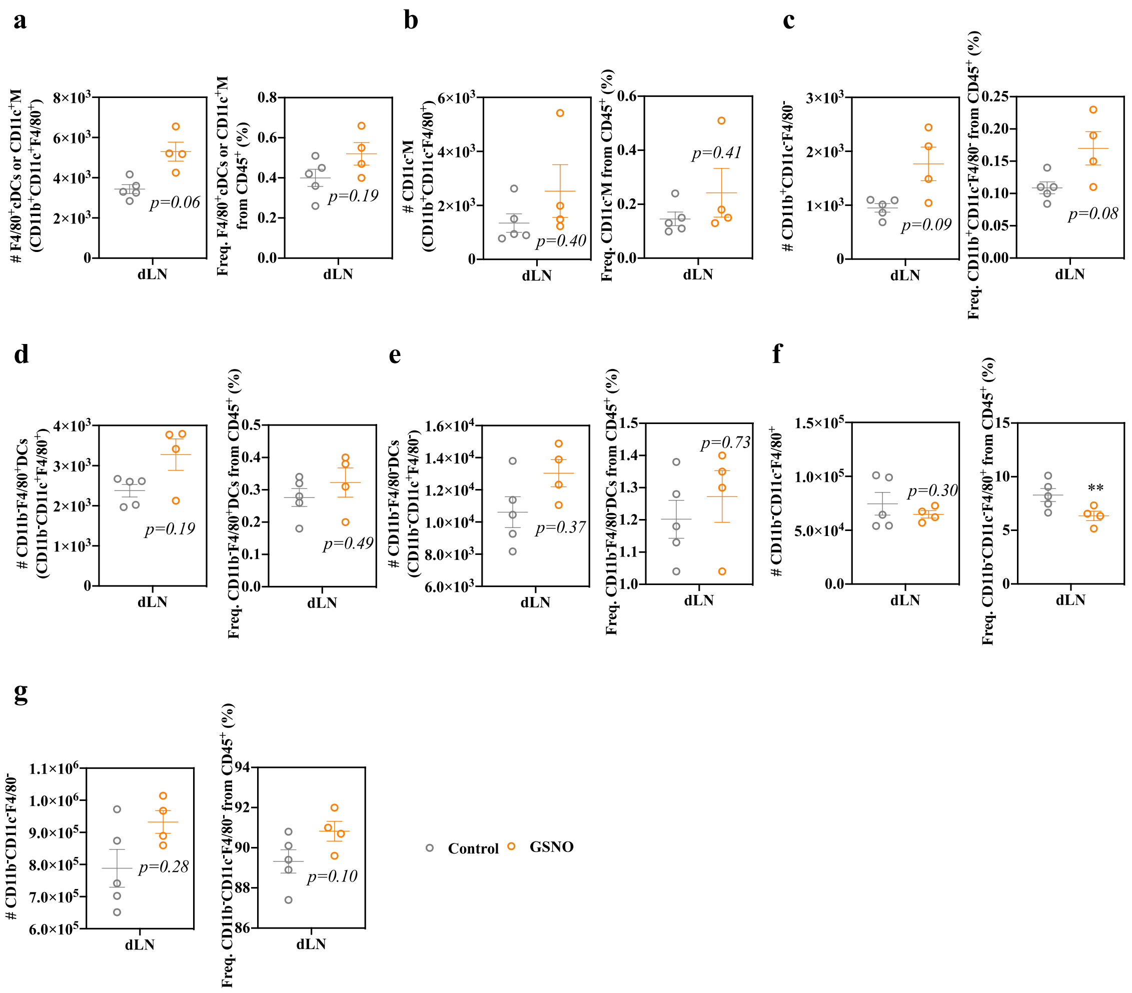

Supplementary Figure 3. Profiles of CD11b, CD11c and/or F4/80 expressing immune cells in dLN associated with subcutaneous GSNO treatment. Immune cells in dLN were profiled 1 day after treatment of GSNO (570 μg kg^{-1}) in 30 μL saline. **a-g** Number and percent populations of (**a**) $CD45^+CD11b^+CD11c^+F4/80^+$ cDCs or macrophages ($F4/80^+$cDCs or $CD11c^+$M), (**b**) $CD45^+CD11b^+CD11c^-F4/80^+$ macrophages ($CD11c^-$M), (**c**) $CD45^+CD11b^+CD11c^-F4/80^-$ (**d**) $CD45^+CD11b^-CD11c^+F4/80^+$ DCs ($CD11b^-F4/80^+$DCs), (**e**) $CD45^+CD11b^-CD11c^+F4/80^-$ DCs ($CD11b^-F4/80^-$DCs), (**f**) $CD45^+CD11b^-CD11c^-F4/80^+$, and (**g**) $CD45^+CD11b^-CD11c^-F4/80^-$. Data are presented as individual biological replicates and mean±SEM. n=5 for control and n=4 for GSNO. *****$p < 0.0001$, ****$p < 0.001$, ***$p < 0.01$, **$p < 0.05$, and *$p < 0.1$ with two-tailed Student t-test. Source data are available in a Source Data file.

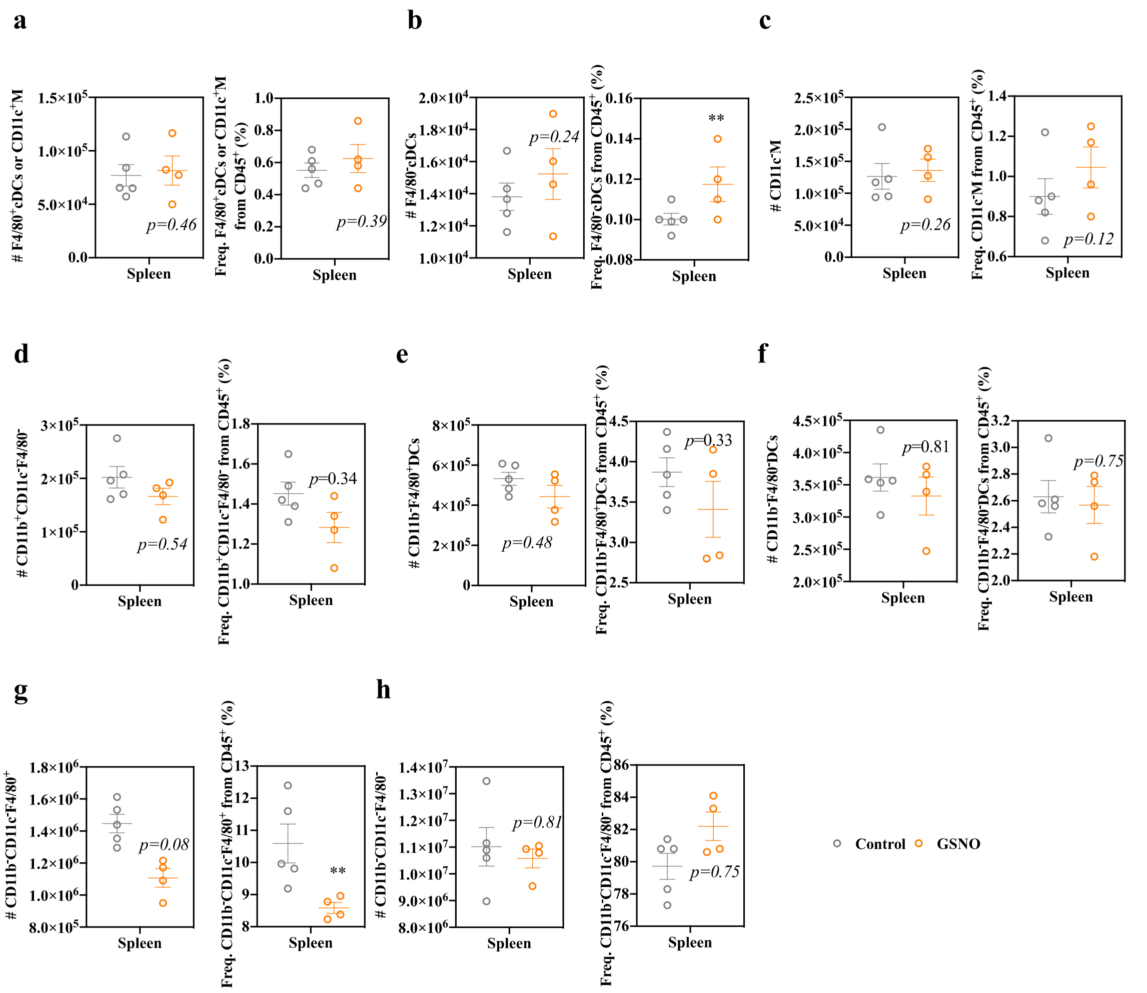

Supplementary Figure 4. Profiles of CD11b, CD11c and/or F4/80 expressing immune cells in spleen associated with subcutaneous GSNO treatment. Immune cells in spleen were profiled 1 day after treatment of GSNO (570 μg kg^{-1}) in 30 μL saline. **a-h** Number and percent populations of (**a**) $F4/80^{+}$cDCs or $CD11c^{+}$M, (**b**) $F4/80^{-}$cDCs, (**c**) $CD11c^{-}$M, (**d**) $CD11b^{+}CD11c^{-}F4/80^{-}$, (**e**) $CD11b^{-}F4/80^{+}$DCs, (**f**) $CD11b^{-}F4/80^{-}$DCs, (**g**) $CD11b^{-}CD11c^{-}F4/80^{+}$, and (**h**) $CD11b^{-}CD11c^{-}F4/80^{-}$. Data are presented as mean±SEM. n=5 for control and n=4 for GSNO. *****$p < 0.0001$, ****$p < 0.001$, ***$p < 0.01$, **$p < 0.05$, and *$p < 0.1$ with two-tailed Student t-test. Source data are available in a Source Data file.

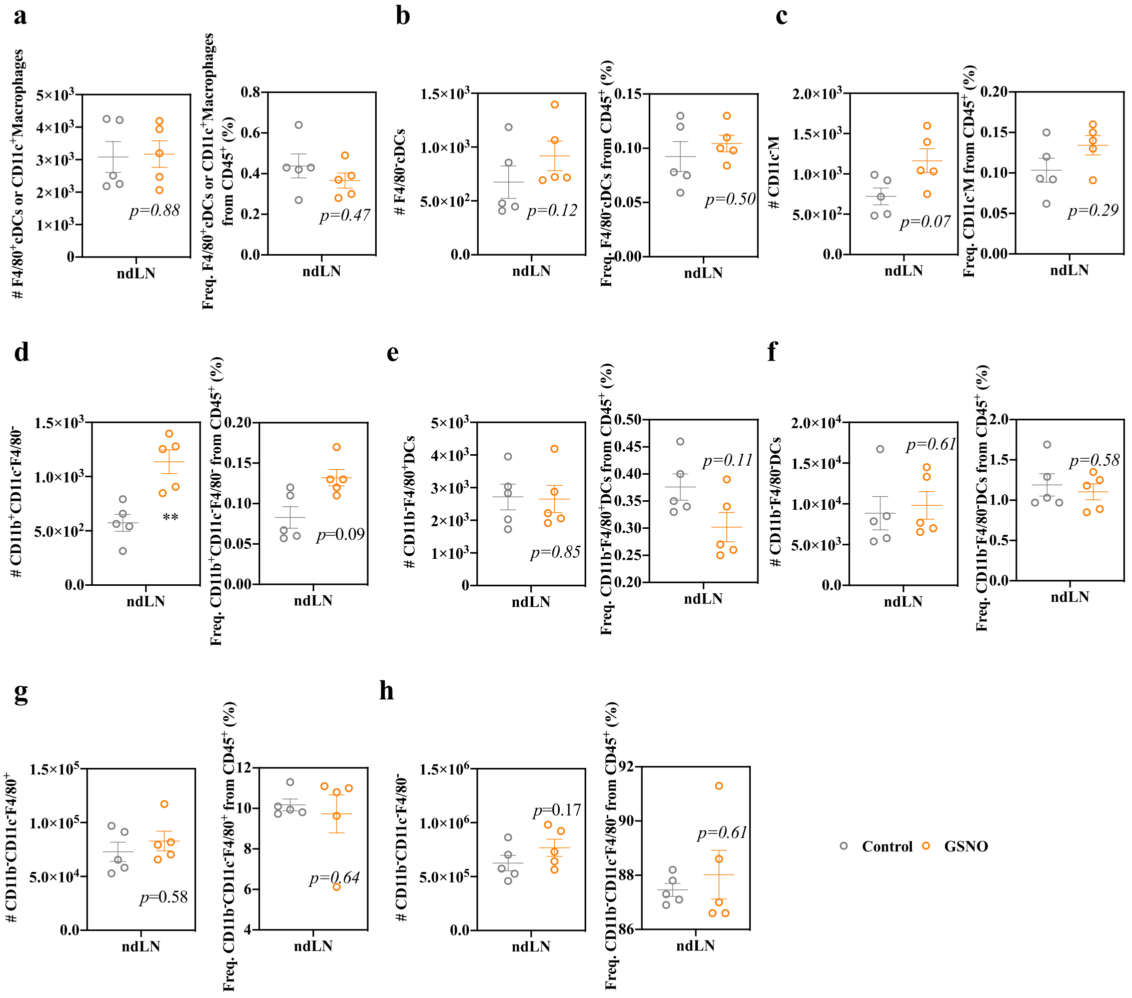

Supplementary Figure 5. Profiles of CD11b, CD11c and/or F4/80 expressing immune cells in ndLN associated with subcutaneous GSNO treatment. Immune cells in ndLN were profiled 1 day after treatment of GSNO (570 μg kg^{-1}) in 30 μL saline. **a-h** Number and percent populations of (**a**) F4/80$^+$cDCs or CD11c$^+$M, (**b**) F4/80$^-$cDCs, (**c**) CD11c$^-$M, (**d**) CD11b$^+$CD11c$^-$F4/80$^-$, (**e**) CD11b$^-$F4/80$^+$DCs, (**f**) CD11b$^-$F4/80$^-$DCs, (**g**) CD11b$^-$CD11c$^-$F4/80$^+$, and (**h**) CD11b$^-$CD11c$^-$F4/80$^-$. Data are presented as individual biological replicates and mean±SEM. n=5. *****p < 0.0001, ****p < 0.001, ***p < 0.01, **p < 0.05, and *p < 0.1 with two-tailed Student t-test. Source data are available in a Source Data file.

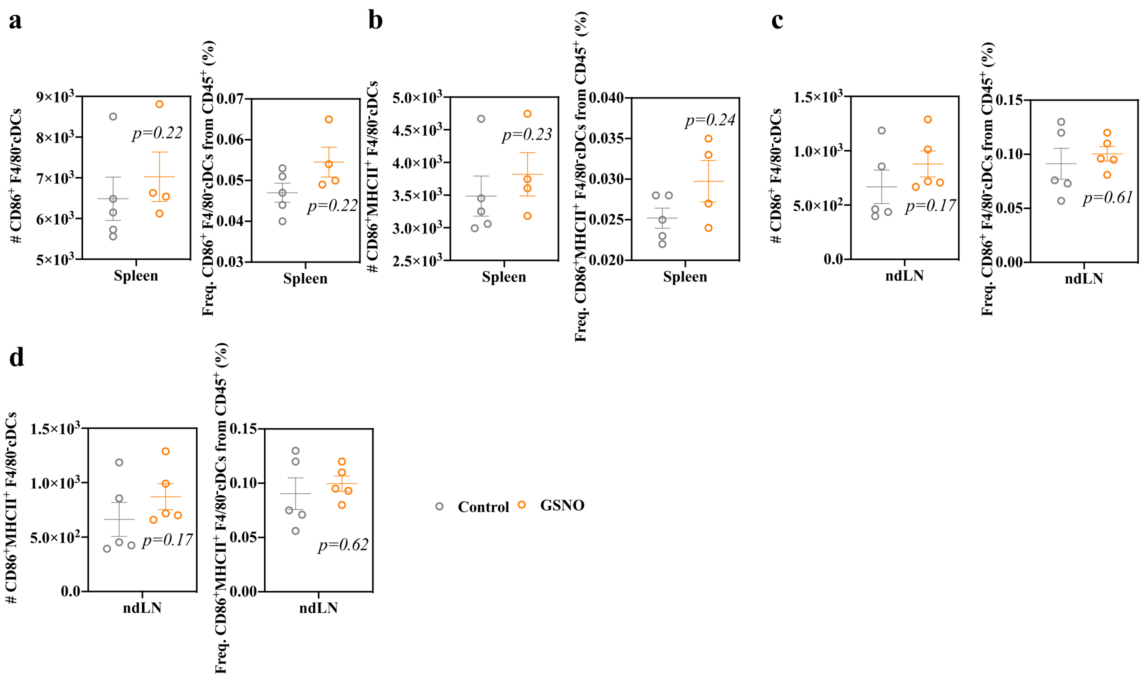

Supplementary Figure 6. Profiles of activated F4/80$^-$cDCs in spleen and ndLN associated with subcutaneous GSNO treatment. Immune cells in spleen and ndLN were profiled 1 day after treatment of GSNO (570 µg kg^{-1}) in 30 µL saline. **a-d** Number and percent populations of (**a**) CD86$^+$ activated F4/80$^-$cDCs (CD86$^+$ F4/80$^-$cDCs) in spleen, (**b**) CD86$^+$ and MHCII$^+$ activated F4/80$^-$cDCs (CD86$^+$MHCII$^+$ F4/80$^-$cDCs) in spleen, (**c**) CD86$^+$ F4/80$^-$cDCs in ndLN, and (**d**) CD86$^+$MHCII$^+$ F4/80$^-$cDCs in ndLN. Data are presented as individual biological replicates and mean±SEM. **a, b** n=5 for control and n=4 for GSNO. **c, d** n=5. *****$p < 0.0001$, ****$p < 0.001$, ***$p < 0.01$, **$p < 0.05$, and *$p < 0.1$ with two-tailed Student t-test. Source data are available in a Source Data file.

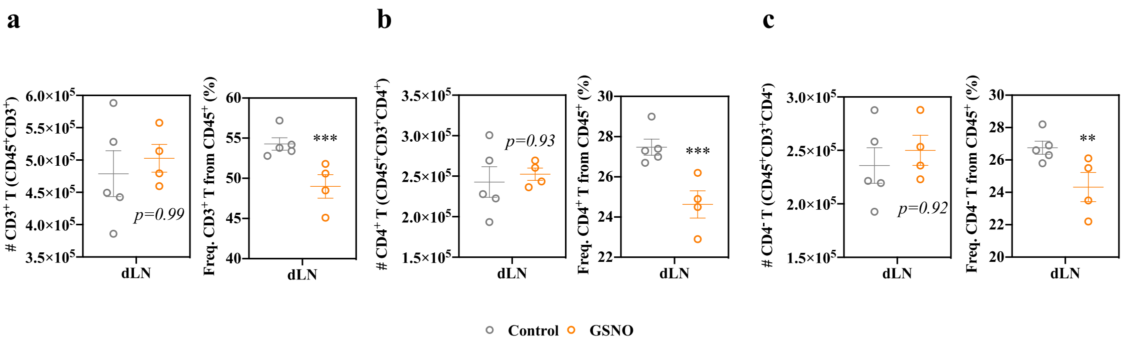

Supplementary Figure 7. Profiles of $CD3^+$, $CD4^+$, and $CD4^-$ T cells in dLN associated with subcutaneous GSNO treatment. Immune cells in dLN were profiled 1 day after treatment of GSNO (570 μg kg^{-1}) in 30 μL saline. **a-c** Number and percent populations of (**a**) $CD45^+CD3^+$ ($CD3^+$ T) (right $p = 0.0073$), (**b**) $CD45^+CD3^+CD4^+$ ($CD4^+$ T) (right $p = 0.0034$), and (**c**) $CD45^+CD3^+CD4^-$ ($CD4^-$ T) (right $p = 0.0251$) cells. Data are presented as individual biological replicates and mean±SEM. n=5 for control and n=4 for GSNO. *****$p < 0.0001$, ****$p < 0.001$, ***$p < 0.01$, **$p < 0.05$, and *$p < 0.1$ with two-tailed Student t-test. Source data are available in a Source Data file.

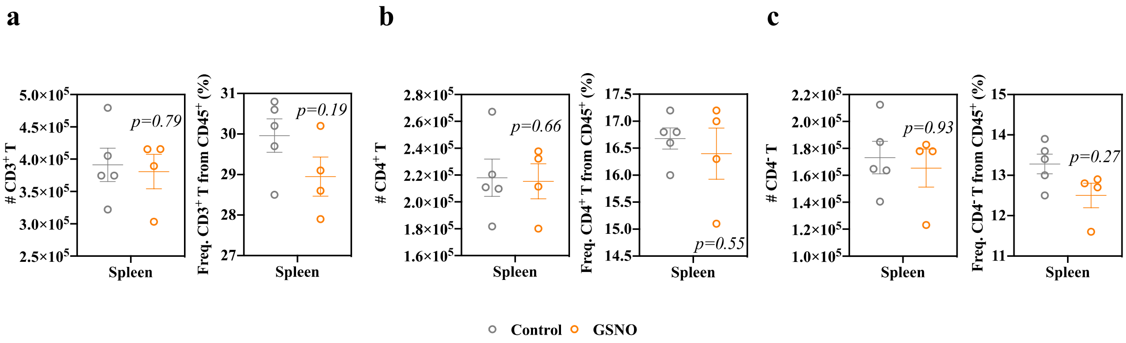

Supplementary Figure 8. Profiles of $CD3^+$, $CD4^+$, and $CD4^-$ T cells in spleen associated with subcutaneous GSNO treatment. Immune cells in spleen were profiled 1 day after treatment of GSNO (570 μg kg^{-1}) in 30 μL saline. **a-c** Number and percent populations of (**a**) $CD3^+$ T, (**b**) $CD4^+$ T, and (**c**) $CD4^-$ T cells. Data are presented as individual biological replicates and mean±SEM. n=5 for control and n=4 for GSNO. *****$p < 0.0001$, ****$p < 0.001$, ***$p < 0.01$, **$p < 0.05$, and *$p < 0.1$ with two-tailed Student t-test. Source data are available in a Source Data file.

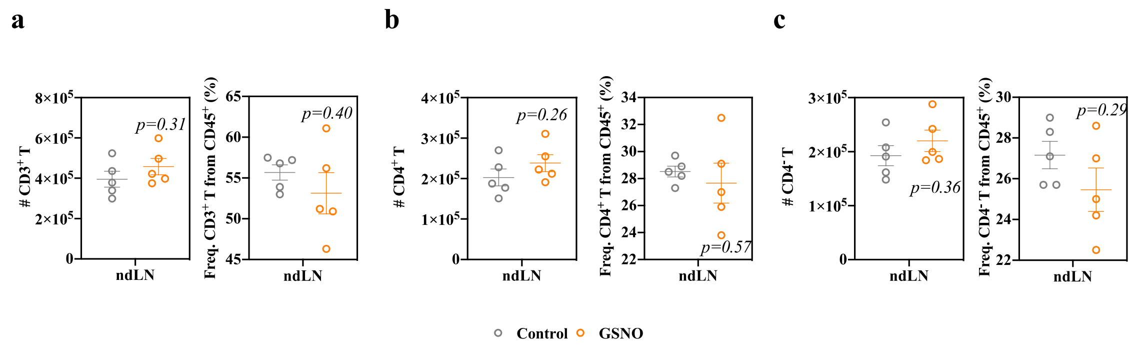

Supplementary Figure 9. Profiles of CD3$^+$, CD4$^+$, and CD4$^-$ T cells in ndLN associated with subcutaneous GSNO treatment. Immune cells in ndLN were profiled 1 day after treatment of GSNO (570 μg kg^{-1}) in 30 μL saline. **a-c** Number and percent populations of (**a**) CD3$^+$ T, (**b**) CD4$^+$ T, and (**c**) CD4$^-$ T cells. Data are presented as individual biological replicates and mean±SEM. n=5. *****p < 0.0001, ****p < 0.001, ***p < 0.01, **p < 0.05, and *p < 0.1 with two-tailed Student t-test. Source data are available in a Source Data file.

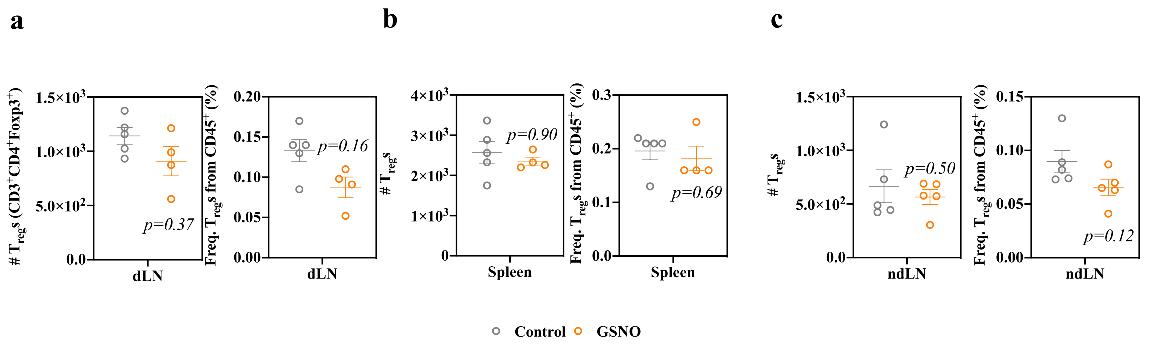

Supplementary Figure 10. Profiles of T_{reg}s in dLN, spleen, and ndLN when GSNO is subcutaneously treated to tumor-free mice. Immune cells in dLN, spleen, and ndLN were profiled 1 day after treatment of GSNO (570 μg kg^{-1}) in 30 μL saline. **a-c** Number and percent populations of CD45$^+$CD3$^+$CD4$^+$Foxp3$^+$ (T_{reg}s) in (**a**) dLN, (**b**) spleen, and (**c**) ndLN. Data are presented as mean±SEM. **a, b** n=5 for control and n=4 for GSNO. **c** n=5. *****p < 0.0001, ****p < 0.001, ***p < 0.01, **p < 0.05, and *p < 0.1 with two-tailed Student t-test. Source data are available in a Source Data file.

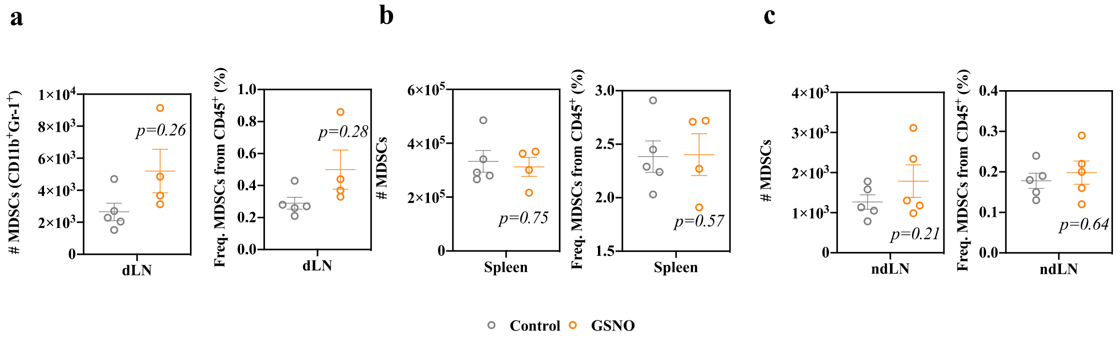

Supplementary Figure 11. Profiles of MDSCs in dLN, spleen, and ndLN associated with subcutaneous GSNO treatment. Immune cells in dLN, spleen, and ndLN were profiled 1 day after treatment of GSNO (570 μg kg^{-1}) in 30 μL saline. **a-c** Number and percent populations of $CD45^+CD11b^+Gr1^+$ (MDSCs) in (**a**) dLN, (**b**) spleen, and (**c**) ndLN. Data are presented as individual biological replicates and mean±SEM. **a, b** n=5 for control and n=4 for GSNO. **c** n=5. *****$p < 0.0001$, ****$p < 0.001$, ***$p < 0.01$, **$p < 0.05$, and *$p < 0.1$ with two-tailed Student t-test. Source data are available in a Source Data file.

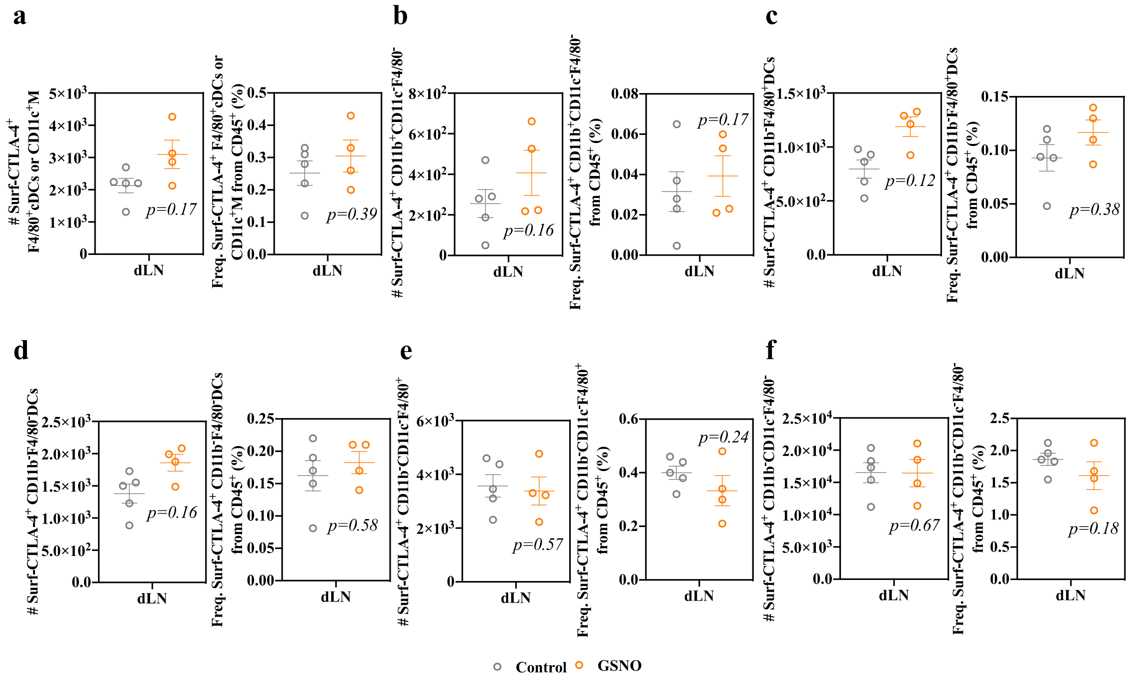

Supplementary Figure 12. Profiles of extracellular CTLA-4 expression of CD11b, CD11c and/or F4/80 expressing immune cells in dLN associated with subcutaneous GSNO treatment. Immune cells in dLN were profiled 1 day after treatment of GSNO (570 μg kg^{-1}) in 30 μL saline. **a-f** Number and percent populations of extracellular CTLA-4 expressing (**a**) $F4/80^+$cDCs or $CD11c^+$M (Surf-CTLA-4^+ $F4/80^+$cDCs or $CD11c^+$M), (**b**) $CD11b^+CD11c^-F4/80^-$ (Surf-CTLA-4^+ $CD11b^+CD11c^-F4/80^-$), (**c**) $CD11b^-F4/80^+$DCs (Surf-CTLA-4^+ $CD11b^-F4/80^+$DCs), (**d**) $CD11b^-F4/80^-$DCs (Surf-CTLA-4^+ $CD11b^-F4/80^-$DCs), (**e**) $CD11b^-CD11c^-F4/80^+$ (Surf-CTLA-4^+ $CD11b^-CD11c^-F4/80^+$), and (**f**) $CD11b^-CD11c^-F4/80^-$ (Surf-CTLA-4^+ $CD11b^-CD11c^-F4/80^-$). Data are presented as individual biological replicates and mean±SEM. n=5 for control and n=4 for GSNO. *****$p < 0.0001$, ****$p < 0.001$, ***$p < 0.01$, **$p < 0.05$, and *$p < 0.1$ with two-tailed Student t-test. Source data are available in a Source Data file.

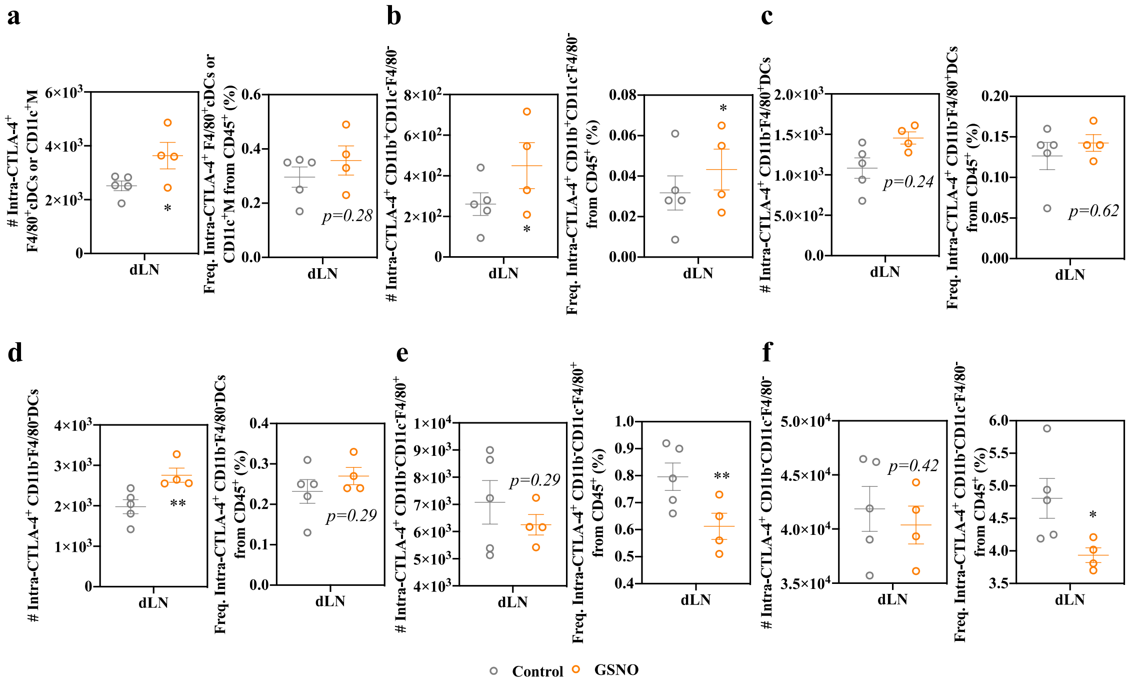

Supplementary Figure 13. Profiles of intracellular CTLA-4 expression of CD11b, CD11c and/or F4/80 expressing immune cells in dLN associated with subcutaneous GSNO treatment. Immune cells in dLN were profiled 1 day after treatment of GSNO (570 μg kg^{-1}) in 30 μL saline. **a-f** Number and percent populations of intracellular CTLA-4 expressing (**a**) $F4/80^+$cDCs or $CD11c^+$M (Intra-CTLA-4^+ $F4/80^+$cDCs or $CD11c^+$M) (left p = 0.0991), (**b**) $CD11b^+CD11c^-F4/80^-$ (Intra-CTLA-4^+ $CD11b^+CD11c^-F4/80^-$) (left p = 0.0966 and right p = 0.0789), (**c**) $CD11b^-F4/80^+$DCs (Intra-CTLA-4^+ $CD11b^-F4/80^+$DCs), (**d**) $CD11b^-F4/80^-$DCs (Intra-CTLA-4^+ $CD11b^-F4/80^-$DCs) (left p = 0.0221), (**e**) $CD11b^-CD11c^-F4/80^+$ (Intra-CTLA-4^+ $CD11b^-CD11c^-F4/80^+$) (right p = 0.0499), and (**f**) $CD11b^-CD11c^-F4/80^-$ (Intra-CTLA-4^+ $CD11b^-CD11c^-F4/80^-$) (right p = 0.0604). Data are presented as individual biological replicates and mean±SEM. n=5 for control and n=4 for GSNO. *****$p < 0.0001$, ****$p < 0.001$, ***$p < 0.01$, **$p < 0.05$, and *$p < 0.1$ with two-tailed Student t-test. Source data are available in a Source Data file.

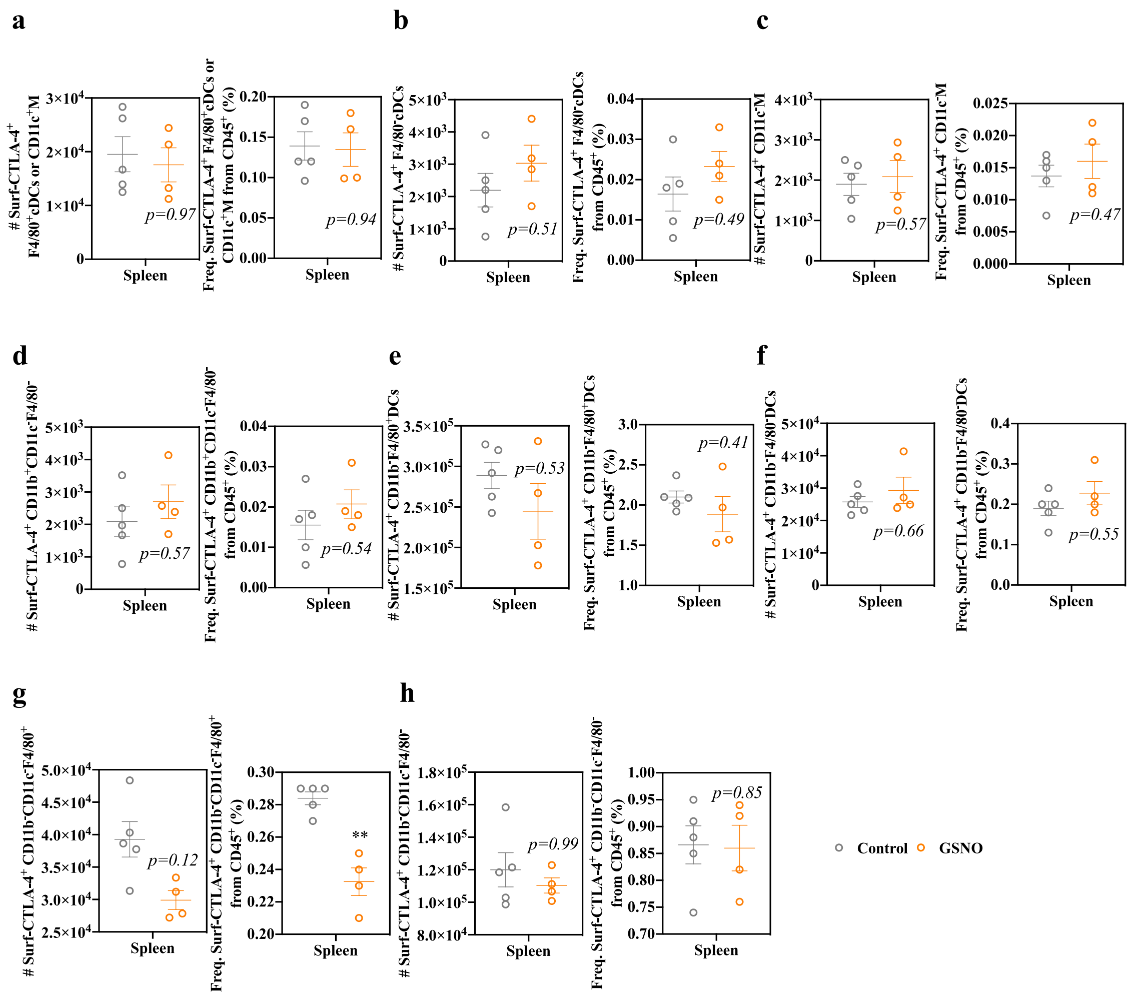

Supplementary Figure 14. Profiles of extracellular CTLA-4 expression of CD11b, CD11c and/or F4/80 expressing immune cells in spleen associated with subcutaneous GSNO treatment. Immune cells in spleen were profiled 1 day after treatment of GSNO (570 μg kg^{-1}) in 30 μL saline. **a-h** Number and percent populations of extracellular CTLA-4 expressing (**a**) $F4/80^+$cDCs or $CD11c^+$ macrophages (Surf-CTLA-4^+ $F4/80^+$cDCs or $CD11c^+$M), (**b**) $F4/80^-$cDCs (Surf-CTLA-4^+ $F4/80^-$cDCs), (**c**) $CD11c^-$M (Surf-CTLA-4^+ $CD11c^-$M), (**d**) $CD11b^+CD11c^-F4/80^-$ (Surf-CTLA-4^+ $CD11b^+CD11c^-F4/80^-$), (**e**) $CD11b^-F4/80^+$DCs (Surf-CTLA-4^+ $CD11b^-F4/80^+$DCs), (**f**) $CD11b^-CD11c^+F4/80^-$ (Surf-CTLA-4^+ $CD11b^-F4/80^-$DCs), (**g**) $CD11b^-CD11c^-F4/80^+$ (Surf-CTLA-4^+ $CD11b^-CD11c^-F4/80^+$) (right p = 0.0190), and (**h**) $CD11b^-CD11c^-F4/80^-$ (Surf-CTLA-4^+ $CD11b^-CD11c^-F4/80^-$). Data are presented as individual biological replicates and mean±SEM. n=5 for control and n=4 for GSNO. *****$p < 0.0001$, ****$p < 0.001$, ***$p < 0.01$, **$p < 0.05$, and *$p < 0.1$ with two-tailed Student t-test. Source data are available in a Source Data file.

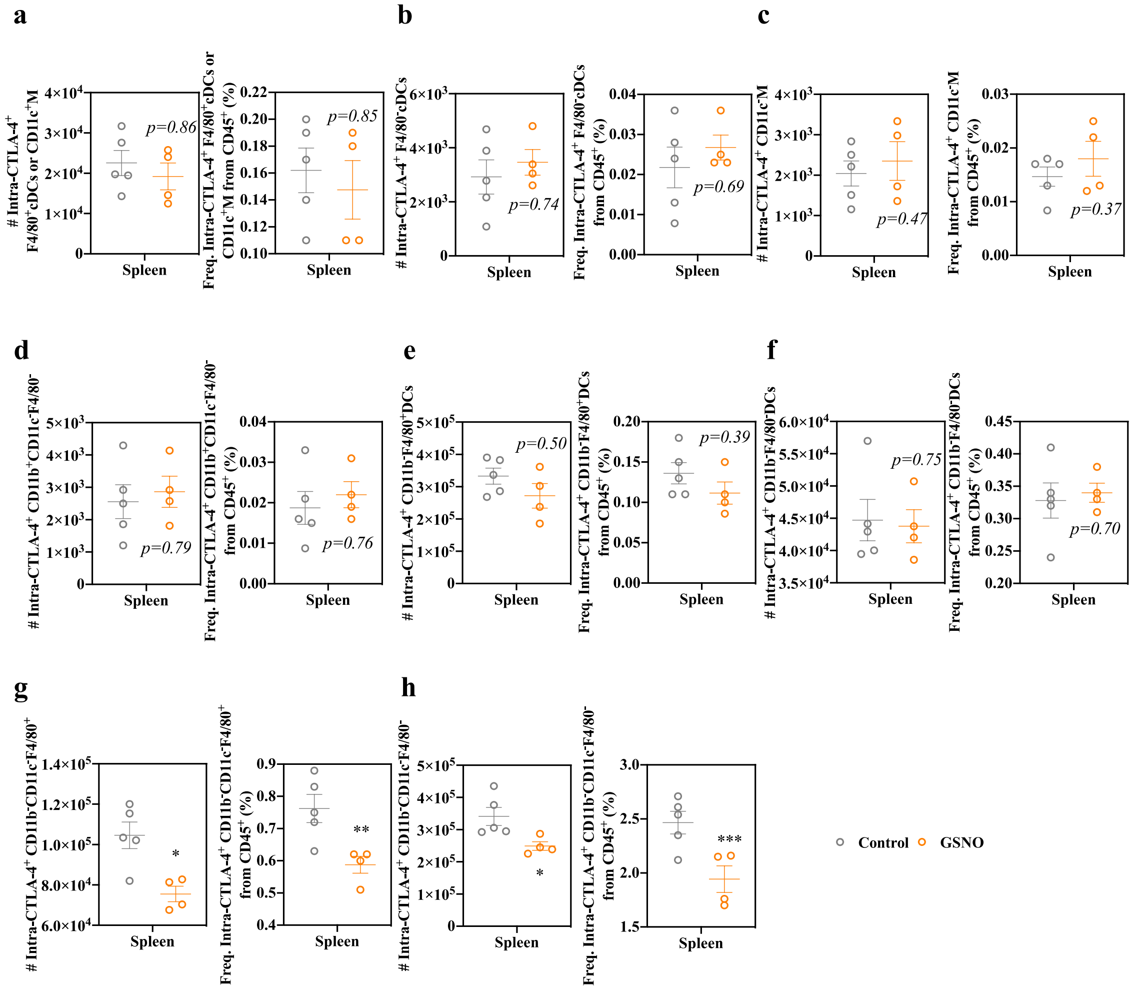

Supplementary Figure 15. Profiles of intracellular CTLA-4 expression of CD11b, CD11c and/or F4/80 expressing immune cells in spleen associated with subcutaneous GSNO treatment. Immune cells in spleen were profiled 1 day after treatment of GSNO (570 µg kg^{-1}) in 30 µL saline. **a-h** Number and percent populations of (**a**) Intra-CTLA-4$^+$ F4/80$^+$cDCs or CD11c$^+$M, (**b**) Intra-CTLA-4$^+$ F4/80$^-$cDCs, (**c**) Intra-CTLA-4$^+$ CD11c$^-$M, (**d**) Intra-CTLA-4$^+$ CD11b$^+$CD11c$^-$F4/80$^-$, (**e**) Intra-CTLA-4$^+$ CD11b$^-$F4/80$^+$DCs, (**f**) Intra-CTLA-4$^+$ CD11b$^-$F4/80$^-$DCs, (**g**) Intra-CTLA-4$^+$ CD11b$^-$CD11c$^-$F4/80$^+$ (left p = 0.0815 and right p = 0.0391), and (**h**) Intra-CTLA-4$^+$ CD11b$^-$CD11c$^-$F4/80$^-$ (left p = 0.0733 and right p = 0.0021). Data are presented as individual biological replicates and mean±SEM. n=5 for control and n=4 for GSNO. *****p < 0.0001, ****p < 0.001, ***p < 0.01, **p < 0.05, and *p < 0.1 with two-tailed Student t-test. Source data are available in a Source Data file.

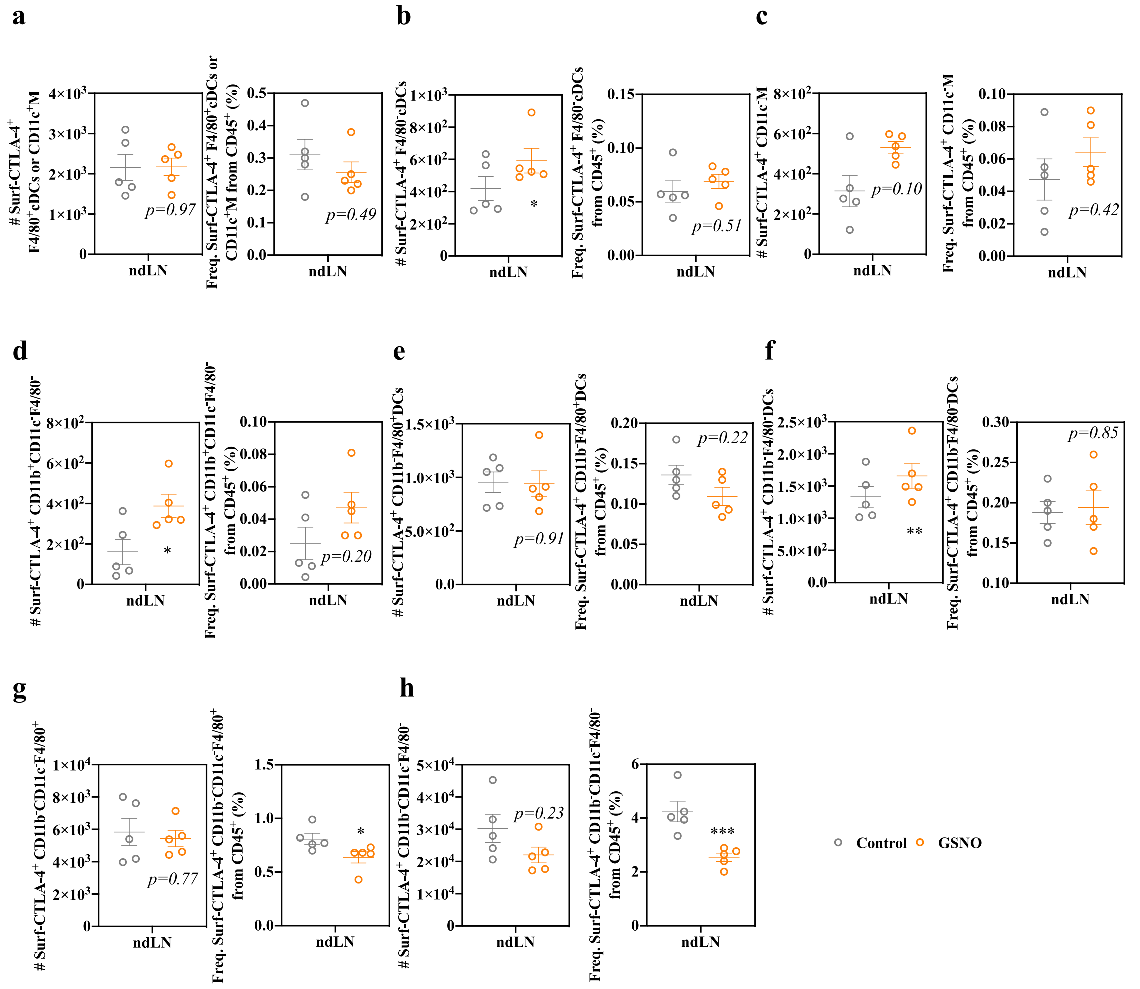

Supplementary Figure 16. Profiles of extracellular CTLA-4 expression of CD11b, CD11c and/or F4/80 expressing immune cells in ndLN associated with subcutaneous GSNO treatment. Immune cells in ndLN were profiled 1 day after treatment of GSNO (570 μg kg^{-1}) in 30 μL saline. **a-h** Number and percent populations of (**a**) Surf-CTLA-4^+ F4/80^+cDCs or CD11c^+M, (**b**) Surf-CTLA-4^+ F4/80^-cDCs (left p = 0.0699), (**c**) Surf-CTLA-4^+ CD11c^-M, (**d**) Surf-CTLA-4^+ CD11b^+CD11c^-F4/80^- (left p = 0.0607), (**e**) Surf-CTLA-4^+ CD11b^-F4/80^+DCs, (**f**) Surf-CTLA-4^+ CD11b^-F4/80^-DCs (left p = 0.0355), (**g**) Surf-CTLA-4^+ CD11b^- CD11c^-F4/80^+ (right p = 0.0630), and (**h**) Surf-CTLA-4^+ CD11b^-CD11c^-F4/80^- (right p = 0.0064). Data are presented as individual biological replicates and mean±SEM. n=5. *****p < 0.0001, ****p < 0.001, ***p < 0.01, **p < 0.05, and *p < 0.1 with two-tailed Student t-test. Source data are available in a Source Data file.

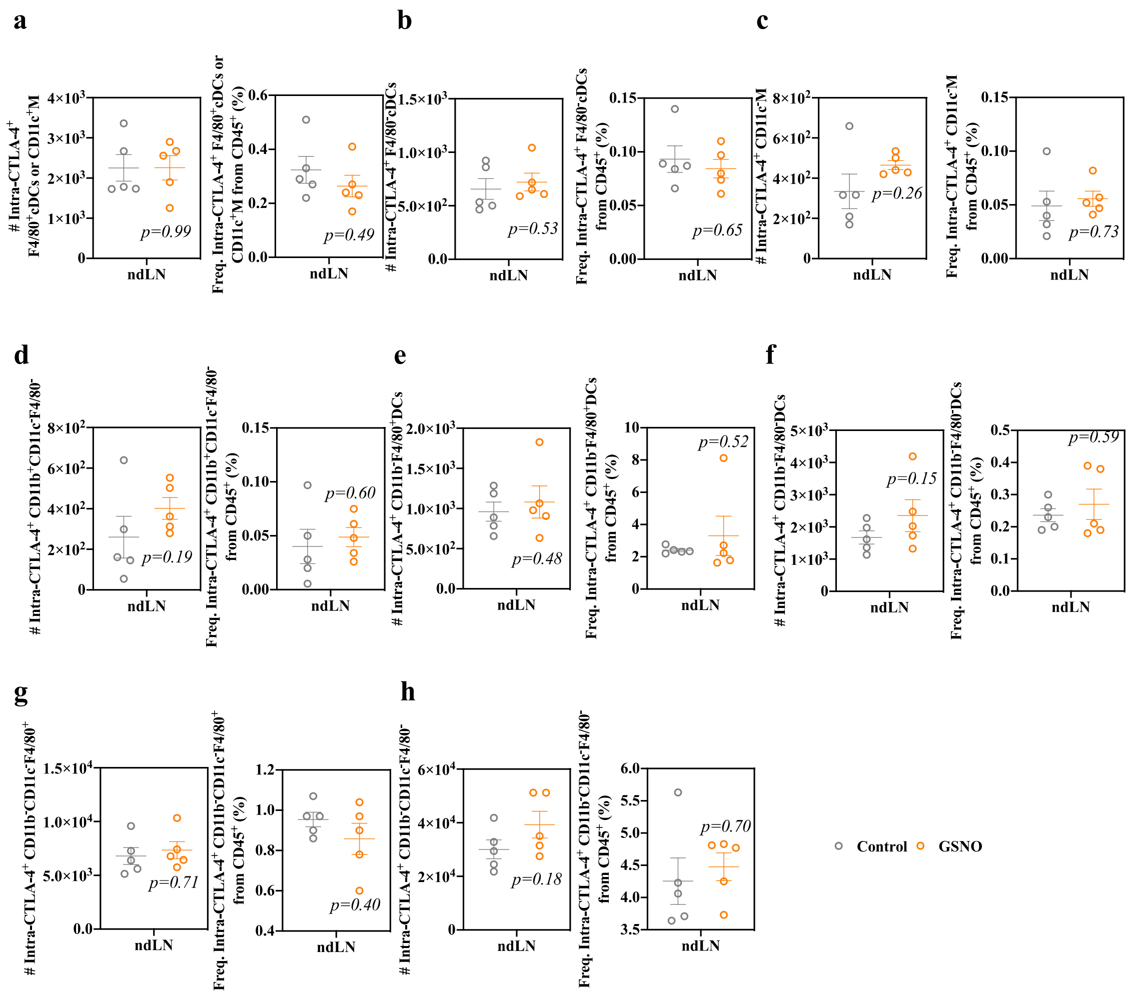

Supplementary Figure 17. Profiles of intracellular CTLA-4 expression of CD11b, CD11c and/or F4/80 expressing immune cells in ndLN associated with subcutaneous GSNO treatment. Immune cells in ndLN were profiled 1 day after treatment of GSNO (570 μg kg^{-1}) in 30 μL saline. **a-h** Number and percent populations of (**a**) Intra-CTLA-4$^+$ F4/80$^+$cDCs or CD11c$^+$M, (**b**) Intra-CTLA-4$^+$ F4/80$^-$cDCs, (**c**) Intra-CTLA-4$^+$ CD11c$^-$M, (**d**) Intra-CTLA-4$^+$ CD11b$^+$CD11c$^-$F4/80$^-$, (**e**) Intra-CTLA-4$^+$ CD11b$^-$F4/80$^+$DCs, (**f**) Intra-CTLA-4$^+$ CD11b$^-$F4/80$^-$DCs, (**g**) Intra-CTLA-4$^+$ CD11b$^-$CD11c$^-$F4/80$^+$, and (**h**) Intra-CTLA-4$^+$ CD11b$^-$CD11c$^-$F4/80$^-$. Data are presented as individual biological replicates and mean±SEM. n=5. *****p < 0.0001, ****p < 0.001, ***p < 0.01, **p < 0.05, and *p < 0.1 with two-tailed Student t-test. Source data are available in a Source Data file.

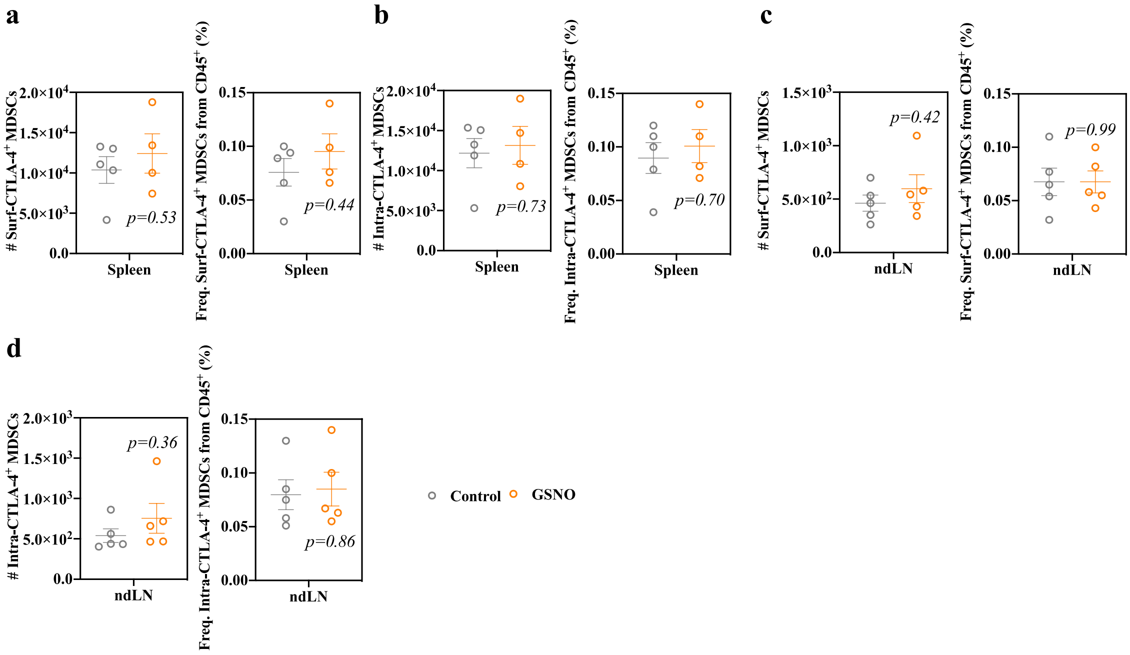

Supplementary Figure 18. Profiles of extracellular and intracellular CTLA-4 expression of MDSCs in spleen and ndLN associated with subcutaneous GSNO treatment. Immune cells in spleen and ndLN were profiled 1 day after treatment of GSNO (570 µg kg^{-1}) in 30 µL saline. **a-d** Number and percent populations of CTLA-4 expressing MDSCs (**a, c**) on extracellularly (Surf-CTLA-4^{+} T_{reg}s) or (**b, d**) intracellularly (Intra-CTLA-4^{+} T_{reg}s) in (**a, b**) spleen and (**c, d**) ndLN. Data are presented as individual biological replicates and mean±SEM. **a, b** n=5 for control and n=4 for GSNO. **c, d** n=5. *****$p < 0.0001$, ****$p < 0.001$, ***$p < 0.01$, **$p < 0.05$, and *$p < 0.1$ with two-tailed Student t-test. Source data are available in a Source Data file.

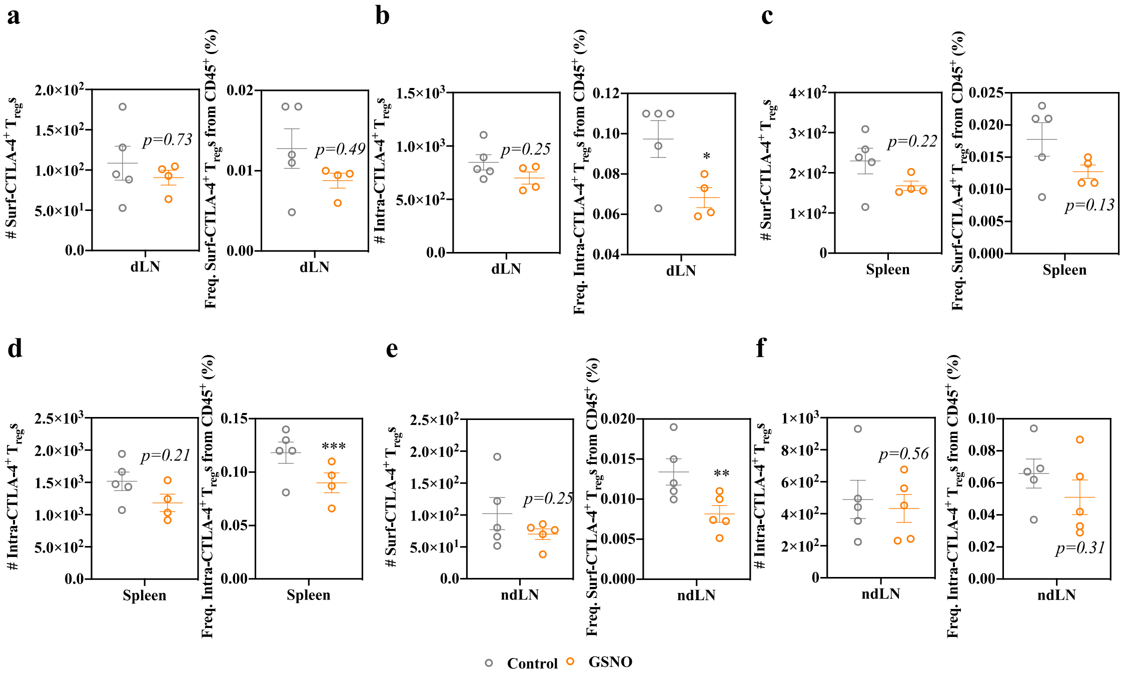

Supplementary Figure 19. Profiles of extracellular and intracellular CTLA-4 expression of T_{reg}s in dLN, spleen, and ndLN associated with subcutaneous GSNO treatment. Immune cells in dLN, spleen, and ndLN were profiled 1 day after treatment of GSNO (570 µg kg^{-1}) in 30 µL saline. **a-f** Number and percent populations of CTLA-4 expressing T_{reg}s (**a, c, e**) on extracellularly (Surf-CTLA-4$^+$ T_{reg}s) or (**b, d, f**) intracellularly (Intra-CTLA-4$^+$ T_{reg}s) in (**a, b**) dLN (**b** right $p = 0.0835$), (**c, d**) spleen (**d** right $p = 0.0076$), and (**e,f**) ndLN (**e** right $p = 0.0363$). Data are presented as individual biological replicates and mean±SEM. **a-d** n=5 for control and n=4 for GSNO. **e, f** n=5. *****$p < 0.0001$, ****$p < 0.001$, ***$p < 0.01$, **$p < 0.05$, and *$p < 0.1$ with two-tailed Student t-test. Source data are available in a Source Data file.

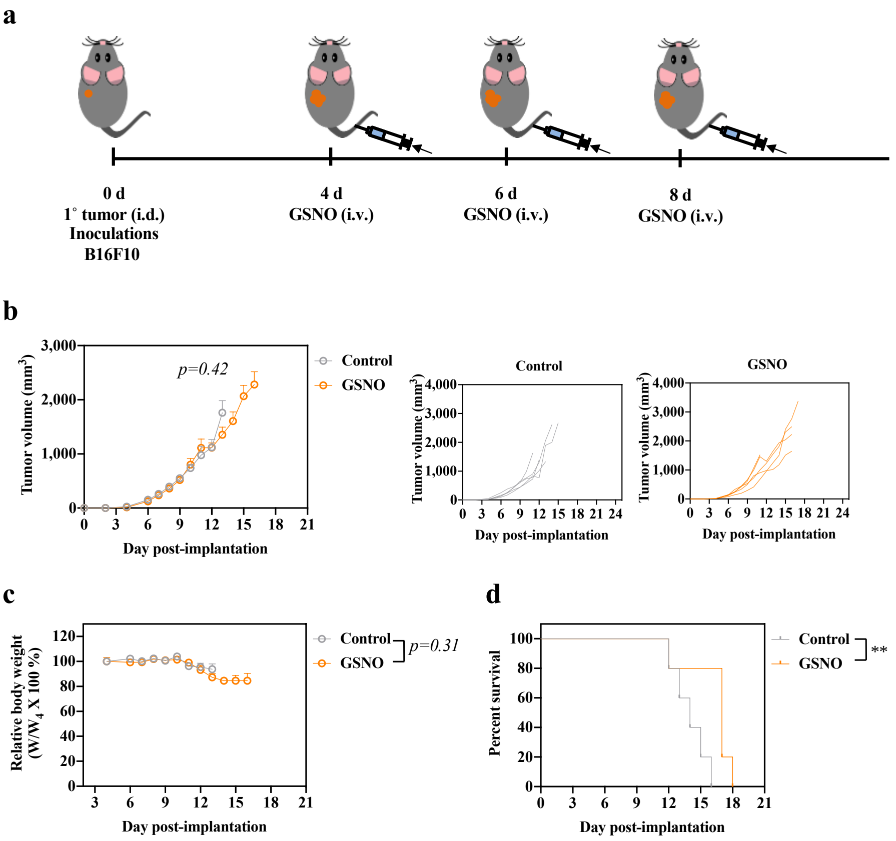

Supplementary Figure 20. In vivo effects of intravenously administered GSNO on tumor progression. **a** Tumor model and treatment schedule. Tumors were formed in C57Bl/6 mice by inoculation of 10^5 B16F10-OVA cells in 30 µL saline on day 0. GSNO (600 µg kg^{-1}) in 30 µL saline was administered i.v. on day 4, 6, and 8. **b** Tumor volume. **c** Relative body weight changes poste treatment. **d** Kaplan–Meier survival curves during treatment. Data are presented as individual biological replicates and mean±SEM. n=5. *****p < 0.0001, ****p < 0.001, ***p < 0.01, **p < 0.05, and *p < 0.1. **b** ANOVA using linear mixed-effects model. **c** Two-way ANOVA using Tukey post-hoc statistical hypothesis. **d** Log-rank using Mantel-Cox statistical hypothesis. Source data are available in a Source Data file.

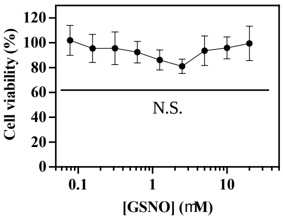

Supplementary Figure 21. Alamar Blue assay of B16F10-OVA cells treated with GSNO. 10^3 B16F10-OVA cells in 96 well cell culture plate was incubated for 2 days treated with GSNO, followed by recording fluorescence (560 nm excitation, 590 nm emission) with Synergy H4 microplate reader after 1 h incubation with AlamarBlue™ cell viability reagent. Data are presented as mean±SD. n=4 for 0 and 20 μM, and n=5 for the other. *****$p < 0.0001$, ****$p < 0.001$, ***$p < 0.01$, **$p < 0.05$, and *$p < 0.1$ with one-way ANOVA using Tukey post-hoc statistical hypothesis by comparing with 0 mg mL^{-1}. Source data are available in a Source Data file.

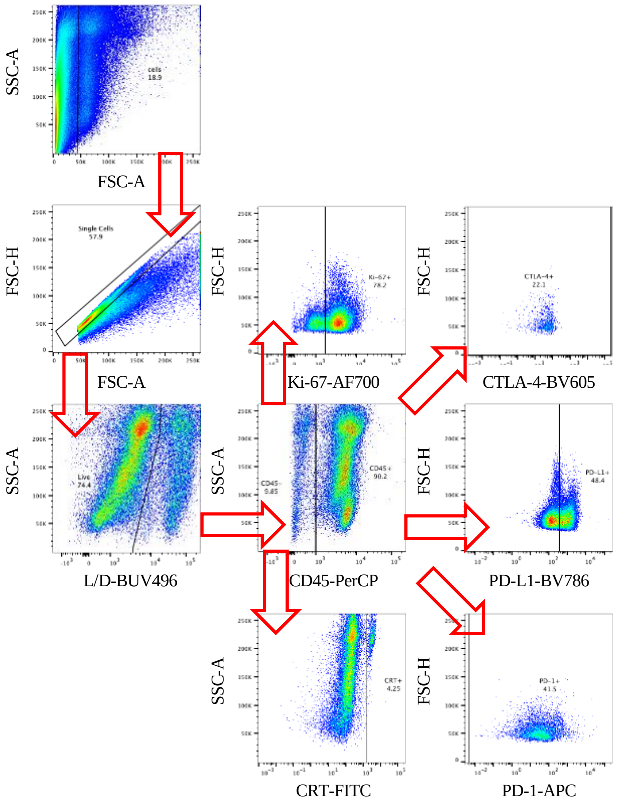

Supplementary Figure 22. Gating strategy to investigate the immunogenicity of tumor cells from GSNO-treated primary tumors bearing two B16F10-OVA tumors. 1º and 2º tumors (10^5 B16F10-OVA cells in 30 μL saline) were inoculated on day 0 and day 4, respectively. GSNO (570 μg kg^{-1}) in 30 μL saline was administered on day 7. Tumor cells were profiled 1 day after treatment of GSNO. LSR Fortessa flow cytometry and FlowJo were employed to analyze and profiles the stained cells.

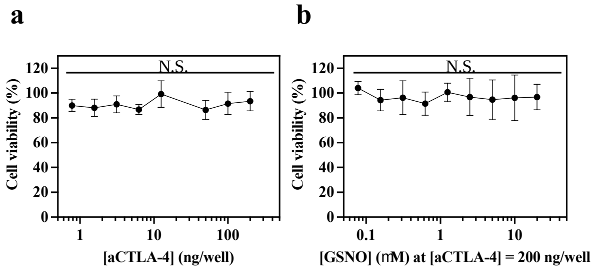

Supplementary Figure 23. AlamarBlue assessment of B16F0-OVA cell viability in response to in vitro treatment with aCTLA-4 and GSNO+aCTLA-4. a, b 10^3 B16F10-OVA cells in 96 well cell culture plates incubated with (**a**) aCTLA-4 and (**b**) GSNO+aCTLA-4 for 2 days, followed by recording fluorescence (560 nm excitation, 590 nm emission) with Synergy H4 microplate reader after 1 h incubation with AlamarBlue™ cell viability reagent. Data are presented as mean±SD. **a** n=4 for 12.5 ng/well, and n=5 for the other. **b** n=3 for 0.08, 1.25 and 5 µM, n=4 for 0 and 10 µM, and n=5 for the other. *****$p < 0.0001$, ****$p < 0.001$, ***$p < 0.01$, **$p < 0.05$, and *$p < 0.1$ with one-way ANOVA using Tukey post-hoc statistical hypothesis by comparing with 0 mg mL^{-1}. Source data are available in a Source Data file.

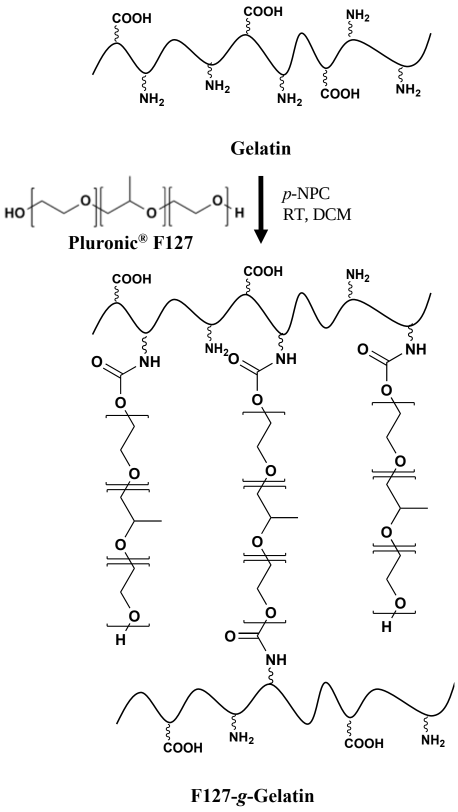

Supplementary Figure 24. Synthetic scheme of F127-*g*-Gelatin. F127 was activated with 4-nitrophenyl chloroformate (*p*-NPC) overnight. *p*-NPC activated F127 yielded via precipitation was reacted with gelatin type A (300g bloom) overnight. F127-*g*-Gelatin was yielded by dialyzing against deionized water with dialysis tube (MWCO 100 KDa) and freezing drying.

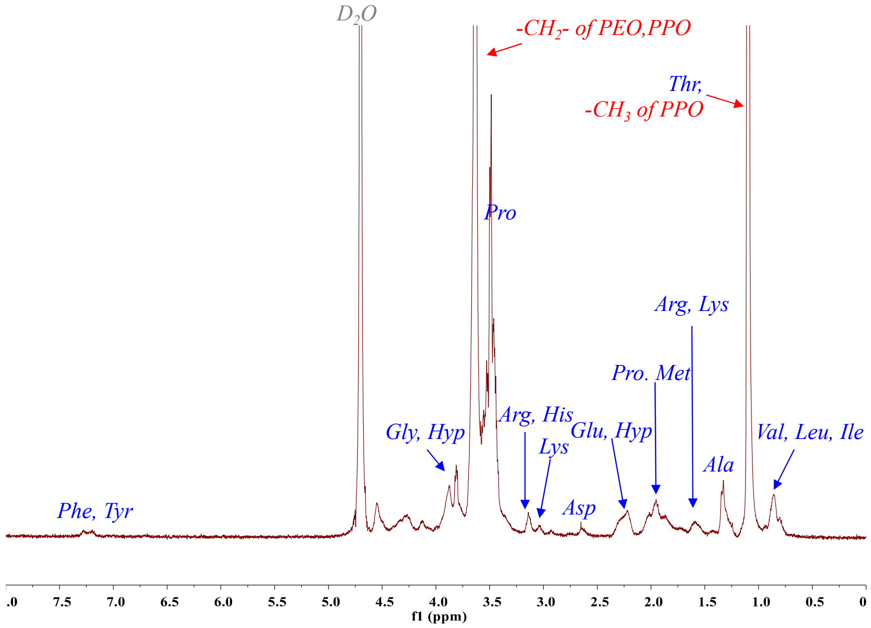

Supplementary Figure 25. **^{1}H NMR of F127-*g*-Gelatin.** Peaks for F127 and gelatin were marked with red and blue colors, respectively.

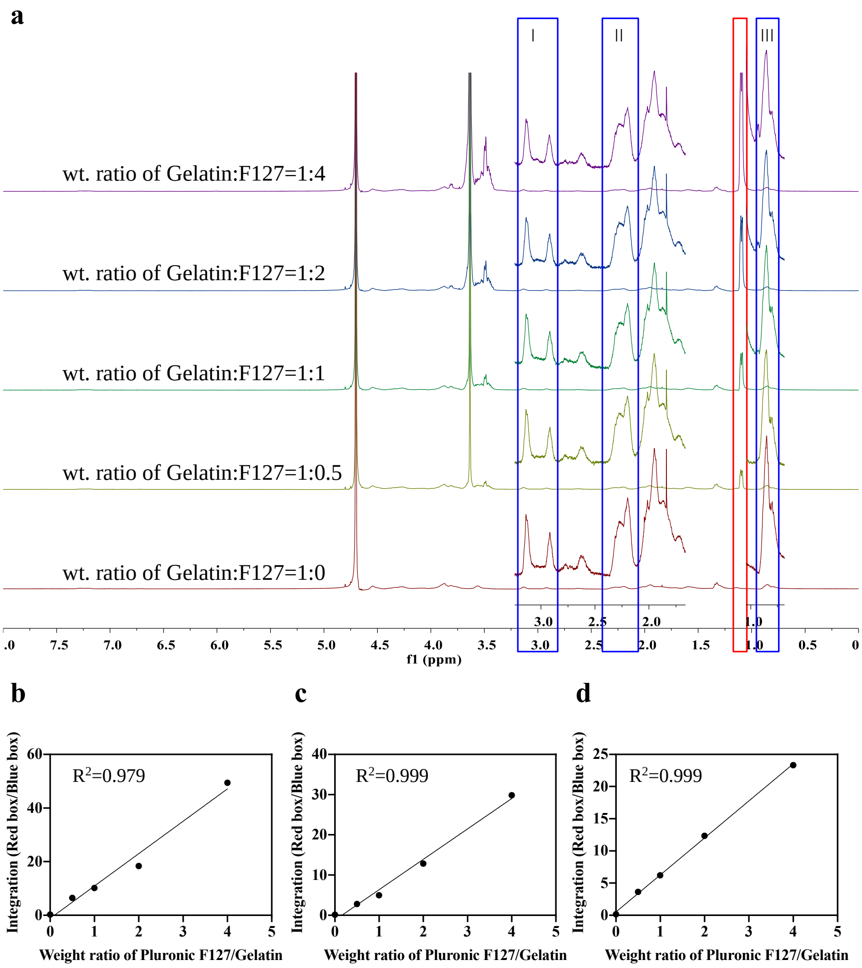

Supplementary Figure 26. Quantitative ^{1}H NMR analysis of F127-*g*-Gelatin. a Gelatin and F127 were physically mixed at a predeterminded weight ratio, and the F127 and gelatin were marked with red and blue colors, respectively. Blue box I represents the Arg and Lys peaks of gelatin. Blue box II represents the Glu and Hyp peaks of gelatin. Blue box III represents the Val, Leu, and Ile peaks of gelatin. Red box represents the methyl groups of polypropylene oxide in F127. **b-d** Integration ratio of Red box to each blue box were plotted with different weight ratio of Gelatin and F127. The composition of F127-*g*-Gelatin was calculated by using each respective standard curve (**b-d**), and results averaged. As a result, wt. ratio of Gelatin:F127 was confirmed to 1:1.85±0.16, which was converted percentage ratio to be 35.1 wt.%:64.9±1.97 wt.%. Source data are available in a Source Data file.

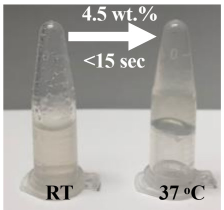

Supplementary Figure 27. Image of F127-*g*-Gelatin thermosensitive hydrogel at 4.5 wt.% at RT (left) and 37 ˚C (right).

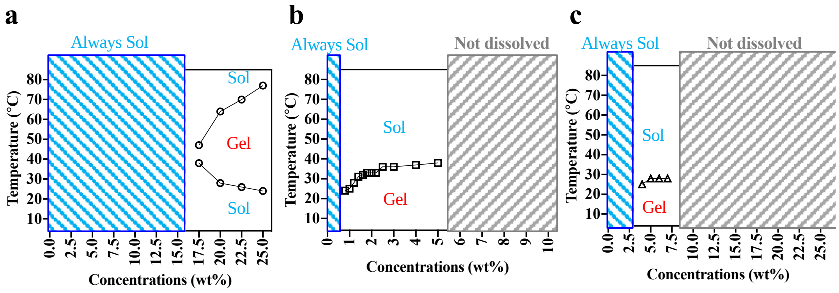

Supplementary Figure 28. Concentration-dependent sol-gel transition properties of control groups for F127-*g*-Gelatin. a-c Sol-gel transition graphs of (**a**) F127, (**b**) gelatin, and (**c**) mixture of gelatin and F127 (blend mass ratio for Gelatin:F127=35.1:64.9).

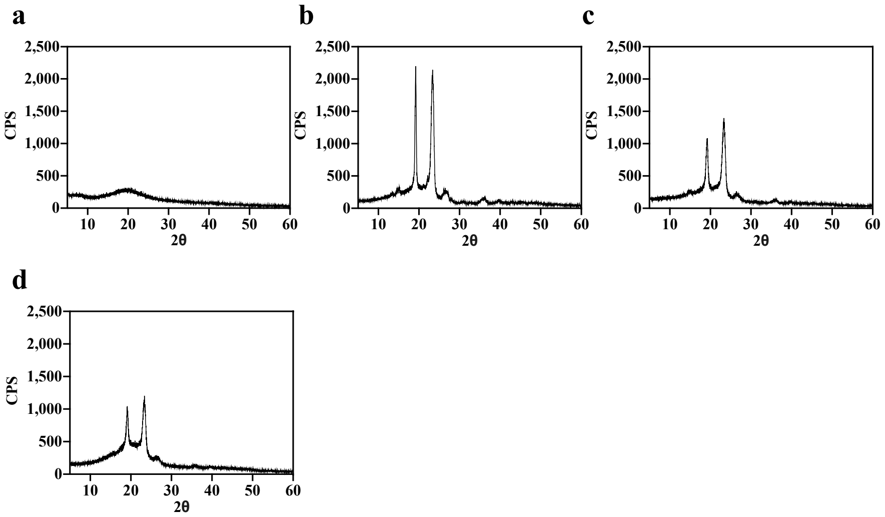

Supplementary Figure 29. XRD patterns of F127-*g*-Gelatin to investigate the powder crystallinity. a-d XRD curves of (**a**) gelatin, (**b**) F127, (**c**) mixture of gelatin and F127 (blend mass ratio for Gelatin:F127=35.1:64.9), and (**d**) F127-*g*-Gelatin. All polymer samples were prepared by lyophilizing after

incubation of 4 wt.% polymer solutions at 37 °C for 1 h and ultra-rapidly freezing using liquid nitrogen. Source data are available in a Source Data file.

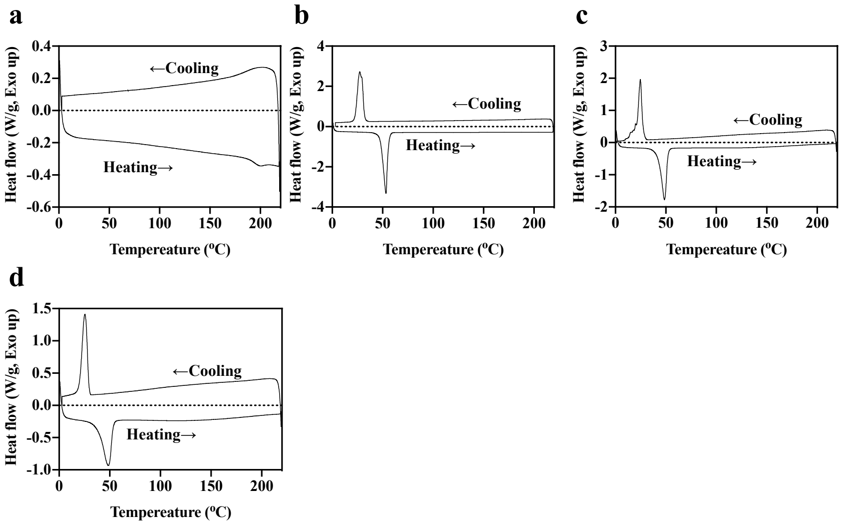

Supplementary Figure 30. Powder DSC thermograms of F127-*g*-Gelatin to investigate the powder crystallinity. a-d DSC curves of (**a**) gelatin (T_c=N.D., and T_m=N.D.), (**b**) F127 (T_c=27.2 ˚C, and T_m=53.3 ˚C), (**c**) mixture of Gelatin and F127 (blend mass ratio for Gelatin:F127=35.1:64.9) (T_c=24.7 ˚C, and T_m=48.6 ˚C), and (**d**) F127-*g*-Gelatin (T_c=25.5 ˚C, and T_m=48.7 ˚C). All polymer samples were prepared by lyophilizing after incubation of 4 wt.% polymer solutions at 37 °C for 1 h and ultra-rapid freezing using liquid nitrogen. T_c and T_m represents the crystalline temperature and melting temperature, respectively. N.D. means "not determined". Source data are available in a Source Data file.

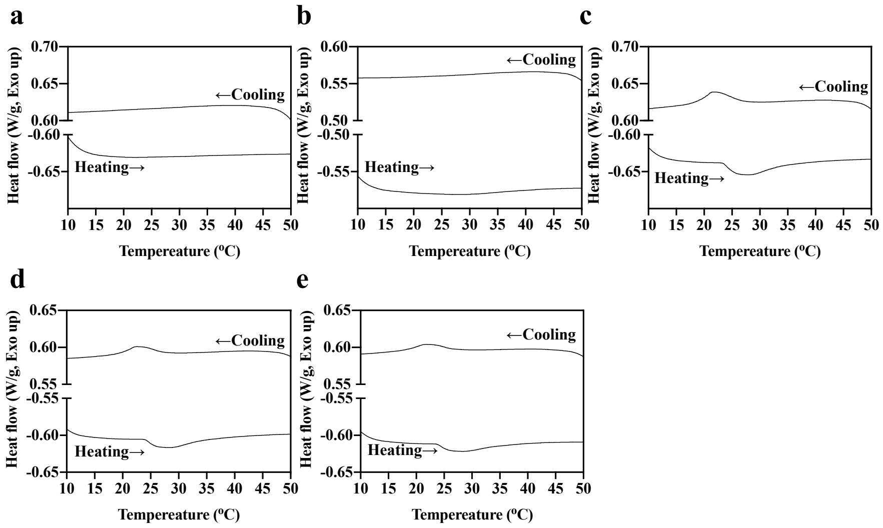

Supplementary Figure 31. Solution DSC thermograms of F127-*g*-Gelatin. a-e DSC curves of solutions containing (**a**) deionized water, (**b**) gelatin (4 wt.%), (**c**) F127 (4 wt.%), (**d**) mixture of gelatin and F127 (Final 4 wt.%, blend mass ratio for Gelatin:F127=35.1:64.9), and (**e**) F127-*g*-Gelatin (4 wt.%). Source data are available in a Source Data file.

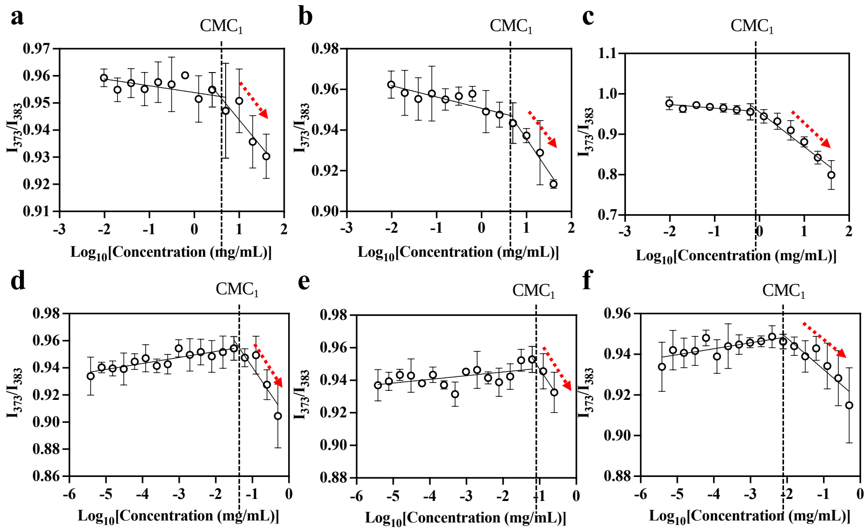

Supplementary Figure 32. CMC measurement of F127-*g*-Gelatin with typical pyrene method. a-f Polymer concentration-dependent ratiometric emitted fluorescence (373 nm and 383 nm) of pyrenes at an excitation wavelength of 336 nm was recorded. The intersection of two distinctive linear lines represents CMC_1. **a-f** Data represent measured CMC_1 of (**a**) F127 at room temperature (RT), (**b**) mixture of gelatin and F127 (blend mass ratio for Gelatin:F127=35.1:64.9) at RT, (**c**) F127-*g*-Gelatin at RT, (**d**) F127 at 37 ˚C, (**e**)

mixture of gelatin and F127 (blend mass ratio for Gelatin:F127=35.1:64.9) at 37 ˚C, and (**f**) F127-*g*-Gelatin at 37 ˚C. Gelatin does not show any distinctive intersections. Data are presented as mean±SD. n=3 except (**d**) n=5. Source data are available in a Source Data file.

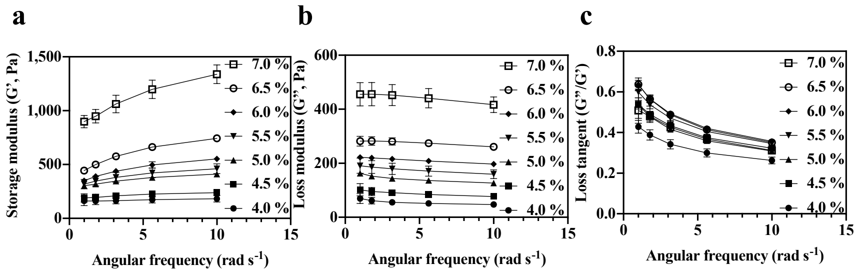

Supplementary Figure 33. Rheology of F127-*g*-Gelatin at different concentrations at 37 ˚C. a-c (**a**) Storage modulus (G'), (**b**) loss modulus (G"), and (**c**) loss tangent (G"/G') of F127-*g*-Gelatin at 4.0-7.0 wt.%. Data are presented as mean±SD. n=3 for 4.5 wt.%, n=4 for 5.5, 6.0, 6.5, and 7.0 wt.%, and n=5 for 4.0 and 5.0 wt.%. Source data are available in a Source Data file.

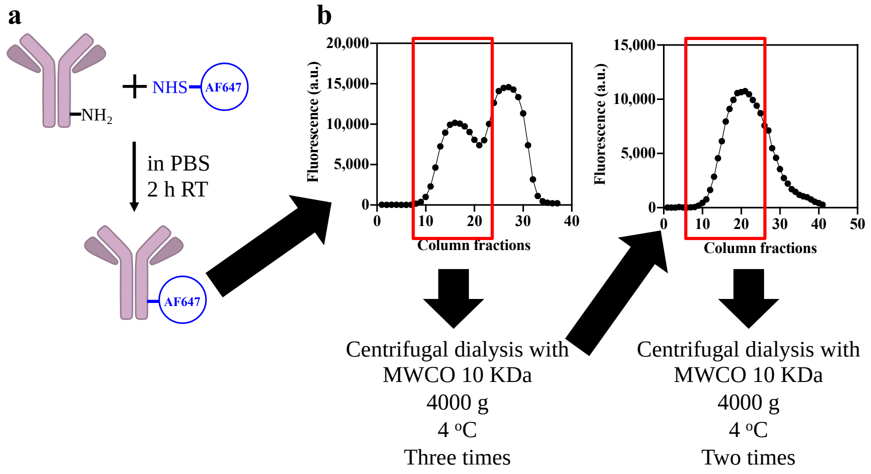

Supplementary Figure 34. Synthesis and preparation of Alexa Fluor™ 647 labeled aCTLA-4. a-b (**a**) aCTLA-4 in PBS was reacted with Alexa Fluor™ 647 NHS Ester (AF647-NHS) in DMSO at room temperature, (**b**) followed by purification with CL-6B Sepharose® column and spin filter with Amicon® Ultra centrifugal filter (Milipore, MWCO 10 kDa). Source data are available in a Source Data file.

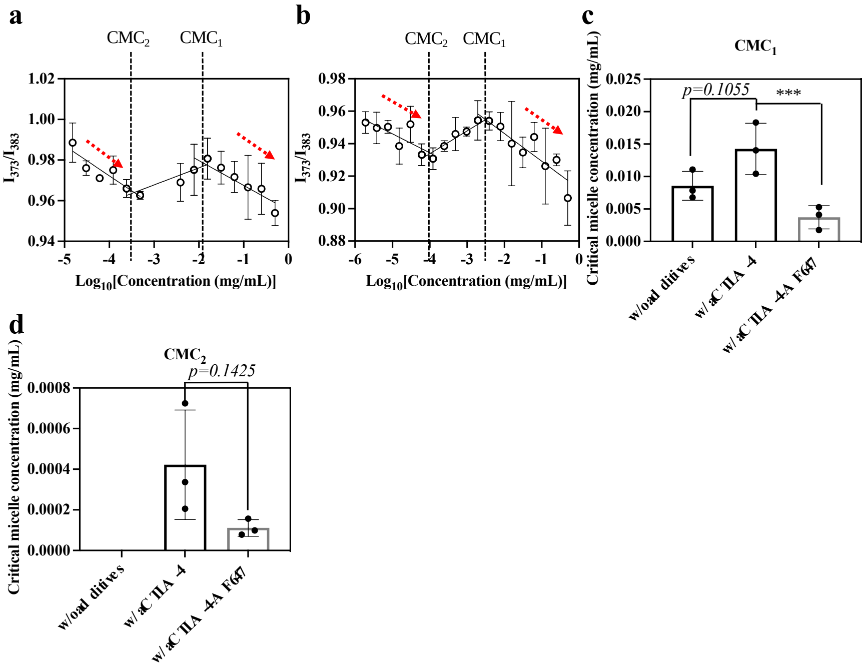

Supplementary Figure 35. CMC measurement of F127-*g*-Gelatin containing aCTLA-4 and aCTLA-AF647 with typical pyrene method. a, b Polymer concentration-dependent ratiometric emitted fluorescence (373 nm and 383 nm) of pyrenes at excitation wavelength of 336 nm was recorded with or without aCTLA-4 or aCTLA-4AF647. The intersections of two distinctive linear lines represent CMC_1 and CMC_2. CMC_1 and CMC_2 of (**a**) F127-*g*-Gelatin containing aCTLA at 37 ˚C, and (**b**) F127-*g*-Gelatin containing aCTLA-AF647 at 37 ˚C. **c, d** Quantitative and statistical analysis of (**c**) CMC_1 ($p = 0.1687$ for w/o additives vs. w/ aCTLA-4-AF647, and $p = 0.0090$ for w/ aCTLA-4 vs. w/ aCTLA-4-AF647) and (**d**) CMC_2 of F127-*g*-Gelatin containing aCTLA-4 and aCTLA-AF647 at 37 ˚C. Data are presented as mean±SD. n=3. *****$p < 0.0001$, ****$p < 0.001$, ***$p < 0.01$, **$p < 0.05$, and *$p < 0.1$. **c** One-way ANOVA using Tukey post-hoc statistical hypothesis. **d** Two-tailed Student t-test. Source data are available in a Source Data file.

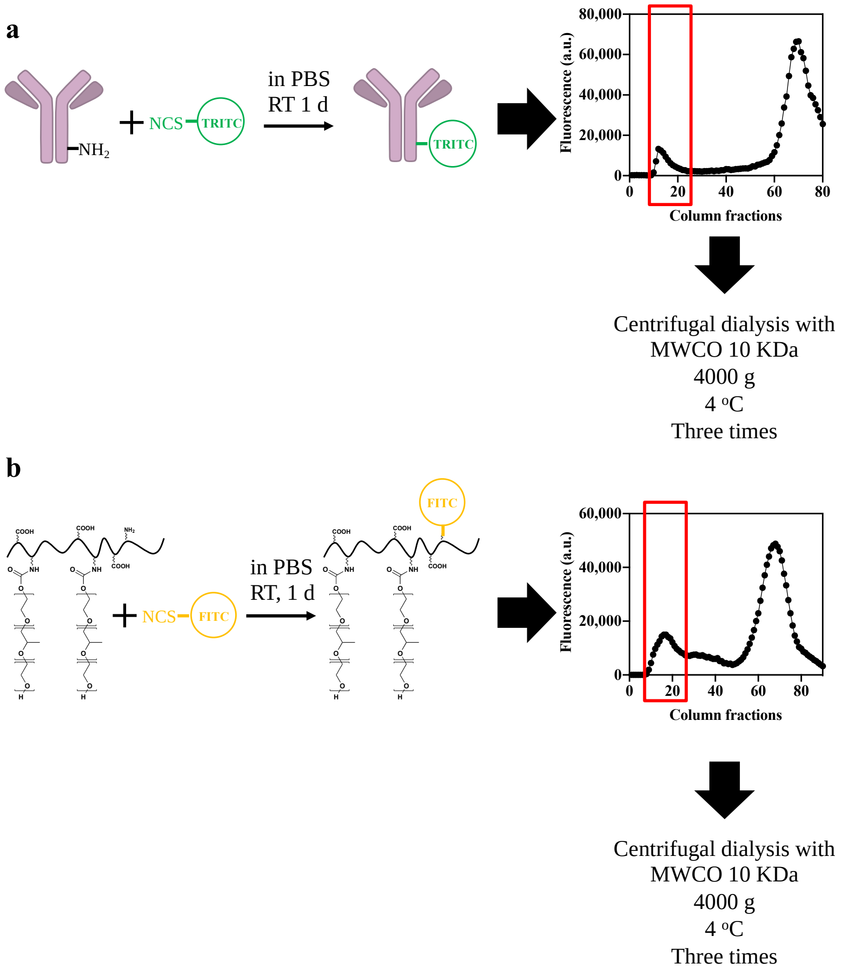

Supplementary Figure 36. Synthesis and preparation of TRITC labeled aCTLA-4 and FITC labeled F127-*g*-Gelatin. a aCTLA-4 in PBS was reacted with TRITC in DMSO at room temperature, followed by purification with CL-6B Sepharose® column and spin filter with Amicon® Ultra centrifugal filter (Milipore, MWCO 10 kDa). **b** F127-*g*-Gelatin in PBS was reacted with FITC in DMSO at room temperature, followed by purification with CL-6B Sepharose® column and spin filter with Amicon® Ultra centrifugal filter (Milipore, MWCO 10 kDa). Source data are available in a Source Data file.

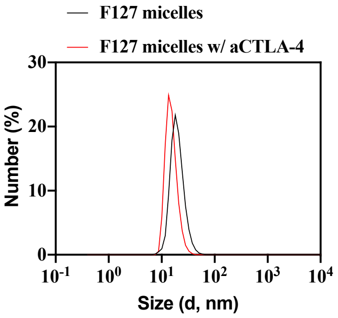

Supplementary Figure 37. Size distribution of F127 micelles with and without aCTLA-4 (n=12). Final concentrations of F127 and aCTLA-4 concentrations are 0.9 wt.% and 0.542 mg mL^{-1}, respectively, which are equivalent to the concentrations of F127-*g*-Gelatin and aCTLA-4 in Figure 4H. n=12. Source data are available in a Source Data file.

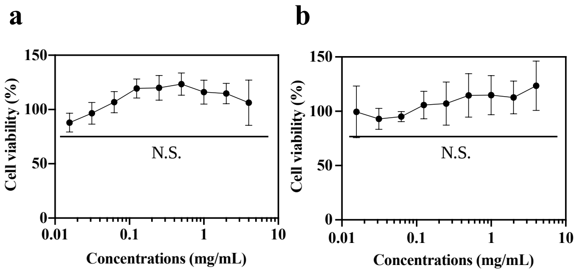

Supplementary Figure 38. AlamarBlue assay of NIH3T3 and B16F10-OVA treated with F127-*g*-Gelatin. a, b 10^4 (**a**) NIH3T3 or (**b**) B16F10-OVA cells in 96 well cell culture plate were each treated with F127-*g*-Gelatin for 2 days, followed by recording fluorescence (560 nm excitation, 590 nm emission) with Synergy H4 microplate reader after 1 h incubation with AlamarBlue™ cell viability reagent. Data are presented as mean±SD. **a** n=8. **b** n=4. *****$p < 0.0001$, ****$p < 0.001$, ***$p < 0.01$, **$p < 0.05$, and *$p < 0.1$ with one-way ANOVA using Tukey post-hoc statistical hypothesis by comparing it with 0 mg mL^{-1}. Source data are available in a Source Data file.

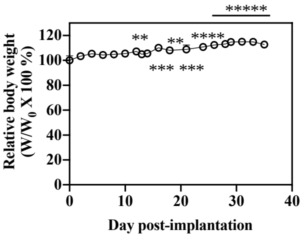

Supplementary Figure 39. Relative body weight changes after treatment of bare hydrogel. Relative body weight was measured after one-time subcutaneous administration of bare F127-*g*-Gelatin hydrogel on tumor-free mice. Data are presented as mean±SEM. n=5. *****$p < 0.0001$, ****$p < 0.001$, ***$p < 0.01$, **$p < 0.05$, and *$p < 0.1$ with one-way ANOVA using Tukey post-hoc statistical hypothesis by comparing it with day 0 ($p = 0.0468$ for day 12, $p = 0.0011$ for day 16, $p = 0.0187$ for day 18, $p = 0.0070$ for day 21, $p = 0.0005$ for day 24, and $p < 0.0001$ for day 26, 28, 29, 31, 33, and 35). Source data are available in a Source Data file.

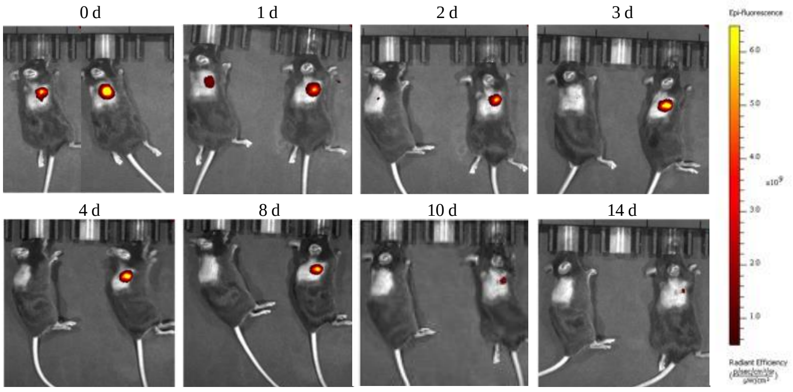

Supplementary Figure 40. IVIS® images of mice treated with Alexa Fluor™ 647 labeled aCTLA-4 by bolus delivery or with F127-*g*-Gelatin. Representative time-resolved IVIS® images of aCTLA-4-AF647 fluorescence were used to quantify the amounts of aCTLA-4 not diffused from the injection sites. Their inversed values represent the amounts of aCTLA-4 released from the injection site as an injected bolus or F127-*g*-Gelatin hydrogel. The quantified fluorescence values are reported in Figure 4**n**.

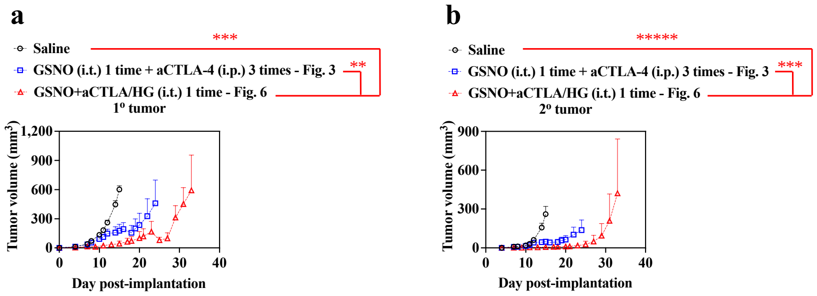

Supplementary Figure 41. Comparison of GSNO+aCTLA-4/HG (i.t., 1 time) in Fig. 6 and GSNO (i.t., 1 time)+aCTLA-4 (i.p., 3 times) in Figure 3. a 1° (directly injected) tumor size. **b** 2° (uninjected) tumor size. Data are presented as mean±SEM. n=5. *****$p < 0.0001$, ****$p < 0.001$, ***$p < 0.01$, **$p < 0.05$, and *$p < 0.1$ with ANOVA using linear mixed-effects model. Source data are available in a Source Data file.

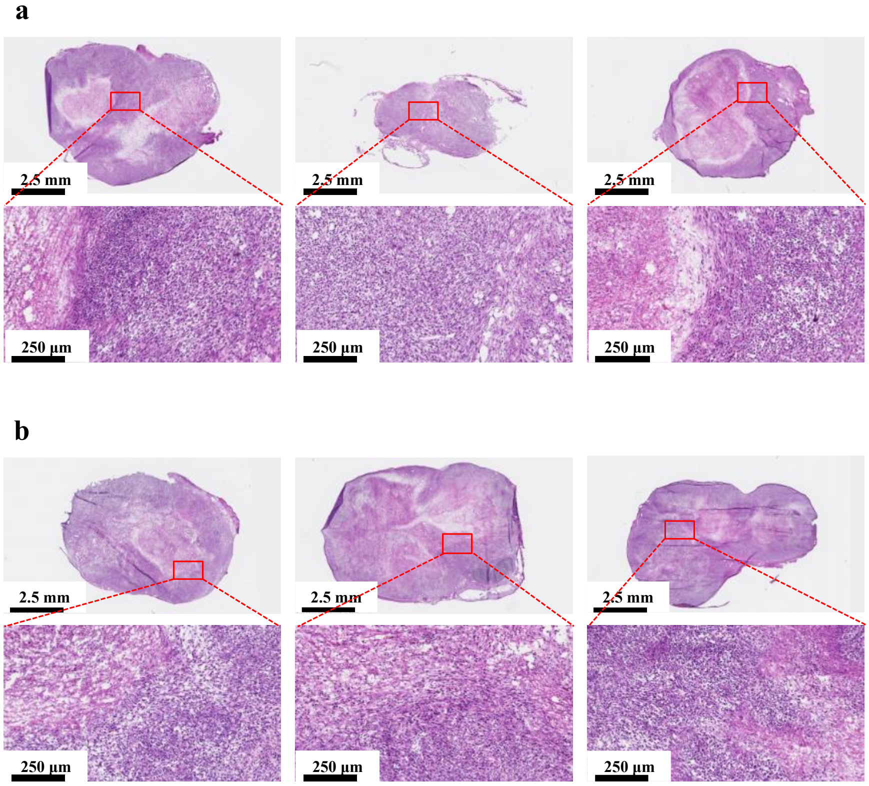

Supplementary Figure 42. Representative histological H&E images of 4T1 tumor tissues treated with F127-*g*-Gelatin hydrogels. a, b 1° and 2° tumors were formed in Balb/C mice by inoculation of 3X10^{5} 4T1 cells in 30 μL saline to the left mammary fatpad on day 0 and right mammary fatpad on day 4, respectively. 30 μL of (**a**) saline and (**b**) 4.5 wt.% F127-*g*-Gelatin hydrogel were administered intratumorally on day 7. 1° tumor tissues were dissected, frozen with OCT, and stained with H&E after sacrificing mice on day 14 (n=3).

Supplementary Table 1. Statistical comparison for Fig. 3b, c with ANOVA using linear mixed-effects model.

vs.	***P* value**	
	1° tumor	2° tumor
Control vs. Control+aCTLA-4	0.8650	0.9990
Control vs. GSNO	0.9410	0.8561
Control vs. GSNO+aCTLA-4	0.0032	<0.0001
Control+aCTLA-4 vs. GSNO	0.6135	0.8394
Control+aCTLA-4 vs. GSNO+aCTLA-4	0.0536	0.0001
GSNO vs. GSNO+aCTLA-4	0.0015	0.0116

Supplementary Table 2. Statistical comparison for Fig. 3e with Log-rank analysis with Mantel-Cox statistical hypothesis and Fig. 3f-i with one-way ANOVA using Tukey post-hoc statistical hypothesis.

**Figures**	***vs.***	***P* value**
**e**	Control vs. Control+aCTLA-4	0.5509
	Control vs. GSNO	0.4013
	Control vs. GSNO+aCTLA-4	0.1189
	Control+aCTLA-4 vs. GSNO	0.1290
	Control+aCTLA-4 vs. GSNO+aCTLA-4	0.1276
	GSNO vs. GSNO+aCTLA-4	0.0939
**f** **$CD45^+$**	Control vs. Control+aCTLA-4	>0.9999
	Control vs. GSNO	0.9988
	Control vs. GSNO+aCTLA-4	0.0214
	Control+aCTLA-4 vs. GSNO	0.9992
	Control+aCTLA-4 vs. GSNO+aCTLA-4	0.0221
	GSNO vs. GSNO+aCTLA-4	0.0409
**g** **$CD4^+$ T**	Control vs. Control+aCTLA-4	0.9997
	Control vs. GSNO	>0.9999
	Control vs. GSNO+aCTLA-4	0.1518
	Control+aCTLA-4 vs. GSNO	0.9988
	Control+aCTLA-4 vs. GSNO+aCTLA-4	0.1772
	GSNO vs. GSNO+aCTLA-4	0.1706
**g** **LAG-3^+ $CD4^+$ T**	Control vs. Control+aCTLA-4	0.9997
	Control vs. GSNO	0.9997

	Control vs. GSNO+aCTLA-4	0.0169
	Control+aCTLA-4 vs. GSNO	0.9980
	Control+aCTLA-4 vs. GSNO+aCTLA-4	0.0142
	GSNO vs. GSNO+aCTLA-4	0.0295
**g** **PD-1⁺ CD4⁺ T**	Control vs. Control+aCTLA-4	0.9980
	Control vs. GSNO	0.9996
	Control vs. GSNO+aCTLA-4	0.0725
	Control+aCTLA-4 vs. GSNO	0.9930
	Control+aCTLA-4 vs. GSNO+aCTLA-4	0.0995
	GSNO vs. GSNO+aCTLA-4	0.0797
**g** **T_{reg}**	Free aCTLA-4 vs. aCTLA-4 micelle	0.9980
	Free aCTLA-4 vs. aCTLA-4 hydrogel	0.9914
	aCTLA-4 micelle vs. aCTLA-4 hydrogel	0.2146
	Free aCTLA-4 vs. aCTLA-4 micelle	0.9993
	Free aCTLA-4 vs. aCTLA-4 hydrogel	0.2803
	aCTLA-4 micelle vs. aCTLA-4 hydrogel	0.3852
**h** **$CD8^+$ T**	Free aCTLA-4 vs. aCTLA-4 micelle	0.9934
	Free aCTLA-4 vs. aCTLA-4 hydrogel	>0.9999
	aCTLA-4 micelle vs. aCTLA-4 hydrogel	0.0593
	Free aCTLA-4 vs. aCTLA-4 micelle	0.9945
	Free aCTLA-4 vs. aCTLA-4 hydrogel	0.0362
	aCTLA-4 micelle vs. aCTLA-4 hydrogel	0.0794
**h** **$CD25^+$ $CD8^+$ T**	Free aCTLA-4 vs. aCTLA-4 micelle	0.9942
	Free aCTLA-4 vs. aCTLA-4 hydrogel	>0.9999
	aCTLA-4 micelle vs. aCTLA-4 hydrogel	0.0644
	Free aCTLA-4 vs. aCTLA-4 micelle	0.9938
	Free aCTLA-4 vs. aCTLA-4 hydrogel	0.0402
	aCTLA-4 micelle vs. aCTLA-4 hydrogel	0.0889
**h** **LAG-3^+ $CD8^+$ T**	Free aCTLA-4 vs. aCTLA-4 micelle	0.9896
	Free aCTLA-4 vs. aCTLA-4 hydrogel	>0.9999
	aCTLA-4 micelle vs. aCTLA-4 hydrogel	0.0813
	Free aCTLA-4 vs. aCTLA-4 micelle	0.9878
	Free aCTLA-4 vs. aCTLA-4 hydrogel	0.0462
	aCTLA-4 micelle vs. aCTLA-4 hydrogel	0.1130
**h**	Free aCTLA-4 vs. aCTLA-4 micelle	0.9932

**PD-1⁺ CD8⁺ T**	Free aCTLA-4 vs. aCTLA-4 hydrogel	0.9981
	aCTLA-4 micelle vs. aCTLA-4 hydrogel	0.0151
	Free aCTLA-4 vs. aCTLA-4 micelle	0.9739
	Free aCTLA-4 vs. aCTLA-4 hydrogel	0.0254
	aCTLA-4 micelle vs. aCTLA-4 hydrogel	0.0160
**h Tetramer⁺ CD8⁺ T**	Free aCTLA-4 vs. aCTLA-4 micelle	0.9451
	Free aCTLA-4 vs. aCTLA-4 hydrogel	0.9946
	aCTLA-4 micelle vs. aCTLA-4 hydrogel	0.0263
	Free aCTLA-4 vs. aCTLA-4 micelle	0.8697
	Free aCTLA-4 vs. aCTLA-4 hydrogel	0.0742
	aCTLA-4 micelle vs. aCTLA-4 hydrogel	0.0234
**i NK**	Free aCTLA-4 vs. aCTLA-4 micelle	0.9763
	Free aCTLA-4 vs. aCTLA-4 hydrogel	0.9957
	aCTLA-4 micelle vs. aCTLA-4 hydrogel	0.0228
	Free aCTLA-4 vs. aCTLA-4 micelle	0.9985
	Free aCTLA-4 vs. aCTLA-4 hydrogel	0.0103
	aCTLA-4 micelle vs. aCTLA-4 hydrogel	0.0212
**i NKT**	Free aCTLA-4 vs. aCTLA-4 micelle	0.9924
	Free aCTLA-4 vs. aCTLA-4 hydrogel	0.9926
	aCTLA-4 micelle vs. aCTLA-4 hydrogel	0.0251
	Free aCTLA-4 vs. aCTLA-4 micelle	0.9497
	Free aCTLA-4 vs. aCTLA-4 hydrogel	0.0147
	aCTLA-4 micelle vs. aCTLA-4 hydrogel	0.0600

Supplementary Table 3. Summary of concentration dependent sol-gel and gel-sol transition of F127® and F127-*g*-Gelatin.

**wt%**	**Sol→Gel T (ºC) / Gel→Sol T (ºC)**		**Final F127 wt% in F127-*g*-Gelatin**
	F127	F127-*g*-Gelatin	
0.5	Always sol (4–85 ˚C)	Always sol (4–85 ˚C)	0.32
1			0.65
1.5			0.97
2			1.29
2.5			1.62

3			1.94
3.5			2.26
4		32 ˚C / Not Available (N.A.)	2.59
4.5		31 ˚C / N.A.	2.91
5		30 ˚C / N.A.	3.24
5.5		30 ˚C / N.A	3.56
6		30 ˚C / N.A	3.88
6.5		29 ˚C / N.A	4.21
7		29 ˚C / N.A	4.53
7.5		Always gel (4–85 ˚C)	4.85
10			6.47
15			9.71
17.5	38 ˚C / 47 ˚C		11.32
20	28 ˚C / 64 ˚C		12.94
22.5	26 ˚C / 70 ˚C		14.56
25	22 ˚C / 77 ˚C		16.18

Supplementary Table 4. Summary of concentration dependent sol-gel and gel-sol transition of Gelatin and the mixture of Gelatin and F127 (Gelatin:F127=35.1 wt.%:64.9 wt.%).

wt%	Gelatin Gel→Sol T (ºC) / Sol→Gel T (ºC)	wt%	F127/Gelatin mixture (64.9%:35.1%) Gel→Sol T (ºC) / Sol→Gel T (ºC)
0.2	Always sol (4–85 ˚C)	1	Always sol (4–85 ˚C)
0.4		2	
0.6		4	25 ˚C / N.A.
0.8	24 ˚C / N.A.	5	28 ˚C / N.A.
1	25 ˚C / N.A.	6	28 ˚C / N.A.
1.2	28 ˚C / N.A.	7	28 ˚C / N.A.
1.4	31 ˚C / N.A.	8	Not dissolved
1.6	32 ˚C / N.A.	10	
1.8	33 ˚C / N.A.	12.5	
2	33 ˚C / N.A.	15	
2.2	33 ˚C / N.A.	>17.5	
2.5	36 ˚C / N.A.		
3	36 ˚C / N.A.		

4	37 ˚C / N.A.		
5	38 ˚C / N.A.		
> 5.5	Not dissolved in 25 ˚C		

Supplementary Table 5. Summary of CMC of F127, mixture of Gelatin and F127 (Gelatin:F127=35.1 wt.%:64.9 wt.%) and F127-*g*-Gelatin at RT and 37 ˚C.

		F127	Mixture	F127-*g*-Gelatin	Fold (F127/F127-*g*-Gelatin)
CMC_1 (mg mL^{-1})	25 ºC	3.09±1.53	4.45±0.54	0.98±0.24	3.15
	37 ºC	0.044±0.0085	0.095±0.013	0.0085±0.0022	5.17
Fold (25 ºC/37 ºC)		70.2	46.8	115.3	

Supplementary Table 6. Summary of CMC_1 and CMC_2 of F127-*g*-Gelatin with or without aCTLA-4 and aCTLA-4-AF647 at 37 ˚C.

At 37 ºC	w/o additive	w/ aCTLA-4	w/ aCTLA-4-AF647
CMC_1 (mg mL^{-1})	0.0085±0.0022	0.014±0.0040	0.0037±0.0018
CMC_2 (mg mL^{-1})	None	0.00042±0.00027	0.00011±0.000041

Supplementary Table 7. Statistical comparison for Fig. 5 with one-way ANOVA using Tukey post-hoc statistical hypothesis.

Figures	*vs.*	***P* value**		
		day 1	day 7	day 11
**a**	Free aCTLA-4 vs. aCTLA-4 micelle	0.9974	0.4023	0.6266
	Free aCTLA-4 vs. aCTLA-4 hydrogel	0.0009	0.0016	0.1035
	aCTLA-4 micelle vs. aCTLA-4 hydrogel	0.0010	0.0080	0.0176
**b**	Free aCTLA-4 vs. aCTLA-4 micelle	0.0215	0.0543	0.2064

	Free aCTLA-4 vs. aCTLA-4 hydrogel	0.0753	0.1861	0.6534
	aCTLA-4 micelle vs. aCTLA-4 hydrogel	0.7092	0.7066	0.5666
**c**	Free aCTLA-4 vs. aCTLA-4 micelle	0.8605	0.9903	0.9093
	Free aCTLA-4 vs. aCTLA-4 hydrogel	0.84277	0.2455	0.8804
	aCTLA-4 micelle vs. aCTLA-4 hydrogel	0.9993	0.2036	0.9971
**d**	Free aCTLA-4 vs. aCTLA-4 micelle	0.6298	0.9980	0.0122
	Free aCTLA-4 vs. aCTLA-4 hydrogel	0.6552	0.0154	0.0098
	aCTLA-4 micelle vs. aCTLA-4 hydrogel	0.2120	0.0169	0.9826
**e**	Free aCTLA-4 vs. aCTLA-4 micelle	0.6436	0.9784	0.4783
	Free aCTLA-4 vs. aCTLA-4 hydrogel	0.6057	0.0834	0.1632
	aCTLA-4 micelle vs. aCTLA-4 hydrogel	0.1943	0.0612	0.6560
**f**	Free aCTLA-4 vs. aCTLA-4 micelle	0.0065	0.9790	0.9354
	Free aCTLA-4 vs. aCTLA-4 hydrogel	0.4705	0.1752	0.9930
	aCTLA-4 micelle vs. aCTLA-4 hydrogel	0.0012	0.2321	0.8737
**g**	Free aCTLA-4 vs. aCTLA-4 micelle	0.0228	0.1175	0.4067
	Free aCTLA-4 vs. aCTLA-4 hydrogel	0.7475	0.0369	0.6324
	aCTLA-4 micelle vs. aCTLA-4 hydrogel	0.0720	0.7424	0.8961
**h**	Free aCTLA-4 vs. aCTLA-4 micelle	0.0418	0.8897	0.2978
	Free aCTLA-4 vs. aCTLA-4 hydrogel	0.8764	0.2058	0.9969
	aCTLA-4 micelle vs. aCTLA-4 hydrogel	0.0195	0.1034	0.2800
**i**	Free aCTLA-4 vs. aCTLA-4 micelle	0.6842	0.9328	0.0848
	Free aCTLA-4 vs. aCTLA-4 hydrogel	0.3460	0.1168	0.0305
	aCTLA-4 micelle vs. aCTLA-4 hydrogel	0.1031	0.0675	0.7404

Supplementary Table 8. Statistical comparison for Fig. 6d with Log-rank analysis with Mantel-Cox statistical hypothesis.

vs.	***P* value**
Saline vs. Free GSNO	0.7589
Saline vs. Free aCTLA	0.1047
Saline vs. Free GSNO+aCTLA-4	0.0027
Saline vs. HG	0.7012
Saline vs. GSNO/HG	0.5261
Saline vs. aCTLA-4/HG	0.0953
Saline vs. GSNO+aCTLA-4/HG	0.0027
Free GSNO vs. Free aCTLA	0.1627

Free GSNO vs. Free GSNO+aCTLA-4	0.0027
Free GSNO vs. HG	0.9787
Free GSNO vs. GSNO/HG	0.7839
Free GSNO vs. aCTLA-4/HG	0.1276
Free GSNO vs. GSNO+aCTLA-4/HG	0.0027
Free aCTLA-4 vs. Free GSNO+aCTLA-4	0.0198
Free aCTLA-4 vs. HG	0.2348
Free aCTLA-4 vs. GSNO/HG	0.2691
Free aCTLA-4 vs. aCTLA-4/HG	0.4065
Free aCTLA-4 vs. GSNO+aCTLA-4/HG	0.0064
Free GSNO+aCTLA-4 vs. HG	0.0018
Free GSNO+aCTLA-4 vs. GSNO/HG	0.0019
Free GSNO+aCTLA-4 vs. aCTLA-4/HG	0.2712
Free GSNO+aCTLA-4 vs. GSNO+aCTLA-4/HG	0.5402
HG vs. GSNO/HG	0.9854
HG vs. aCTLA-4/HG	0.0800
HG vs. GSNO+aCTLA-4/HG	0.0018
GSNO/HG vs. aCTLA-4/HG	0.1120
GSNO/HG vs. GSNO+aCTLA-4/HG	0.0019
aCTLA-4/HG vs. GSNO+aCTLA-4/HG	0.0244

Supplementary Table 9. Statistical comparison for Fig. 6e, f with ANOVA using linear mixed-effects model.

vs.	***P* value**	
	1º tumor	2º tumor
Saline vs. Free GSNO	0.9997	>0.9999
Saline vs. Free aCTLA-4	0.9888	0.7044
Saline vs. Free GSNO+aCTLA-4	0.0865	0.0106
Saline vs. HG	>0.9999	0.9998
Saline vs. GSNO/HG	0.9993	>0.9999
Saline vs. aCTLA-4/HG	0.9223	0.2843
Saline vs. GSNO+aCTLA-4/HG	0.0003	0.0015
Free GSNO vs. Free aCTLA-4	>0.9999	0.7166
Free GSNO vs. Free GSNO+aCTLA-4	0.2215	0.0101
Free GSNO vs. HG	>0.9999	0.9992
Free GSNO vs. GSNO/HG	>0.9999	>0.9999
Free GSNO vs. aCTLA-4/HG	0.9951	0.2852
Free GSNO vs. GSNO+aCTLA-4/HG	0.0012	0.0014
Free aCTLA-4 vs. Free GSNO+aCTLA-4	0.3987	0.3506
Free aCTLA-4 vs. HG	0.9979	0.3733
Free aCTLA-4 vs. GSNO/HG	>0.9999	0.7756
Free aCTLA-4 vs. aCTLA-4/HG	>0.9999	0.9952
Free aCTLA-4 vs. GSNO+aCTLA-4/HG	0.0086	0.0919
Free GSNO+aCTLA-4 vs. HG	0.1320	0.0022

Free GSNO+aCTLA-4 vs. GSNO/HG	0.2448	0.0130
Free GSNO+aCTLA-4 vs. aCTLA-4/HG	0.6253	0.7900
Free GSNO+aCTLA-4 vs. GSNO+aCTLA-4/HG	0.3888	0.9951
HG vs. GSNO/HG	>0.9999	0.9974
HG vs. aCTLA-4/HG	0.9699	0.0979
HG vs. GSNO+aCTLA-4/HG	0.0006	0.0003
GSNO/HG vs. aCTLA-4/HG	0.9970	0.3352
GSNO/HG vs. GSNO+aCTLA-4/HG	0.0014	0.0019
aCTLA-4/HG vs. GSNO+aCTLA-4/HG	0.0086	0.3534

Supplementary Table 10. Statistical comparison for Supplementary Fig. 41 with ANOVA using linear mixed-effects model.

vs.	***P* value**	
	1° tumor	2° tumor
Saline vs. Fig. 3	0.6078	0.0110
Saline vs. Fig. 6	0.0067	<0.0001
Fig.3 vs. Fig. 6	0.0380	0.0099

Supplementary Table 11. Statistical comparison for Fig. 7c with Log-rank analysis with Mantel-Cox statistical hypothesis.

vs.	***P* value**
Saline vs. HG	0.9375
Saline vs. Free GSNO+aCTLA-4	0.2818
Saline vs. GSNO+aCTLA-4/HG	0.0183
HG vs. Free GSNO+aCTLA-4	0.3452
HG vs. GSNO+aCTLA-4/HG	0.0208
Free GSNO+aCTLA-4 vs. GSNO+aCTLA-4/HG	0.5226

Supplementary Table 12. Statistical comparison for Fig. 7d, e with ANOVA using linear mixed-effects model.

vs.	***P* value**	
	1° tumor	2° tumor
Saline vs. HG	0.9822	>0.9999

Saline vs. Free GSNO+aCTLA-4	0.7800	0.1479
Saline vs. GSNO+aCTLA-4/HG	0.0021	<0.0001
HG vs. Free GSNO+aCTLA-4	0.9392	0.1648
HG vs. GSNO+aCTLA-4/HG	0.0061	0.0001
Free GSNO+aCTLA-4 vs. GSNO+aCTLA-4/HG	0.0281	0.0400

Supplementary Table 13. Antibody list for Fig. 1, and Supplementary Fig. 1-19.

Color	Antibody	Clone	Company (Cat.#)	Dilution
FITC	CD11c	N418	Biolegend (117306)	1.25:100
PerCP	CD45	30-F11	Biolegend (103130)	1.25:100
PE	CD86	GL-1	Biolegend (105008)	1.25:100
PE/Cy7	CD4	GK1.5	Biolegend (100422)	1.25:100
APC	GR1	RB6-8C5	Biolegend (108412)	1.25:100
AF700	CD11b	M1/70	Biolegend (101222)	1.25:100
APC/Cy7	MHCII	M5/114.15.2	Biolegend (107628)	1.25:100
BV421	FoxP3	MF-14	Biolegend (126419)	2.5:100
BV605	CTLA-4 (CD152)	UC10-4B9	Biolegend (106323)	2.5:100
BV711	CD3	145-2C11	Biolegend (100349)	1.25:100
BV785	F4/80	BM8	Biolegend (123141)	2.5:100
Zombie Aqua (BUV 496)			Biolegend (423102)	1:100

Supplementary Table 14. Antibody list for Fig. 2g-j, and Supplementary Fig. 22.

Color	Antibody	Clone	Company	Dilution
FITC	CRT	1G6A7	NOVUS Biologicals (NBP1-47518F)	1:100
PerCP	CD45	30-F11	Biolegend (103130)	1.25:100
APC	PD-1	RMP1-30	Biolegend (109112)	1.25:100
AF700	Ki-67	16A8	Biolegend (652420)	2.5:100
BV605	CTLA-4 (CD152)	UC10-4B9	Biolegend (106323)	2.5:100
BV785	PD-L1	10F.9G2	Biolegend (124331)	2.5:100

Zombie Aqua (BUV 496)	Biolegend (423102)	1:100

Supplementary Table 15. Antibody list for Fig. 3f-i.

Color	Antibody	Clone	Company	Dilution
FITC	CD4	GK1.5	Biolegend (100406)	1.25:100
PerCP	CD45	30-F11	Biolegend (103130)	1.25:100
PE	SIINFEKL-MHCI–PE		NIH Tetramer Core Facility	1:100
APC	LAG-3	C9B7W	Biolegend (125210)	5:100
AF700	CD25	PC61	Biolegend (102024)	0.5:100
APC/Cy7	CD8	53-6.7	Biolegend (100714)	2.5:100
BV421	FoxP3	MF-14	Biolegend (126419)	2.5:100
BV605	NK1.1	PK136	Biolegend (108740)	2.5:100
BV711	CD3	145-2C11	Biolegend (100349)	1.25:100
BV785	PD-1	29F.1A12	Biolegend (135225)	1.25:100
Zombie Aqua (BUV 496)			Biolegend (423102)	1:100
