# Supplementary material for: Thermosensitive hydrogel releasing nitric oxide donor and anti-CTLA-4 micelles for anti-tumor immunotherapy
Source: Nat Commun. 2022 Mar 18;13:1479. doi: 10.1038/s41467-022-29121-x (PMC8933465; doi:10.1038/s41467-022-29121-x)
Supplement: Supplementary file 2 — Reporting Summary [file 41467_2022_29121_MOESM2_ESM.pdf]

nature research

Corresponding author(s): Susan N. Thomas, NCOMMS-21-10753C

Last updated by author(s): Feb 15, 2022

Reporting Summary

Nature Research wishes to improve the reproducibility of the work that we publish. This form provides structure for consistency and transparency in reporting. For further information on Nature Research policies, see our Editorial Policies and the Editorial Policy Checklist.

Statistics

For all statistical analyses, confirm that the following items are present in the figure legend, table legend, main text, or Methods section.

n/a	Confirmed	
☐	☒	The exact sample size (n) for each experimental group/condition, given as a discrete number and unit of measurement
☐	☒	A statement on whether measurements were taken from distinct samples or whether the same sample was measured repeatedly
☐	☒	The statistical test(s) used AND whether they are one- or two-sided<br>*Only common tests should be described solely by name; describe more complex techniques in the Methods section.*
☐	☒	A description of all covariates tested
☐	☒	A description of any assumptions or corrections, such as tests of normality and adjustment for multiple comparisons
☐	☒	A full description of the statistical parameters including central tendency (e.g. means) or other basic estimates (e.g. regression coefficient) AND variation (e.g. standard deviation) or associated estimates of uncertainty (e.g. confidence intervals)
☐	☒	For null hypothesis testing, the test statistic (e.g. F, t, r) with confidence intervals, effect sizes, degrees of freedom and P value noted<br>*Give P values as exact values whenever suitable.*
☒	☐	For Bayesian analysis, information on the choice of priors and Markov chain Monte Carlo settings
☒	☐	For hierarchical and complex designs, identification of the appropriate level for tests and full reporting of outcomes
☒	☐	Estimates of effect sizes (e.g. Cohen's d, Pearson's r), indicating how they were calculated

Our web collection on statistics for biologists contains articles on many of the points above.

Software and code

Policy information about availability of computer code

Data collection	Proton NMR was collected by Bruker Advance 400 MHz FT-NMR with Topspin v3.0 softwre. Differential Scanning Calorimetry data were collected by TA Instruments Q200 with Advantage v2.0 software. X-ray diffraction was collected by Malvern PANalytical Empyrean with Highscore v4.9 software. Rheology was collected by MCR 302 rheometer with Rheocompass v1.24.584 software. Scanning electron microscopy images were collected by Hitachi SU-8230. High-resolution FEI Tecnai G2 F30 TEM with Gatan GMS v3.2 software was used to take transmission electron microscopy images of micelles. Malvern Zetasizer Nano ZS with Zetasizer software v5.1 was used to collect size and zeta potential of micelles. All fluorescence and absorbance data were collected by Synergy H4 microplate reader with Gen5 v2.09 software. Flow cytometry data were collected by BD Fortessa with BD FACSDiva v9.0 software. H&E stained tissues were imaged using Nanozoomer 2.0 HT.
Data analysis	GraphPad Prism 9 and R studio 1.2.5033 with lme4 (v1.1.26) and emmeans package (v.1.7.2) for graph formatting and statistical analysis. MestreNova NMR v11 for NMR analysis. FlowJo v10.6 for all flow cytometry analysis.

For manuscripts utilizing custom algorithms or software that are central to the research but not yet described in published literature, software must be made available to editors and reviewers. We strongly encourage code deposition in a community repository (e.g. GitHub). See the Nature Research guidelines for submitting code & software for further information.

Data

Policy information about availability of data

All manuscripts must include a data availability statement. This statement should provide the following information, where applicable:

- Accession codes, unique identifiers, or web links for publicly available datasets
- A list of figures that have associated raw data
- A description of any restrictions on data availability

The source data supporting this study's findings are available with this paper. The remaining information are available within the Article, Supplementary Information or Source Data file. Other data and analysis that may not be included in this article, supplementary information and source files are available from the first and corresponding authors on reasonable request.

Field-specific reporting

Please select the one below that is the best fit for your research. If you are not sure, read the appropriate sections before making your selection.

☒ Life sciences ☐ Behavioural & social sciences ☐ Ecological, evolutionary & environmental sciences

For a reference copy of the document with all sections, see nature.com/documents/nr-reporting-summary-flat.pdf

Life sciences study design

All studies must disclose on these points even when the disclosure is negative.

Sample size	There were no statistical methods to predetermine the sample sizes. The group sizes (n=3-12) were determined based on our experimental experience, which are indicated in each figure and were enough to facilitate the statistical analysis.
Data exclusions	No data were excluded.
Replication	Experiments were repeated at least in triplicate, which findings were reproducible.
Randomization	All experimental samples, cells, and animals were randomly allocated into experimental groups.
Blinding	The investigators were not blinded during experiments and data analysis because most of studies require multiple processing steps that scientists must carefully track.

Reporting for specific materials, systems and methods

We require information from authors about some types of materials, experimental systems and methods used in many studies. Here, indicate whether each material, system or method listed is relevant to your study. If you are not sure if a list item applies to your research, read the appropriate section before selecting a response.

Materials & experimental systems

n/a	Involved in the study	
☐	☒	Antibodies
☐	☒	Eukaryotic cell lines
☒	☐	Palaeontology and archaeology
☐	☒	Animals and other organisms
☒	☐	Human research participants
☒	☐	Clinical data
☒	☐	Dual use research of concern

Methods

n/a	Involved in the study	
☒	☐	ChIP-seq
☐	☒	Flow cytometry
☒	☐	MRI-based neuroimaging

Antibodies

Antibodies used

All is monoclonal antibodies.
1. CD11c-FITC (Biolegend, Clone: N418, Catalogue #: 117306, dilution: 1.25:100)
2. CD45-PerCP (Biolegend, Clone: 30-F11, Catalogue #: 103130, dilution: 1.25:100)
3. CD86-PE (Biolegend, Clone: GL-1, Catalogue #: 105008, dilution: 1.25:100)
4. CD4-PE/Cy7 (Biolegend, Clone: GK1.5, Catalogue #: 100422, dilution: 1.25:100)
5. GR1-APC (Biolegend, Clone: RB6-8C5, Catalogue #: 108412, dilution: 1.25:100)
6. CD11b-AF700 (Biolegend, Clone: M1/70, Catalogue #: 101222, dilution: 1.25:100)
7. MHCII-APC/Cy7 (Biolegend, Clone: M5/114.15.2, Catalogue #: 107628, dilution: 1.25:100)
8. FoxP3-BV421 (Biolegend, Clone: MF-14, Catalogue #: 126419, dilution: 2.5:100)

9. CTLA-4-BV605 (Biolegend, Clone: UC10-4B9, Catalogue #: 106323, dilution: 2.5:100)
10. CD3-BV711 (Biolegend, Clone: 145-2C11, Catalogue #: 100349, dilution: 1.25:100)
11. F4/80-BV785 (Biolegend, Clone: BM8, Catalogue #: 123141, dilution: 2.5:100)
12. CRT-FITC (NOVUS Biologicals, Clone: 1G6A7, Catalogue #: NBP1-47518F, dilution: 1:100)
13. PD-1-APC (Biolegend, Clone: RMP1-30, Catalogue #: 109112, dilution: 1.25:100)
14. Ki-67-AF700 (Biolegend, Clone: 16A8, Catalogue #: 652420, dilution: 2.5:100)
15. PD-L1-BV785 (Biolegend, Clone: 10F.9G2, Catalogue #: 124331, dilution: 2.5:100)
16. CD4-FITC (Biolegend, Clone: GK1.5, Catalogue #: 100406, dilution: 1.25:100)
17. SIINFEKL-MHCI-PE (NIH Tetramer Core Facility, dilution: 1:100)
18. LAG-3-APC (Biolegend, Clone: C9B7W, Catalogue #:125210, dilution: 5:100)
19. CD25-AF700 (Biolegend, Clone: PC61, Catalogue #: 102024, dilution: 0.5:100)
20. CD8-APC/Cy7 (Biolegend, Clone: 53-6.7,Catalogue #: 100714, dilution: 2.5:100)
21. NK1.1-BV605 (Biolegend, Clone: PK136, Catalogue #: 108740, dilution: 2.5:100)
22. PD-1-BV785 (Biolegend, Clone: 29F.1A12, Catalogue #: 135225, dilution: 1.25:100)

Validation

All antibodies were verified by the supplier as below;
1. https://www.biolegend.com/en-us/products/fitc-anti-mouse-cd11c-antibody-1815
2. https://www.biolegend.com/en-us/products/percp-anti-mouse-cd45-antibody-4265
3. https://www.biolegend.com/en-us/products/pe-anti-mouse-cd86-antibody-256
4. https://www.biolegend.com/en-us/products/pe-cyanine7-anti-mouse-cd4-antibody-1919
5. https://www.biolegend.com/en-us/products/apc-anti-mouse-ly-6g-ly-6c-gr-1-antibody-456
6. https://www.biolegend.com/en-us/products/alexa-fluor-700-anti-mouse-human-cd11b-antibody-3388
7. https://www.biolegend.com/en-us/products/apc-cyanine7-anti-mouse-i-a-i-e-antibody-5966
8. https://www.biolegend.com/en-us/products/brilliant-violet-421-anti-mouse-foxp3-antibody-12143
9. https://www.biolegend.com/en-us/products/brilliant-violet-605-anti-mouse-cd152-antibody-12375
10. https://www.biolegend.com/en-us/products/brilliant-violet-711-anti-mouse-cd3epsilon-antibody-11975
11. https://www.biolegend.com/en-us/products/brilliant-violet-785-anti-mouse-f4-80-antibody-9919
12. https://www.novusbio.com/products/calreticulin-antibody-1g6a7_nbp1-47518f
13. https://www.biolegend.com/en-us/products/apc-anti-mouse-cd279-pd-1-antibody-6672
14. https://www.biolegend.com/en-us/products/alexa-fluor-700-anti-mouse-ki-67-antibody-10366
15. https://www.biolegend.com/en-us/products/brilliant-violet-785-anti-mouse-cd274-b7-h1-pd-l1-antibody-13497
16. https://www.biolegend.com/en-us/products/fitc-anti-mouse-cd4-antibody-248
17. https://tetramer.yerkes.emory.edu
18. https://www.biolegend.com/en-us/products/apc-anti-mouse-cd223-lag-3-antibody-6926
19. https://www.biolegend.com/en-us/products/alexa-fluor-700-anti-mouse-cd25-antibody-3389
20. https://www.biolegend.com/en-us/products/apc-cyanine7-anti-mouse-cd8a-antibody-2269
21. https://www.biolegend.com/en-us/products/brilliant-violet-605-anti-mouse-nk-1-1-antibody-8665
22. https://www.biolegend.com/en-us/products/brilliant-violet-785-anti-mouse-cd279-pd-1-antibody-9874

Eukaryotic cell lines

Policy information about cell lines

Cell line source(s)

B16F10-OVA mouse melanoma, 4T1 mouse breast tumor, and NIH3T3 mouse fibroblast cells were provided from Prof. Melody Swartz previously at École Polytechnique Fédérale de Lausanne, Prof. Edmund Waller in Emory University, and Prof. Andres Garcia in Georgia Institute of Technology, respectively.

Authentication

Cells were not authenticated after receipt.

Mycoplasma contamination

Cell lines were not tested for mycoplasma contamination

Commonly misidentified lines
(See ICLAC register)

There are no commonly misidentified cell lines in this study.

Animals and other organisms

Policy information about studies involving animals; ARRIVE guidelines recommended for reporting animal research

Laboratory animals

All animal procedures were IACUC approved with an A100305 protocol and performed in Georgia Tech's Physiological Research Laboratory (PRL). C57Bl/6 and Balb/C mice were purchased from Jackson Laboratories and 8-10 weeks old C57Bl/6 female mice & 6-12 weeks old Balb/C female mice were used for experiments.
Mice were housed in ventilated cage (max 5 mice/cage) supplied with food and water in a 12-h light/12-h dark cycle (7:00-19:00 light & 19:00 pm-7:00 dark) with 22 ˚C and 41% humidity

Wild animals

This study does not contain any wild animals.

Field-collected samples

This study does not involve any samples collected from the field.

Ethics oversight

All animal procedures were IACUC approved and performed in Georgia Institute of Technology's Physiological Research Laboratory (A100379 & A100305).

Note that full information on the approval of the study protocol must also be provided in the manuscript.

Flow Cytometry

Plots

Confirm that:

- [x] The axis labels state the marker and fluorochrome used (e.g. CD4-FITC).
- [x] The axis scales are clearly visible. Include numbers along axes only for bottom left plot of group (a 'group' is an analysis of identical markers).
- [x] All plots are contour plots with outliers or pseudocolor plots.
- [x] A numerical value for number of cells or percentage (with statistics) is provided.

Methodology

Sample preparation	B16F10-OVA mouse melanoma and 4T1 mouse breast tumor were passaged in Dulbecco's Modified Eagle Medium (GibcoTM, DMEM) containing 10% Fetal Bovine Serum (GibcoTM, FBS) and 1X Antibiotic-Antimycotic (GibcoTM) in CO2 incubator at 37 ˚C. To develop tumor model, B16F10-OVA cells or 4T1 cells were inoculated to left dorsal of C57Bl/6 or mammary fatpad of Balb/C,respectively.<br>Tissues of interest were harvested from mice by surgery after sacrificing the mice. Cells in tumor, spleen, lymph nodes were harvested by using 70 µm strainer. Detailed sampling method for immune profiles are described in the Method sections
Instrument	BD Fortessa was used to collect the flow cytometry data.
Software	FlowJo 10.6.2 was used to analyze the flow cytometry data
Cell population abundance	After gating the populations, both the number and percentage of gated cells were reported.
Gating strategy	The detailed gating strategy can be found in supplementary information. In brief, fluorescence were first compensated with FlowJo program. Cells were gated on FSC-A/SSC-A, singlet cells were gated on FSC-A/FSC-H, live singlet cells were gated on negative population in live dead, and then CD45 positive population for immune cell and CD45 negative population were gated by CD45 signal. After gating immune cells and non-immune cells, each antibody was used to gate specific populations. Detailed gating strategies are shown in Supplementary Figure 1 and 22.

- [x] Tick this box to confirm that a figure exemplifying the gating strategy is provided in the Supplementary Information.
